# Benzimidazole derivatives as dual EGFR and BRAF^V600E^ inhibitors with pro-apoptotic antiproliferative potential

**DOI:** 10.1039/d6ra02117d

**Published:** 2026-05-14

**Authors:** Lamya H. Al-Wahaibi, Mohamed Samir, Stefan Bräse, Bahaa G. M. Youssif, Martha M. Morcoss

**Affiliations:** a Department of Chemistry, College of Sciences, Princess Nourah Bint Abdulrahman University Riyadh 11671 Saudi Arabia; b Department of Pharmaceutical Organic Chemistry, Faculty of Pharmacy, Al-Azhar University 71524 Assiut Egypt; c Institute of Biological and Chemical Systems, IBCS-FMS, Karlsruhe Institute of Technology 76131 Karlsruhe Germany braese@kit.edu; d Department of Pharmaceutical Organic Chemistry, Faculty of Pharmacy, Assiut University Assiut-71526 Egypt bgyoussif2@gmail.com +20-2-01044353895; e Department of Pharmaceutical Chemistry, Faculty of Pharmacy, Nahda University 62513 Beni-Suef Egypt

## Abstract

Resistance to single-target kinase inhibitors is increasing, necessitating the development of multi-target-directed medications that can simultaneously affect different carcinogenic pathways. The objective of this investigation was to develop, synthesize, and evaluate the pharmacological properties of a novel set of compounds that are derived from benzimidazole and thereby inhibit both EGFR and BRAF^V600E^. Compounds 8c, 8d, and 8f were identified as the most potent candidates in *in vitro* enzymatic and antiproliferative investigations. Compound 8d exhibited the most potent dual inhibitory activity, with IC_50_ values of 7.17 nM against EGFR and 45.50 nM against BRAF^V600E^. These values were comparable to those of the reference medications vemurafenib (IC_50_ = 41.38 nM) and erlotinib (IC_50_ = 5.40 nM). Mechanistic studies have shown that compounds 8d and 8f strongly induce apoptosis, primarily through the intrinsic (mitochondrial) pathway, as evidenced by significant activation of caspase-9 and caspase-3. The experimental results were confirmed by molecular docking studies, which showed that compound 8d exhibits strong binding affinity for both the EGFR and BRAF^V600E^ kinase domains. The *in silico* ADME profiling and drug-likeness analysis revealed that compound 8d exhibits high gastrointestinal absorption and adheres to Lipinski's rule of five, supporting its potential as an oral therapy.

## Abstract 2

Compound 8d exhibited potent dual inhibitory activity against EGFR and BRAF^V600E^. It induces apoptosis, with high gastrointestinal absorption.

## Graphical abstract

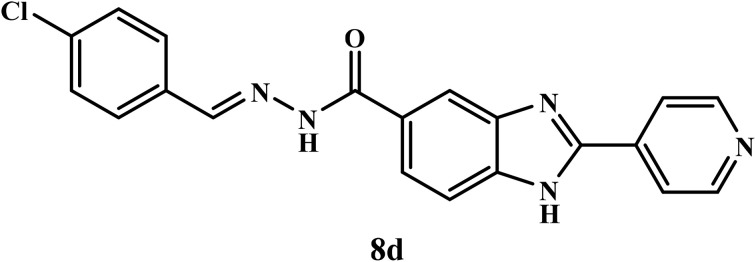

## 1. Introduction

Cancer is one of the main causes of death throughout the world because it causes cells to grow out of control, and its genetic makeup changes all the time.^1^ For many years, the most common way to treat cancer has been with standard chemotherapy. However, it is usually characterized by a narrow therapeutic window and a lack of selectivity, which can lead to severe side effects and make it harder to overcome multidrug resistance.^2,3^ Contemporary oncology has evolved into a precision medicine paradigm that employs high-throughput genome sequencing to detect oncogenic mutations, including BRAF^V600E^. This change has made it possible to develop personalized drugs and immunotherapies that work better.^4,5^ However, cancer can employ several signalling pathways and adaptive feedback mechanisms, presenting a considerable obstacle for therapy. Consequently, current studies emphasize novel synthetic strategies that concurrently block multiple routes within the signalling network, thereby preventing the development of acquired resistance.^6,7^

A prime example of this challenge is shown in BRAF^V600E^-mutant metastatic colorectal cancer (mCRC). BRAF inhibitors have revolutionized melanoma treatment; however, their effectiveness in mCRC is limited by an adaptive feedback reactivation mechanism.^8,9^ In these cells, rapid inhibition of BRAF increases Epidermal Growth Factor Receptor (EGFR) levels, thereby bypassing the blockade and restoring MAPK/ERK signaling.^10,11^ The Phase III BEACON CRC trial (NCT02928224)^12,13^ showed that using encorafenib (a BRAF^V600E^ inhibitor) and cetuximab (a monoclonal antibody targeting EGF00R) together to block two pathways significantly improved survival compared with single-agent treatment. This dual-target approach has been approved as standard treatment by the FDA (Food and Drug Administration) and the European Medicines Agency (EMA).^14,15^ This technique is being tested in a variety of settings, including non-small cell lung cancer (NSCLC), where BRAF mutations can develop as an acquired resistance mechanism to EGFR inhibitors such as osimertinib.^16^ Current treatments aim to convert a temporary response into a long-term therapeutic benefit by suppressing both the primary carcinogenic driver and its primary escape mechanism.^17,18^

Benzimidazole-derived compounds, owing to their structural resemblance to natural purines and nucleosides, constitute one of the most diverse and thoroughly investigated small-molecule frameworks in cancer therapy.^19,20^ This “privileged” scaffold enables engagement with a variety of biological targets associated with cancer growth. Benzimidazole compounds exhibit anticancer activity through a variety of pathways, including kinase inhibition of BRAF, CDK4/6, EGFR, and VEGFR-2.^21–24^

**Fig. 1:**
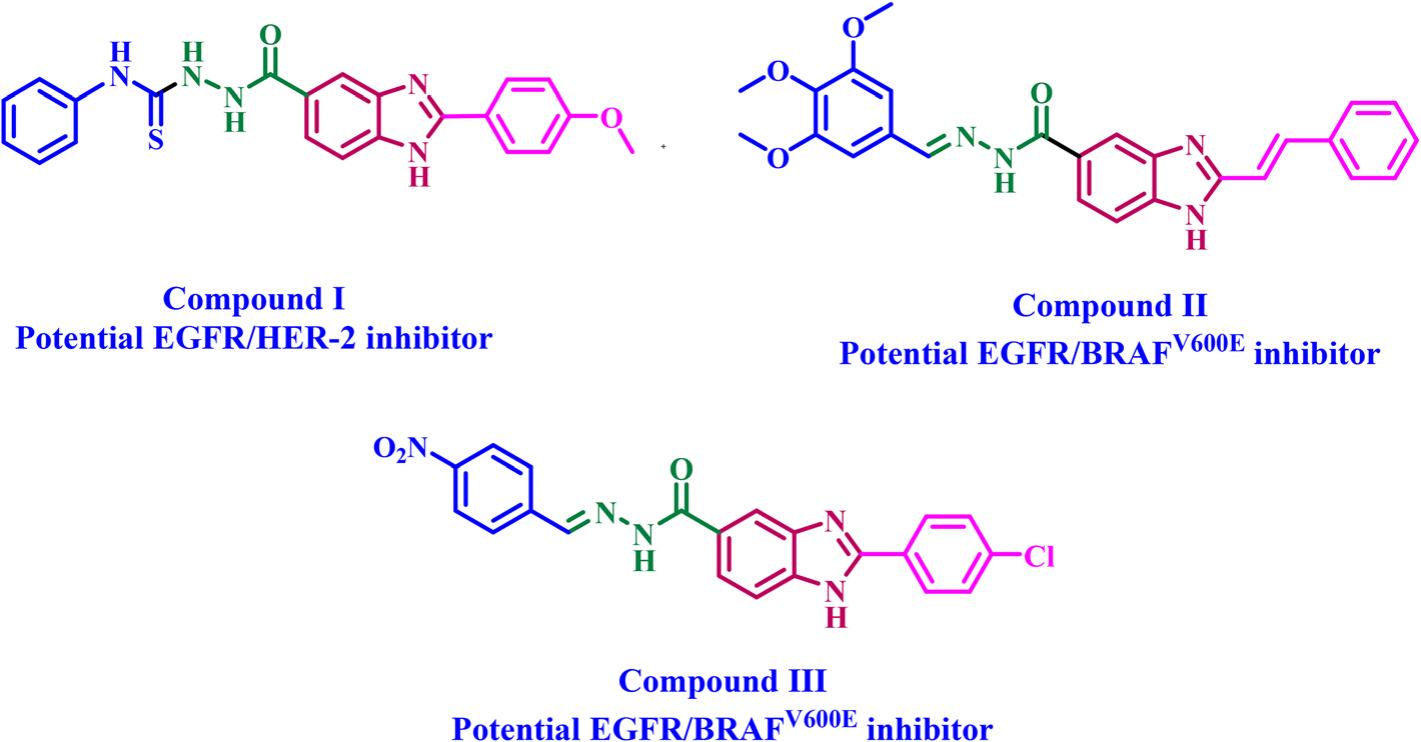
Structures of some reported benzimidazoles as kinase inhibitors (I–III).

We recently^25^ reported the design, synthesis, and antiproliferative efficacy of a novel series of benzimidazole-based compounds that function as dual inhibitors of EGFR and HER-2. Compound I (Fig. 1) demonstrated enhanced activity against the MCF-7 breast cancer cell line, with an IC_50_ of 5 µM, nearly six times more potent than Doxorubicin. Compound I had the greatest activity as an EGFR inhibitor, with an IC_50_ of 76 nM, comparable to the reference erlotinib (IC_50_ = 80 nM). Compound I was identified as the most potent HER-2 inhibitor, with an IC_50_ of 60 nM, while the reference lapatinib exhibited an IC_50_ of 26 nM. Compound I demonstrated a similar interaction with the ATP-binding site of the EGFR tyrosine kinase. It established robust interactions with the Met769 critical residue in the binding site, exhibiting an overall binding affinity of −8.9 kcal mol^−1^, in contrast to the positive control erlotinib, which demonstrated a binding score of −8.3 kcal mol^−1^.

In another paper,^26^ a novel set of styryl benzimidazole derivatives was developed, synthesized, and tested as antiproliferative agents that inhibit both EGFR and BRAF^V600E^. Compound II (Fig. 1) has the highest antiproliferative efficacy. Compound II effectively inhibited EGFR (IC_50_ = 79 ± 5 nM) and BRAF^V600E^ (IC_50_ = 77 nM), with IC_50_ values comparable to those of the reference medicines erlotinib and vemurafenib. Apoptotic marker assays (Caspases, Bax, Bcl-2, and p53) revealed that apoptosis contributes to the antiproliferative effects. Molecular docking showed that compound II favorably accommodated within the ATP-binding pocket of EGFR, with an interaction energy of −8.40 kcal mol^−1^, supporting its proposed mechanism of action.

A novel class of benzimidazole compounds exhibiting potential dual inhibition of EGFR and BRAF^V600E^ has been synthesised.^27^ The newly synthesised compounds were tested for antiproliferative activity against the NCI-60 cell line. All newly synthesised compounds were evaluated against a panel of 60 cancer cell lines at a concentration of 10 µM. Some compounds evaluated showed a significant antiproliferative effect against the tested cell lines. Compound III (Fig. 1) was selected for five-dose evaluation against 60 human tumour cell lines. Compound III exhibited significant selectivity towards the leukemia subpanel, with a selectivity ratio of 5.96. *In vitro* assay results indicated that compound III had substantial antiproliferative activity as a dual inhibitor of EGFR and BRAF^V600E^. Compound III induced apoptosis by elevating caspase 3 and 8 and increasing Bax, while decreasing Bcl-2. Furthermore, molecular docking analyses validated the capability of compound III to function as a dual inhibitor of EGFR and BRAF^V600E^.

### 1.1. Rational design

**Fig. 2:**
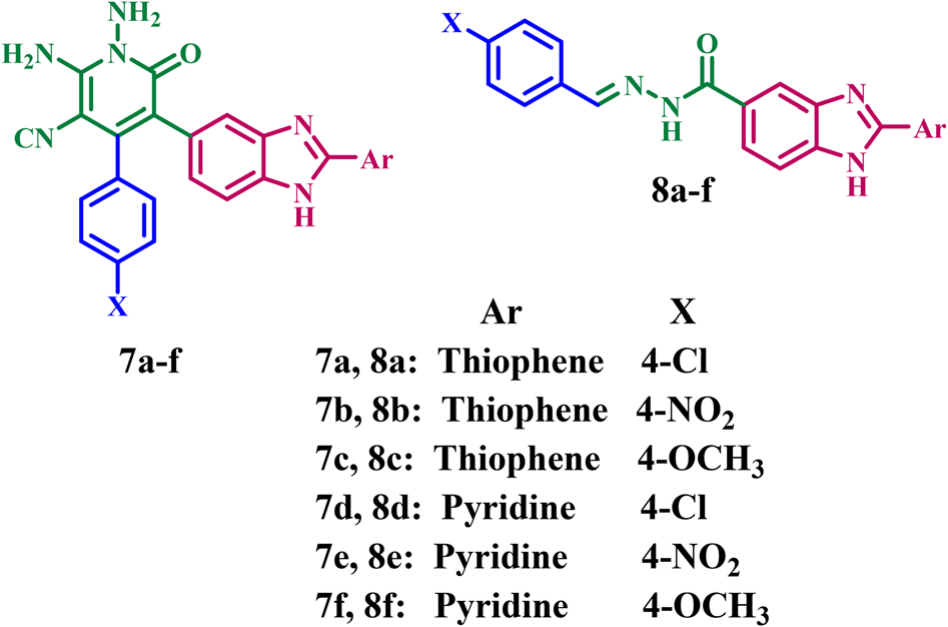
Structures of compounds 7a–f and 8a–f.

Informed by previous data and as part of our ongoing endeavor to develop dual or multi-kinase inhibitors^25–31^ with improved antiproliferative efficacy, we present the design, synthesis, and antiproliferative effects of novel benzimidazole-based derivatives 7a–f and 8a–f (Fig. 2) functioning as dual EGFR/BRAF^V600E^ inhibitors. The novel compounds have distinct pharmacophoric features required for binding to both the EGFR and BRAF^V600E^ active sites. The substituted benzimidazole core (a bioisostere of the adenine core of ATP) serves as a crucial hinge-binding scaffold, enabling significant hydrogen bonding with Met769 in EGFR and Cys532 in BRAF^V600E^, thereby positioning the molecule within the ATP-binding site. The 1,6-dihydropyrimidine-3-carbonitrile in compounds 7a–f, or the acylhydrazone in 8a–f, serves as a linker, placing the terminal phenyl ring deep within the hydrophobic pocket, next to the gatekeeper residues. This is very important for blocking EGFR effectively. The nitrogen atoms in the hydrazone moiety or in the heterocyclic moieties serve as hydrogen-bond acceptors and donors, maintaining BRAF in either the DFG-out or DFG-in conformation, thereby enabling its activity. Furthermore, various substituents (NO_2,_ –OMe, -and Cl) were incorporated to evaluate the impact of differing electronic effects and to optimize hydrophobic interactions within the kinase catalytic domains.

The structures of compounds 7a–f (scaffold A) and 8a–f (scaffold B) were validated by ^1^H NMR, ^13^C NMR, and EIMS spectroscopy. The antiproliferative properties of the new compounds were assessed against different cancer cell lines: breast, colon, and hepatic. The most potent antiproliferative agents were then assessed for their activity against EGFR and BRAF^V600E^. Moreover, the apoptotic potential, encompassing Caspases-3, -8, and -9, BAX, and Bcl-2, was assessed. Finally, docking and ADMET studies were performed against the most effective derivative targeting EGFR and BRAF^V600E^.

## 2. Experimental

### 2.1. Chemistry

General details: see Appendix A (SI).

Compounds 3a–b,^27^4a–b,^26^5a–b,^25^ and 6a–b^32^ were prepared according to reported procedures.

#### 2.1.1. General procedure for synthesis of compounds 7a–f

Equimolar amounts of compounds 5a–b (4 mmol) and the corresponding arylidene malononitriles 6a–c (4 mmol) were dissolved in absolute ethanol (20 mL) with a few drops of piperidine. The reaction mixture was refluxed for 4 hours. The resultant crude solid was filtered while hot, dried, and recrystallized from a chloroform/methanol mixture (8 : 2), yielding pure compounds 7a–f in satisfactory yields.

##### 2.1.1.1. 1,2-Diamino-4-(4-chlorophenyl)-6-oxo-5-(2-(thiophen-2-yl)-1*H*-benzimidazol-5-yl)-1,6-dihydropyridine-3-carbonitrile (7a)

Brownish powder, 79% yield, m.p. 252–254 °C; ^1^H NMR (DMSO-*d*_6_, 400 MHz, *δ* ppm): 4.42 (s, 2H, NH_2_, D_2_O exchangeable), 7.24 (d, 2H, *J* = 8.00 Hz, Ar–H)), 7.51–7.54 (m, 2H, Ar–H), 7.65 (s, 1H, Ar–H), 7.75–7.88 (m, 5H, Ar–H), 8.47 (s, 2H, NH_2_, D_2_O exchangeable), 11.49 (s, 1H, NH benzimidazole, D_2_O exchangeable); ^13^C NMR (DMSO-*d*_6_, 100 MHz, *δ* ppm):100.00, 117.31 (C≡N), 122.86, 127.85, 128.04, 128.95, 129.16, 129.37, 129.45, 130.02, 130.98, 133.68, 133.99, 134.93, 143.57, 146.53, 149.64, 159.23, (aromatic carbons), 161.65 (C–NH_2_), 164.22 (C=O); C_23_H_15_ClN_6_OS (458.92): MS (EI) *m*/*z* calculated for C_23_H_15_ClN_6_OS (458.92), found: 459.12 ([M + H]^+^, 8.17%).

##### 2.1.1.2. 1,2-Diamino-4-(4-nitrophenyl)-6-oxo-5-(2-(thiophen-2-yl)-1*H*-benzimidazol-5-yl)-1,6-dihydropyridine-3-carbonitrile (7b)

Yellow powder, 73% yield, m.p. 232–234 °C; ^1^H NMR (DMSO-*d*_6_, 400 MHz, *δ* ppm): 4.41 (s, 2H, NH_2_, D_2_O exchangeable), 7.26 (s, 2H, Ar–H), 7.67 (s, 2H, Ar–H), 7.76 (d, 2H, *J* = 8.00 Hz, Ar–H), 7.81–7.89 (m, 3H, Ar–H), 8.19 (s, 2H, NH_2_, D_2_O exchangeable), 8.33 (s, 1H, Ar–H), 12.52 (s, 1H, NH benzimidazole, D_2_O exchangeable); ^13^C NMR (DMSO-*d*_6_, 100 MHz, *δ* ppm): 98.56, 107.61, 108.71, 111.83 (C≡N), 122.98, 124.55, 127.48, 128.09, 128.39, 128.96, 130.09, 133.62, 141.35, 145.23, 148.21, 149.69, 153.19, 157.97, (aromatic carbons), 164.36 (C–NH_2_), 166.88 (C=O). MS (EI) *m*/*z* calculated for C_23_H_15_N_7_O_3_S (469.48):, found: 469.10 ([M + H]^+^, 2.77%).

##### 2.1.1.3. 1,2-Diamino-4-(4-methoxyphenyl)-6-oxo-5-(2-(thiophen-2-yl)-1*H*-benzimidazol-5-yl)-1,6-dihydropyridine-3-carbonitrile (7c)

Brown powder, 78% yield, m.p. 256–258 °C; ^1^H NMR (DMSO-*d*_6_, 400 MHz, *δ* ppm): 3.80 (s, 3H, OCH_3_), 6.99–7.02 (m, 4H, Ar–H), 7.24 (s, 1H, Ar–H), 7.67–7.88 (m, 5H, 3Ar–H and NH_2_, D_2_O exchangeable), 8.14 (s, 2H, Ar–H), 8.42 (s, 2H, NH_2_, D_2_O exchangeable), 11.77 (s, 1H, NH benzimidazole, D_2_O exchangeable); ^13^C NMR (DMSO-*d*_6_, 100 MHz, *δ* ppm): 55.81 (OCH_3_), 93.57, 99.98, 105.32, 110.12, 114.87 (C≡N), 119.58, 122.70, 127.58, 128.07, 129.16, 129.98, 130.45, 133.75, 142.01, 147.78, 149.62, 157.23, 161.29 (aromatic carbons), 163.99 (C–NH_2_), 166.42 (C=O). MS (EI) *m*/*z* calculated for C_24_H_18_N_6_O_2_S (454.51), found: 454.09 ([M + H]^+^, 5.32%).

##### 2.1.1.4. 1,2-Diamino-4-(4-chlorophenyl)-6-oxo-5-(2-(pyridin-4-yl)-1*H*-benzimidazol-5-yl)-1,6-dihydropyridine-3-carbonitrile (7d)

Brown powder, 73% yield, m.p. 275–277 °C; ^1^H NMR (DMSO-*d*_6_, 400 MHz, *δ* ppm): 5.00 (s, 2H, NH_2_, D_2_O exchangeable), 7.42 (d, 1H, *J* = 8.00 Hz, Ar–H), 7.74 (d, 2H, *J* = 8.00 Hz, Ar–H), 7.84–7.87 (m, 4H, Ar–H), 8.09 (d, 2H, *J* = 8.00 Hz, Ar–H), 8.23–8.27 (m, 2H, Ar–H), 8.47 (s, 2H, NH_2_, D_2_O exchangeable), 12.06 (s, 1H, NH benzimidazole, D_2_O exchangeable); ^13^C NMR (DMSO-*d*_6_, 100 MHz, *δ* ppm): 100.00, 117.30 (C≡N), 121.08, 123.39, 128.37, 129.18, 129.43, 130.97, 133.93, 134.95, 135.25, 140.16, 141.79, 146.62, 151.10, 151.56, 159.09 (aromatic carbons), 163.21 (C–NH_2_), 164.09 (C=O). MS (EI) *m*/*z* calculated for C_24_H_16_ClN_7_O (453.89), found: 452.37 ([M + H]^+^, 2.82%).

##### 2.1.1.5. 1,2-Diamino-4-(4-nitrophenyl)-6-oxo-5-(2-(pyridin-4-yl)-1*H*-benzimidazol-5-yl)-1,6-dihydropyridine-3-carbonitrile (7e)

Brown powder, 77% yield, m.p. 279–281 °C; ^1^H NMR (DMSO-*d*_6_, 400 MHz, *δ* ppm): 7.75 (d, 1H, *J* = 8.00 Hz, Ar–H), 7.86 (d, 1H, *J* = 8.00 Hz, Ar–H), 7.99 (s, 2H, NH_2_, D_2_O exchangeable), 8.10–8.16 (m, 2H, Ar–H), 8.27–8.29 (m, 4H, Ar–H), 8.57 (s, 2H, NH_2_, D_2_O exchangeable), 8.77 (m, 3H, Ar–H), 12.23 (s, 1H, NH benzimidazole, D_2_O exchangeable); ^13^C NMR (DMSO-*d*_6_, 100 MHz, *δ* ppm): 100.00, 115.35 (C≡N), 116.95, 117.27, 121.07, 121.46, 123.47, 124.54, 128.09, 128.40, 135.25, 137.23, 140.01, 141.32, 145.33, 148.25, 151.10 (aromatic carbons), 151.57 (C–NH_2_), 164.23 (C=O). MS (EI) *m*/*z* calculated for C_24_H_16_N_8_O_3_ (464.45), found: 463.98 ([M − H]^+^, 20.25%).

##### 2.1.1.6. 1,2-Diamino-4-(4-methoxyphenyl)-6-oxo-5-(2-(pyridin-4-yl)-1*H*-benzimidazol-5-yl)-1,6-dihydropyridine-3-carbonitrile (7f)

Yellow powder, 85% yield, m.p. 270–272 °C, ^1^H NMR (DMSO-*d*_6_, 400 MHz, *δ* ppm): 3.89 (s, 3H, OCH_3_), 7.10 (s, 3H, Ar–H and NH_2_, D_2_O exchangeable), 7.78 (m, 2H, Ar–H),7.92 (d, 2H, *J* = 8.00 Hz, Ar–H), 8.32 (m, 3H, Ar–H), 8.50 (m, 3H, Ar–H), 8.88 (s, 2H, NH_2_, D_2_O exchangeable), 11.95 (s, 1H, NH benzimidazole, D_2_O exchangeable); ^13^C NMR (DMSO-*d*_6_, 100 MHz, *δ* ppm): 55.76 (OCH_3_), 86.96, 100.02, 108.46, 114.85 (C≡N), 118.12, 121.07, 123.38, 127.55, 128.76, 129.19, 130.64, 132.52, 137.27, 144.28, 148.01, 151.10, 151.40 (aromatic carbons), 161.32 (C–NH_2_), 163.93 (C=O). MS (EI) *m*/*z* calculated for C_25_H_19_N_7_O_2_ (449.47), found: 449.88 ([M + H]^+^, 7.14%).

#### 2.1.2. General procedure for synthesis of compounds 8a–f

Equimolar quantities of compounds 5a–b (2 mmol) and different substituted benzaldehydes were refluxed in 25 mL of absolute ethanol for 6 hours using glacial acetic acid as a catalyst. The resulting crude material was filtered and recrystallized from ethanol, yielding pure 8a–f.

##### 2.1.2.1. *N*′-(4-Chlorobenzylidene)-2-(thiophen-2-yl)-1*H*-benzimidazole-5-carbohydrazide 8a

Buff powder, 79% yield, m.p. 203–205 °C; ^1^H NMR (DMSO-*d*_6_, 400 MHz, *δ* ppm): 7.25 (d, 1H, *J* = 8.00 Hz, Ar–H), 7.51 (d, 1H, *J* = 8.00 Hz, Ar–H), 7.77–7.88 (m, 3H, Ar–H), 8.05 (s, 1H, Ar–H), 8.23 (s, 1H, Ar–H), 8.47 (m, 4H, CH=NH), 11.95 (s, 1H, NH, D_2_O exchangeable), 13.23 (s, 1H, NH benzimidazole, D_2_O exchangeable); ^13^C NMR (DMSO-*d*_6_, 100 MHz, *δ* ppm):111.64, 118.75, 122.23, 123.38, 128.07, 129.15, 129.45, 129.99, 133.69, 134.01, 134.92, 137.88, 143.84, 146.52, 149.40, 149.99, 164.25 (C=O). MS (EI) *m*/*z* calculated for C_19_H_13_ClN_4_OS (380.45), found: 382.06 ([M + H]^+^, 4.56%).

##### 2.1.2.2. *N*′-(4-Nitrobenzylidene)-2-(thiophen-2-yl)-1*H*-benzimidazole-5-carbohydrazide 8b

Yellow powder, 63% yield, m.p. 234–236 °C; ^1^H NMR (DMSO-*d*_6_, 400 MHz, *δ* ppm): 7.25 143.75 7.61–7.71 (m, 2H, Ar–H), 7.78–7.81 (m, 2H, Ar–H), 8.06 (d, 2H, *J* = 8.00 Hz, Ar–H), 8.24–8.28 (m, 3H, Ar–H), 8.57 (s, 1H, CH=NH), 12.19 (s, 1H, NH, D_2_O exchangeable), 13.26 (s, 1H, NH benzimidazole, D_2_O exchangeable); ^13^C NMR (DMSO-*d*_6_, 100 MHz, *δ* ppm): 118.72, 122.39, 123.36, 124.55, 127.00, 128.06, 128.92, 130.05, 133.65, 135.00, 137.96, 141.39, 143.75, 145.21, 148.25, 149.51, 164.34 (C=O). MS (EI) *m*/*z* calculated for C_19_H_13_N_5_O_3_S (391.41), found: 391.83 ([M + H]^+^, 5.09%).

##### 2.1.2.3. *N*′-(4-Methoxybenzylidene)-2-(thiophen-2-yl)-1*H*-benzimidazole-5-carbohydrazide 8c

Buff powder, 77% yield, m.p. 291–293 °C; ^1^HNMR (DMSO-*d*_6_, 400 MHz, *δ* ppm): 3.80 (s, 3H, OCH_3_), 7.01 (d, 1H, *J* = 8.00 Hz, Ar–H),7.25 (s, 1H, Ar–H), 7.59–7.69 (m, 4H, Ar–H), 7.77–7.89 (m, 2H, Ar–H), 8.04–8.22 (m, 2H, Ar–H), 8.42 (s, 1H, CH=NH), 11.76 (s, 1H, NH, D_2_O exchangeable), 13.22 (s, 1H, NH benzimidazole, D_2_O exchangeable); ^13^C NMR (DMSO-*d*_6_, 100 MHz, *δ* ppm): 55.77 (OCH_3_),111.54, 114.86, 118.65, 122.22, 123.26, 127.61, 127.97, 128.91, 129.15, 129.98, 133.74, 135.03, 137.78, 143.77, 147.84, 161.30, 164.05 (C=O). MS (EI) *m*/*z* calculated for C_20_H_16_N_4_O_2_S (376.43), found: 377.35 ([M + H]^+^, 5.56%).

##### 2.1.2.4. *N*′-(4-Chlorobenzylidene)-2-(pyridin-4-yl)-1*H*-benzimidazole-5-carbohydrazide 8d

Yellowish powder, 76% yield, m.p. 225–227 °C; ^1^HNMR (DMSO-*d*_6_, 400 MHz, *δ* ppm): 7.51 (s, 2H, Ar–H), 7.76–7.87 (m, 2H, Ar–H), 8.11–8.19 (m, 3H, Ar–H), 8.47 (s, 2H, Ar–H), 8.78 (s, 2H, Ar–H, CH=NH), 11.99 (s, 1H, NH, D_2_O exchangeable), 13.62 (s, 1H, NH benzimidazole, D_2_O exchangeable);^13^C NMR (DMSO-*d*_6_, 100 MHz, *δ* ppm): 99.97, 112.57, 121.10, 123.37, 129.20, 129.38, 133.79, 135.01, 137.18, 146.81, 151.02, 151.41, 164.25, 172.72 (C=O). MS (EI) *m*/*z* calculated for C_20_H_14_ClN_5_O (375.82), Found: 376.11 ([M + H]^+^, 3.11%) and 378.07 (M^+^+2, 2.56%).

##### 2.1.2.5. *N*′-(4-Nitrobenzylidene)-2-(pyridin-4-yl)-1*H*-benzimidazole-5-carbohydrazide 8e

Brown powder, 82% yield, m.p. 217–219 °C; ^1^H NMR (DMSO-*d*_6_, 400 MHz, *δ* ppm): 7.72 (m, 3H, Ar–H), 7.90–8.00 (m, 5H, Ar–H), 8.29 (d, 1H, *J* = 8.00 Hz, Ar–H), 8.41–8.58 (m, 2H, Ar–H), 8.79 (s, 1H, CH=NH), 11.95 (s, 1H, NH, D_2_O exchangeable), 13.59 (s, 1H, benzimidazole NH, D_2_O exchangeable); ^13^C NMR (DMSO-*d*_6_, 100 MHz, *δ* ppm): 117.53, 119.77, 121.07, 124.53, 128.39, 135.53, 137.20, 141.30, 142.30, 143.49, 145.33, 148.23, 150.48, 151.08, 164.20, 172.57(C=O). MS (EI) *m*/*z* calculated for C_20_H_14_N_6_O_3_ (386.37), found: 386.62 ([M + H]^+^, 3.24%).

##### 2.1.2.6. *N*′-(4-Methoxybenzylidene)-2-(pyridin-4-yl)-1*H*-benzimidazole-5-carbohydrazide 8f

Brown powder, 74% yield, m.p. 220–222 °C; ^1^H NMR (DMSO-*d*_6_, 400 MHz, *δ* ppm): 3.76 (s, 3H, CH_3_), 6.98 (d, 1H, *J* = 8.40 Hz, Ar–H), 7.65–7.87 (m, 4H, Ar–H), 8.11 (d, 2H, *J* = 8.40 Hz, Ar–H), 8.29 (s, 1H, Ar–H), 8.44 (s, 1H, CH=NH), 8.76 (d, 4H, *J* = 8.00 Hz, 3 Ar–H, NH, D_2_O exchangeable), 12.10 (s, 1H, NH benzimidazole, D_2_O exchangeable); ^13^C NMR (DMSO-*d*_6_, 100 MHz, *δ* ppm): 55.78 (OCH_3_), 114.83, 115.51, 116.52, 121.09, 123.28, 127.54, 128.61, 129.19, 137.40, 140.17, 141.82, 145.14, 147.97, 151.07, 161.30, 163.97 (C=O). MS (EI) *m*/*z* calculated for C_21_H_17_N_5_O_2_ (371.40), found: 371.08 ([M + H]^+^, 8.73%).

### 2.2. Biology

#### 2.2.1. Cell viability assay

The effect of 7a–f and 8a–f on cell viability was examined using the MCF-10A normal cell line. The MTT assay was employed to evaluate the cell viability of the compounds under investigation after a 4 day incubation with MCF-10A cells.^29,33^ For additional experimental information, refer to Appendix A.

#### 2.2.2. Antiproliferative assay

Novel compounds 7a–f and 8a–f were evaluated for their antiproliferative abilities against three human cancer cell lines: MCF-7 (mammary gland breast cancer), HepG2 (hepatocellular carcinoma), and HCT-116 (colorectal carcinoma), as well as against the normal human diploid cell line (WI-38). To evaluate activity, the MTT assay was performed.^34,35^ The IC_50_ values for the new compounds were determined through the dose-response experiments. The results were derived from at least two separate investigations, each with three replicates per concentration. For additional details, consult Appendix A.

#### 2.2.3. BRAF^V600E^ inhibitory assay

Vemurafenib was employed as the reference drug to evaluate the *in vitro* efficacy of compounds 7a, 7b, 7e, 8c, 8d, and 8f against BRAF^V600E^ using the B-Raf(V600E) Kinase Assay Kit (Catalog # 48688; San Diego, CA 92121).^36^ The IC_50_ values were presented as the results. Experimental details are provided in Appendix A.

#### 2.2.4. EGFR inhibitory assay

The efficacy of compounds 7a, 7b, 7e, 8c, 8d, and 8f in targeting EGFR-TK was evaluated using the EGFR Kinase Assay Kit Catalog # 40321(San Diego, CA 92121).^37^ The IC_50_ values for each compound were determined and compared with those of erlotinib, which served as the reference standard. For additional experimental information, check Appendix A.

#### 2.2.5. Apoptotic markers assays

Compounds 8d and 8f were evaluated as caspase-3, -8, and -9 activators and as down-regulators of the anti-apoptotic protein Bcl-2 in the HCT-116 colorectal cancer cell line, using staurosporine as a ref. 38. Additional information is provided in Appendix A.

## 3. Results and discussion

### 3.1. Chemistry

**Scheme 1:**
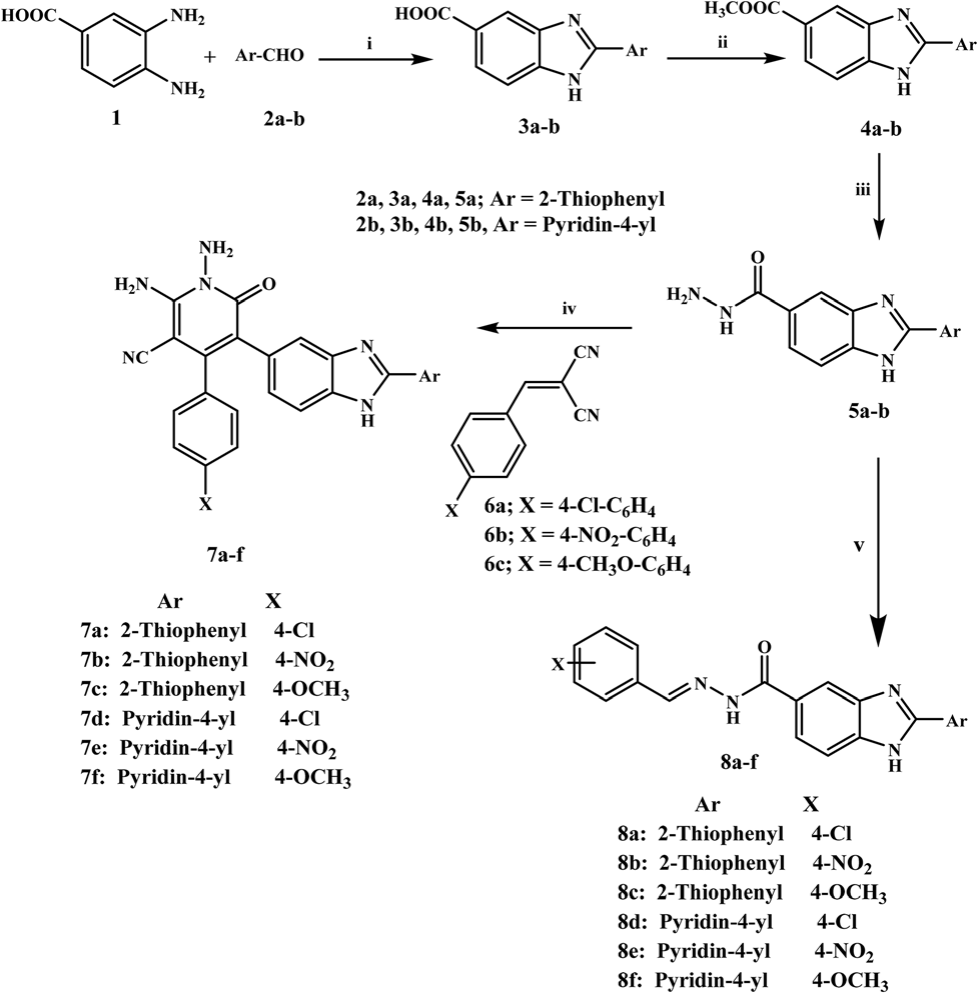
Synthesis of compounds 7a–f and 8a–f.

The synthesis protocol for the final compounds 7a–f and 8a–f is outlined in Scheme 1. The benzimidazole-5-carboxylic acid derivatives 3a–b were synthesised with an 83% yield through the reaction of 3,4-diaminobenzoic acid 1 and thiophene-2-carbaldehyde 2a or pyridine-4-carbaldehyde 2b under reflux for 6 hours in the presence of sodium metabisulfite and dimethyl formamide (DMF).^27^ The Fischer esterification of carboxylic acids 3a–b was carried out by heating the compounds in methanol with a few drops of concentrated H_2_SO_4_ under reflux for 17 hours, affording esters 4a–b with a productivity of 77%.^26^ Hydrazide derivatives 5a–b were synthesised by refluxing the respective esters 4a–b with 99% hydrazine monohydrate in ethanol for 12 hours, affording 63%25 yield. Furthermore, substituted benzylidene malononitriles 6a–c were synthesised by dissolving a mixture of malononitrile and the corresponding substituted benzaldehyde in ethanol, with a few drops of piperidine as a catalyst. The mixture was heated for 15 minutes and thereafter stirred at room temperature for 1 to 2 hours.^32^

The structure of representative compound 4a was validated by ^1^H NMR spectroscopy. The ester group was recognized by a distinctive three-proton singlet at *δ* 3.84 ppm, indicative of the methoxy protons (–COOCH_3_). The benzimidazole core was indicated by a broad, downfield singlet at *δ* 13.32 ppm (N1–H), characteristic of this structure in DMSO-*d*_6_ owing to hydrogen bonding. Moreover, the aromatic region integrated for six protons, precisely aligning with the anticipated signals from both the benzimidazole structure and the thiophene substituent.

#### 3.1.1 Reagent and reaction conditions

(i) DMF, Na_2_S_2_O_8,_ reflux 6 h; 83% (ii) Conc. H_2_SO_4_, methanol, reflux 17 h; 77% (iii) NH_2_NH_2_, H_2_O, ethanol, reflux 12 h; 63% (iv) arylidenemalononitrile, ethanol, piperidine, reflux 4 h, (V) aldehydes, ethanol, a few drops of CH_3_COOH, reflux 6 h.

The final target compounds 7a–f were synthesised in high yields by heating hydrazide derivatives 5a–b with the corresponding substituted arylidene malononitriles 6a–c in absolute ethanol, catalyzed by a few drops of piperidine, followed by refluxing for 4 hours. The crude products were recrystallized from a methanol/chloroform mixture to obtain pure 7a–f.

The structures of 7a–f were validated using different spectroscopic techniques. The ^1^H NMR spectra exhibited a broad signal at *δ* 11.49–12.52 ppm, indicative of the benzimidazole NH proton, whereas the ^13^C NMR spectra displayed signals at *δ* 111.83–117.31 ppm (C≡N carbon) and *δ* 151.57–164.36 ppm and 163.93–166.88 ppm for C–NH_2_ and C=O, respectively. The ^1^H NMR spectrum of 7d, as a representative example, exhibited an exchangeable signal at *δ* 5.47 ppm, corresponding to the NH_2_ group. An NH signal of the benzimidazole structure was detected at *δ*: 12.06 ppm. The ^13^C NMR spectrum of 7d exhibited a signal at *δ*: 117.30 ppm corresponding to the C≡N carbon, and at *δ*: 163.21 and 164.09 corresponding to C–NH_2_ and C=O, respectively.

Moreover, the condensation reaction of compounds 5a–b with various aromatic aldehydes in ethanol, using a few drops of glacial acetic acid as a catalyst, was carried out under reflux for 6 hours, yielding the corresponding hydrazones 8a–f. The ^1^H NMR spectra of compounds 8a–f exhibited singlet signals at *δ* = 8.42–8.79 ppm, corresponding to CH=NH, at 11.19–12.19 ppm, associated with –NH moieties that are exchangeable with D_2_O, and at 12.10–13.59 ppm, linked to –NH benzimidazole, which are also exchangeable with D_2_O. Furthermore, the ^13^C NMR spectra of 8a–f displayed a characteristic peak at *δ* 163.97–172.72 corresponding to (C=O). The ^1^H NMR spectrum of 8c exhibited a singlet signal at *δ* 3.80, corresponding to the OCH_3_ group, two signals at *δ* 8.42 and *δ* 11.76 ppm, attributable to the azomethine proton CH=N and hydrazone NH, respectively, and a signal at *δ* 13.22 associated with the NH of benzimidazole. The ^13^C NMR spectrum of 8c exhibited novel signals at *δ* 55.77, 161.30, and 164.05 ppm, corresponding to OCH_3_, hydrazone C=N, and C=O, respectively.

Additionally, we conduct COSY-2D (500 MHz spectrometer) which is crucial for resolving the multiplets of aromatic protons (7.76–7.87 and 8.11–8.19, compound 8d), which encompass overlapping signals from three distinct aromatic rings. The COSY-2D NMR spectrum offers definitive proof of the structural connectivity of *N*′-(4-chlorobenzylidene)-2-(pyridin-4-yl)-1*H*-benzimidazole-5-carbohydrazide (8d) by delineating three separate, isolated spin systems. The lack of cross-peaks between these systems verifies that the aromatic rings have been separated by quaternary carbons and the carbohydrazide linker, as anticipated by the IUPAC structure.

#### 3.1.2 The pyridine and azomethine region

In the downfield region, the COSY spectrum identifies a clear correlation between the signals at *δ* 8.78 and *δ* 8.11. While the ^1^H NMR signal at 8.78 ppm represents a 2H integration (accounting for both the pyridine H_α_ and the singlet azomethine CH=N proton), the 2D correlation specifically links the pyridine H_α_ to its vicinal H_β_ partner at 8.11 ppm. The azomethine proton, despite overlapping in the ^1^H dimension, shows no cross-peaks, confirming its isolation from any vicinal protons within three bonds.

#### 3.1.3 The benzimidazole core

The 1,2,4-trisubstituted benzimidazole ring is characterized by a specific coupling network between H6 and H7. A distinct cross-peak is observed between the multiplet at *δ* 8.19 (H6) and the signal at *δ* 7.51 (H7), typical of *ortho*-coupling in the benzimidazole scaffold. Notably, the signal representing H4 (appearing near *δ* 8.15) remains as a “diagonal-only” peak or shows only very weak *meta*-coupling, which is consistent with its position between the quaternary C5 (carbohydrazide attachment) and the bridgehead carbon.

#### 3.1.4 The 4-chlorobenzylidene system

**Fig. 3:**
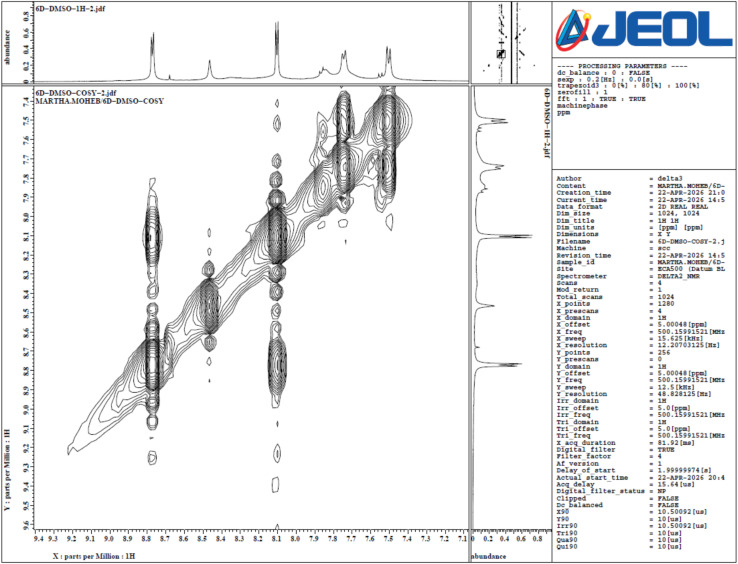
2D-COSY of compound 8d.

The *para*-substituted chlorophenyl ring displays a classic AA‘XX’ symmetric coupling pattern. This is evidenced by the strong, symmetric cross-peaks between the protons *ortho* to the imine linker at *δ* 7.76–7.87 (H2′, 6′) and those *ortho* to the chlorine atom at *δ* 7.51 (H3′, 5′). This “square” correlation confirms the integrity of the 4-chlorobenzylidene moiety. Finally, the highly downfield signals at *δ* 11.99 and *δ* 13.62 show no COSY correlations, verifying their assignment as the exchangeable NH protons of the hydrazide and benzimidazole groups, respectively, Fig. 3 and Table 1.

**Table 1:** Summary of correlations in 2D-COSY of compound 8d

Proton(s)	Chemical shift (*δ*)	Coupled to (*via* COSY)	Group
H_α_	8.78	8.11 (H_β_)	Pyridine
H_2′, 6′_	7.82	7.51 ((H_3′, 5′_))	4-Cl-phenyl
H_6_	8.19	7.51 (H_7_)	Benzimidazole
CH=N	8.78	None	Linker

### 3.2. Biology

#### 3.2.1. Cell viability assay

Using the MCF-10A (human mammary gland epithelial) normal cell line, the impact of new compounds 7a–f and 8a–f on cell viability was investigated. The cell viability of the compounds under investigation was assessed after a 4 day incubation with MCF-10A cells using the MTT assay.^29,33^Table 2 indicates that none of the compounds under investigation exhibited cytotoxic effects on normal cells. At a dosage of 50 µM, all compounds maintained cell viability at or above 89%.

**Table 2:** Cell viability% and antiproliferative activity of compounds 7a–f and 8a–f

Comp.	Cell viability%	*In vitro* cytotoxicity IC_50_ (µM)			
		HepG2	HCT-116	MCF-7	Mean IC_50_
7a	91	33.46 ± 2.1	21.52 ± 1.5	15.95 ± 1.2	23.64
7b	93	23.07 ± 1.6	30.19 ± 1.9	19.25 ± 1.4	24.17
7c	90	83.74 ± 4.2	>100	74.21 ± 3.7	85.93
7d	89	42.79 ± 2.5	48.73 ± 2.8	29.58 ± 2.0	40.36
7e	90	38.32 ± 2.3	36.20 ± 2.2	27.68 ± 1.8	34.06
7f	92	59.41 ± 3.3	78.81 ± 3.8	45.71 ± 2.5	61.31
8a	90	71.38 ± 3.8	67.78 ± 3.6	63.51 ± 3.3	67.55
8b	91	52.93 ± 3.0	86.51 ± 4.4	39.85 ± 2.3	59.76
8c	89	18.07 ± 1.4	16.84 ± 1.3	12.47 ± 1.0	15.88
8d	90	5.95 ± 0.4	3.98 ± 0.2	6.34 ± 0.4	5.42
8e	92	47.31 ± 2.8	56.17 ± 3.2	32.64 ± 2.1	45.37
8f	91	7.72 ± 0.6	9.82 ± 0.7	8.12 ± 0.7	8.55
Doxorubicin	—	4.50 ± 0.2	5.23 ± 0.3	4.17 ± 0.2	4.63

#### 3.2.2. Antiproliferative assay

The antiproliferative efficacy of novel compounds 7a–f (scaffold A) and 8a–f (scaffold B) was assessed against three human cancer cell lines: MCF-7 (mammary gland breast cancer), HepG2 (hepatocellular carcinoma), and HCT-116 (colorectal carcinoma). The MTT assay was employed to assess activity.^36^ The cell line was acquired from ATCC (American Type Culture Collection) *via* the Holding Company for Biological Products and Vaccines (VACSERA) in Cairo, Egypt. Doxorubicin functioned as the control in this study. Table 2 presents the median inhibitory concentration (IC_50_) and the average IC_50_ for the three cancer cell lines.

The antiproliferative efficacy of scaffold A (pyridine-3-carbonitrile-based) compounds 7a–f was predominantly mild to moderate, with mean IC_50_ values spanning from 23 to 85 µM against the three cancer cell lines evaluated. Conversely, the standard Doxorubicin demonstrated a mean IC_50_ value of 4.63 µM. Compounds 7a–f exhibit a potency that is at least 4.6-fold inferior to that of Doxorubicin against the evaluated cancer cell lines, with compounds 7a, 7b, and 7e being the most efficient variants among scaffold A (7a–f) compounds. Compounds 7a (Ar = thiophene, X = 4-Cl), 7b (Ar = thiophene, X = 4-NO_2_), and 7e (Ar = pyridin-4-yl, X = 4-NO_2_) demonstrated mean IC_50_ values of 23.64, 24.17, and 34.06 µM, respectively, indicating potencies that are 4.6-, 4.8-, and 6.8-fold inferior to that of Doxorubicin. Compound 7c (Ar = thiophene, X = 4-OMe) had the lowest potency, both among scaffold A derivatives and among all newly synthesised compounds. It exhibited a mean IC_50_ value of 85.93 µM, indicating a potency that is 17-fold inferior to that of Doxorubicin. These findings indicated that the presence of a pyridine-3-carbonitrile moiety at the fifth position of the benzimidazole moiety is detrimental to antiproliferative activity.

Compounds 8a–f derived from scaffold B (carbohydrazide-based) exhibited notable antiproliferative activity, with mean IC_50_ values ranging from 5.42 to 67.55 µM, compared to Doxorubicin's IC_50_ value of 4.63 µM. Compounds 8c, 8d, and 8f exhibited the highest potency among the derivatives, with mean IC_50_ values of 15.88, 5.42, and 8.55 µM, respectively. Of the newly synthesised derivatives 7a–f and 8a–f, compound 8d (Ar = pyridin-4-yl, X = 4-Cl, scaffold B) exhibited the highest potency, with a mean IC_50_ value of 5.42 µM. It exhibited comparable potency to Doxorubicin (mean IC_50_ = 4.63 µM). Compound 8d surpassed the reference drug Doxorubicin in efficacy against the HCT-116 cancer cell line, with an IC_50_ value of 3.98 ± 0.2 µM. Doxorubicin exhibited an IC_50_ value of 5.23 ± 0.3 µM for the same cell line. The results indicated that compound 8d was 1.3 times more effective than Doxorubicin against the HCT-116 colorectal cancer cell line; however, compound 8d showed reduced efficacy compared to Doxorubicin against the other cancer cell lines.

Compound 8f (Ar = pyridin-4-yl, X = OMe, scaffold B), structurally analogous to compound 8d but featuring a methoxy group at the *para* position of the *N*-phenyl carbohydrazide moiety, exhibited the second-highest antiproliferative activity with a mean IC_50_ of 8.55 µM, demonstrating 1.6-fold reduced potency compared to compound 8d. This suggests that a chlorine atom at the *para* position of the *N*-phenyl carbohydrazide moiety is more beneficial to antiproliferative activity than a methoxy group. Compound 8f exhibited 2.5-fold reduced potency compared to 8d against the colorectal cancer cell line HCT-116.

Compound 8e (Ar = pyridin-4-yl, X = NO_2_, scaffold B), structurally similar to 8d but containing a nitro group at the *para* position of the *N*-phenyl carbohydrazide moiety, displayed weak antiproliferative activity with a mean IC_50_ of 45.37 µM, indicating an 8-fold decrease in potency over that of compound 8d. This indicates that when pyridine-4-yl serves as the aromatic substituent at the second position of the benzimidazole nucleus, the chlorine atom at the *para* position of the *N*-phenyl carbohydrazide moiety enhances antiproliferative activity more effectively than a methoxy group. In contrast, the nitro group is detrimental to activity.

Furthermore, the substitution pattern at the second position of the benzimidazole moiety (Ar) is essential for the antiproliferative activity of compounds 8a–f. Compound 8a (Ar = thiophene, X = 4-Cl, scaffold B) possesses an identical structure to compound 8d, differing only by the presence of a thiophene group at the second position of the benzimidazole moiety. The average IC_50_ of 8a was 67.55 µM, indicating that 8a was at least 12 times less effective than compound 8d (average IC_50_ = 5.42 µM). The data indicate that the pyridine-4-yl group at the second position of the benzimidazole nucleus is essential for antiproliferative action. The identical principle can be employed when analyzing the activity of 8b with 8e and 8c*versus*8f, as illustrated in Table 1.

#### 3.2.3. Cytotoxicity assay against normal cell line

Consequently, it was imperative to assess the selectivity of the target compounds for cancer cells *versus* normal cells by evaluating the safety profiles of the most potent compounds, 8d and 8f, in the normal human diploid cell line (WI-38) using the MTT assay.^39^ The IC_50_ values of compounds 8d and 8f were 76.12 ± 3.8 and 84.02 ± 4.3 µM, respectively. Table 3 shows that the evaluated compounds exhibited a favorable safety margin relative to normal cells.

**Table 3:** The selectivity index and IC_50_ values of 8d and 8f against the WI-38 normal cell line

Compound	Cytotoxicity (WI-38) IC_50_ (µM)	Selectivity index (SI)		
		HCT-116	HepG2	MCF-7
8d	76.12 ± 3.8	>19	>12	12
8f	84.02 ± 4.3	>8	>10	>10

#### 3.2.4. BRAF^V600E^ inhibitory assay

The *in vitro* efficacy of compounds 7a, 7b, 7e, 8c, 8d, and 8f, the most potent antiproliferative agents, against BRAF^V600E^ was assessed, using vemurafenib as the reference drug.^36^ The results were displayed as IC_50_ values in Table 4. The enzyme assays demonstrated that the assessed compounds exhibited significant inhibition of BRAF^V600E^, with IC_50_ values ranging from 45 to 253 nM, compared with vemurafenib, which had an IC_50_ of 41 nM. Compound 8d (R = pyridin-4-yl, R_1_ = 4-Cl), recognized as the most effective antiproliferative agent, also had the greatest efficacy as a BRAF^V600E^ inhibitor, with an IC_50_ value of 45.50 ± 1.80 nM, showing comparable potency to the reference drug vemurafenib (IC_50_ value = 41.40 ± 1.13 nM). Compounds 7e (R = pyridin-4-yl, R_1_ = 4-NO_2_) and 8f (R = pyridin-4-yl, R_1_ = 4-OMe) had significant BRAF^V600E^ inhibitory activity, with IC_50_ values of 78 nM and 57 nM, respectively. They achieved second- and third-rank activity, exhibiting 1.7- and 1.3-fold reductions in potency as BRAF^V600E^ inhibitors relative to 8d, respectively.

**Table 4:** IC_50_ values of 7a, 7b, 7e, 8c, 8d, and 8f against BRAF^V600E^ and EGFR

Compound	BRAF^V600E^ (IC_50_ nM)	EGFR (IC_50_ nM)
7a	198.10 ± 11.94	56.77 ± 3.41
7b	113.25 ± 4.25	53.26 ± 1.87
7e	78.10 ± 2.08	39.86 ± 2.93
8c	253.50 ± 15.32	22.90 ± 1.73
8d	45.50 ± 1.80	7.17 ± 0.55
8f	57.70 ± 3.98	11.48 ± 0.69
Vemurafenib	41.38 ± 1.13	—
Erlotinib	—	5.40 ± 0.13

Compounds 7a, 7b, and 8c demonstrated mild to moderate anti-BRAF activity, with IC_50_ values of 198.10, 113.25, and 253.50 nM, respectively, and were at least 2.5-fold less potent than compound 8d and vemurafenib. These observations indicate that compounds 8d and 8f are strong antiproliferative agents that may function as mutant-BRAF inhibitors.

#### 3.2.5. EGFR inhibitory assay

Compounds 7a, 7b, 7e, 8c, 8d, and 8f were subsequently assessed for their ability to target EGFR-TK.^37^ The IC_50_ values for each compound were determined and compared with those of erlotinib, serving as the reference standard. Table 4 lists the IC_50_ values.

The results of this *in vitro* assay were consistent with those of the BRAF^V600E^ inhibitory assay, in which compounds 8d and 8f, the most potent BRAF^V600E^ inhibitors, were also the most effective EGFR inhibitors. Compounds 8d and 8f demonstrated significant EGFR inhibition with IC_50_ values of 7.17 and 11.48 nM, respectively, exhibiting 1.3- and 2.1-fold reductions in potency relative to erlotinib, which had an IC_50_ of 5.40 nM. Compounds 7a, 7b, 7e, and 8c demonstrated substantial EGFR inhibitory activity, with IC_50_ values ranging from 22.90 to 56.77 nM, indicating at least a fourfold reduction in potency relative to erlotinib.

The results show that compounds 8d and 8f are potent dual-target inhibitors, with significant activity against both BRAF^V600E^ and EGFR. Compound 8d is the more promising of the two, with IC_50_ values of 45.50 nM for BRAF^V600E^ and 7.17 nM for EGFR, which are similar to the established reference drugs, vemurafenib and erlotinib, respectively. Although vemurafenib has marginally higher efficacy against the BRAF mutation and erlotinib has superior potency against EGFR, 8d's ability to maintain single-digit or low-double-digit nanomolar potency across both kinases suggests its potential as a highly effective treatment for cancers that concurrently exploit these pathways, thereby avoiding single-agent therapies.

#### 3.2.6. Apoptotic induction assays

Despite the therapeutic efficacy of targeted BRAF inhibitors, treatment resistance is common due to compensatory activation of EGFR. Combining BRAF^V600E^ and EGFR inhibitors is an effective approach to prevent the compensatory pathway from functioning.^31^ The ultimate efficacy of targeted therapy is contingent upon its capacity to convert kinase inhibition into apoptotic induction.^40^ Apoptotic cell death is regulated by the Bcl-2 protein family. Depending on the equilibrium between pro-apoptotic BAX and anti-apoptotic Bcl-2, the outer membrane of the mitochondria is either more or less permeable. This triggers the caspase cascade, which results in apoptosis.^41,42^ The objective of this investigation is to assess the pro-apoptotic potential of compounds 8d and 8f by analyzing their effects on critical apoptotic markers (Caspases, BAX, and Bcl-2), thereby elucidating their mechanism of action in inducing cytotoxic stress.

##### 3.2.6.1. Caspase assay

The activation of the respective initiator caspases in the dual BRAF^V600E^/EGFR inhibitors 8d and 8f was investigated to distinguish between extrinsic and intrinsic apoptotic pathways and to elucidate the specific mechanism of cell death induced by these inhibitors. We evaluated Caspase-8, which is recruited to initiate the extrinsic pathway following death receptor ligation at the plasma membrane, and Caspase-9, the primary initiator of the intrinsic (mitochondrial) pathway, which is activated by intracellular stress and cytochrome c release.^43^ Our objective is to ascertain whether our compounds induce a distinct apoptotic pathway or a convergent “crosstalk” between mitochondrial and receptor-mediated signaling, leading to the activation of executioner Caspase-3, by analyzing both pathways. The impact of compounds 8d and 8f on caspase-3 was assessed in HCT-116 (colorectal) cancer cell line and compared to staurosporine as a reference medication,^38^Table 5.

**Table 5:** Caspases-3, -8, and -9 levels of compounds 8d and 8f

Compd no.	Caspase-3		Caspase-8		Caspase-9	
	Conc (pg ml^−1^)	Fold change	Conc (ng ml^−1^)	Fold change	Conc (ng ml^−1^)	Fold change
8d	493 ± 4	8.2	1.05 ± 0.08	10	26.50 ± 1	26
8f	445 ± 4	7.4	0.90 ± 0.06	9	23.80 ± 1	24
Staurosporine	420 ± 3	7.0	1.90 ± 0.10	19	20.00 ± 1	20
Control	60	1.0	0.10	1	1.00	1

The assessment of Caspase-3 levels revealed that compounds 8d and 8f are potent activators of the apoptotic execution phase. Both compounds surpassed staurosporine, a prominent broad-spectrum kinase inhibitor regarded as the reference standard for inducing apoptosis. Compound 8d demonstrated the most significant pro-apoptotic activity, with a value of 493 ± 4 pg ml^−1^, which is 8.2 times higher than that of untreated control cells. Potent activation indicates that the concurrent inhibition of BRAF^V600E^ and EGFR by 8d generates a synergistic stress signal that cellular survival mechanisms cannot evade. Compound 8f exhibited superior performance relative to staurosporine, with a 7.4-fold increase in activity compared to a 7.0-fold enhancement. Both compounds effectively convert kinase inhibition into a distinct “death signal”. The significant elevation of Caspase-3 levels confirms that the observed antiproliferative effects of 8d and 8f are due to programmed cell death rather than nonspecific necrosis.

Additionally, the effects of 8d and 8f on the levels of Caspases 8 (extrinsic route) and 9 (intrinsic pathway) were examined. The dual BRAF^V600E^/EGFR inhibitors 8d and 8f substantially enhance the activity of both initiator caspases, indicating that these compounds facilitate apoptosis *via* a convergent mechanism that activates both extrinsic and intrinsic pathways. The most important result is that Caspase-9 is quite active. Compound 8d caused a 26-fold increase (26.50 ± 1 ng ml^−1^), while compound 8f caused a 24-fold increase (23.80 ± 1 ng ml^−1^). In this sense, both compounds outperformed staurosporine by a factor of 20. This indicates that the primary mechanism of action of these dual inhibitors is the induction of significant cellular stress, which likely makes the outer mitochondrial membrane permeable.

##### 3.2.6.2. BAX and Bcl-2 levels assays

**Fig. 4:**
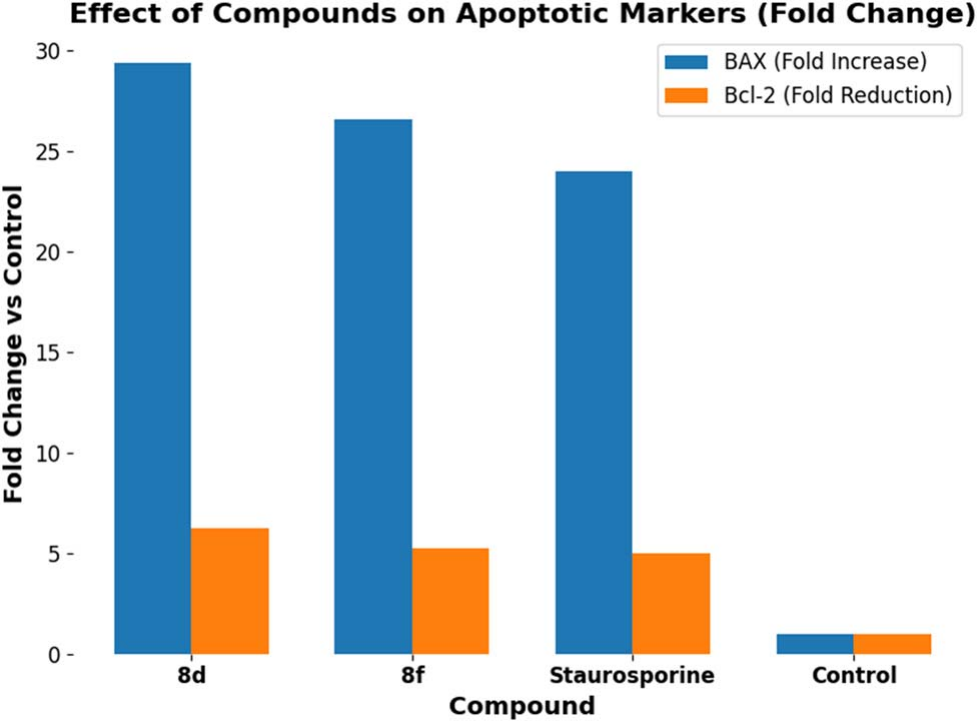
Effect of compounds 8d and 8f on apoptotic markers BAX and Bcl-2.

Compounds 8d and 8f were analyzed for their effects on pro-apoptotic BAX and anti-apoptotic Bcl-2 levels in the MCF-7 human breast cancer cell line, using staurosporine as a ref. 38, as shown in Table 6 and Fig. 4.

**Table 6:** Assays of BAX and Bcl-2 for compounds 8d and 8f

Compd no.	BAX		Bcl-2	
	Conc (pg ml^−1^)	Fold change	Conc (ng ml^−1^)	Fold reduction
8d	265 ± 1	29.40	0.80	6.25
8f	240 ± 1	26.60	0.95	5.25
Staurosporine	220 ± 1	24.00	1.00	5.00
Control	9.00	s1	5.00	1

The pro-apoptotic effects of compounds 8d and 8f were further corroborated by their modulation of Bcl-2 family proteins, which regulate the intrinsic apoptotic pathway. Both compounds altered the cellular balance by raising BAX levels and lowering Bcl-2 levels, thereby increasing the likelihood of apoptosis. BAX levels were raised 29.4-fold with compound 8d and 26.6-fold with compound 8f (Fig. 4). Both derivatives were much more effective than staurosporine (20-fold), indicating that inhibiting both BRAF^V600E^ and EGFR is a highly effective way to initiate the mitochondrial stress response. At the same time, 8d and 8f successfully “inhibited” the survival signal sent out by Bcl-2. Compared to the control (5.00 ng ml^−1^), compound 8d reduced Bcl-2 levels by 6.25-fold (0.80 ng ml^−1^), indicating that it was more effective than staurosporine. The simultaneous elevation of BAX and reduction of Bcl-2 yields a substantial pro-apoptotic ratio. This imbalance results in the loss of mitochondrial membrane integrity, leading to a 26-fold increase in Caspase-9 levels.

In conclusion, apoptotic profiling of 8d and 8f shows an enormous shift in activity from hindering both BRAF^V600E^ and EGFR to starting programmed cell death. These compounds increase the permeability of the mitochondrial membrane by raising the BAX/Bcl-2 ratio. This leads to strong activation of Caspase-9, followed by Caspase-3. This means that 8d and 8f are quite effective at causing apoptosis, even more than staurosporine.

### 3.3. Docking study

#### 3.3.1. Docking study into the EGFR active site

Using Autodock Vina software,^44^ a molecular docking study was conducted to investigate the binding interactions of the most effective compound, 8 d, with EGFR, using a proven crystallographic structure of EGFR complexed with erlotinib (PDB ID: 1M17)^45^ as a reference model. The protein structures were preprocessed using energy minimization using the OPLS-AA force field. Standard docking preparation methods, such as water removal, protonation of titratable residues, and assignment of appropriate bond orders, were applied to improve docking accuracy.

**Fig. 5:**
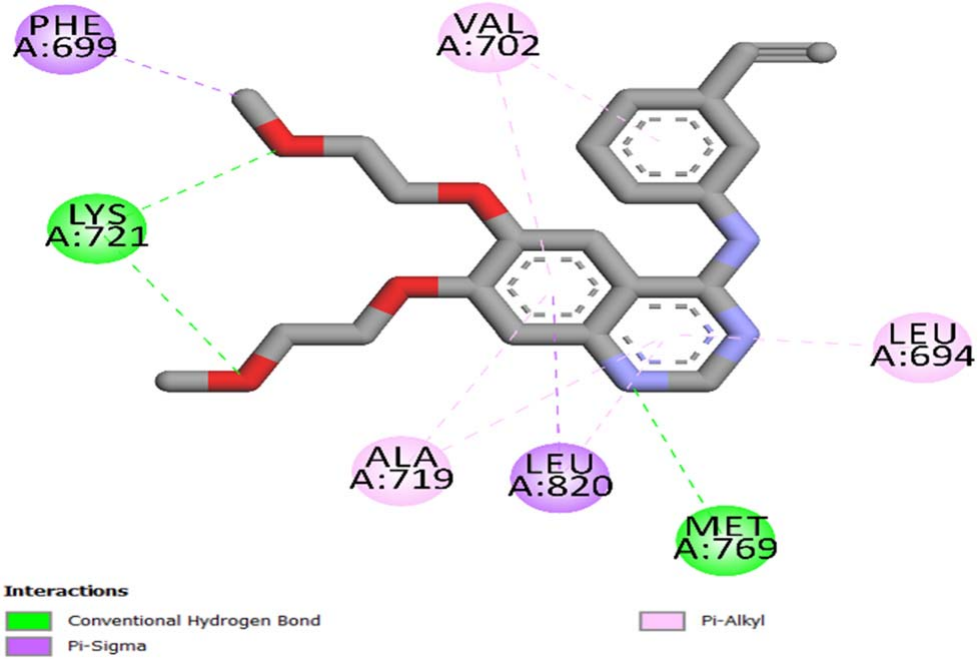
2D representation of re-docked erlotinib into the EGFR active site (PDB ID: 1M17).

To authenticate the docking process, the co-crystallized ligands (erlotinib for EGFR and vemurafenib for BRAF^V600E^) were re-docked into their corresponding binding sites. The redocking of erlotinib into EGFR (RMSD = 1.12 Å) yielded an S score of −7.40 kcal mol^−1^, precisely reproducing the native binding conformation with a critical hydrogen bond between the pyrimidine nitrogen and Met769, thereby validating the docking methodology. Erlotinib formed an additional hydrogen bond with Lys721 *via* the ethoxy group, and a network of hydrophobic interactions involving Leu694, Phe699, Val702, Ala719, and Leu820 (Fig. 5).

**Fig. 6:**
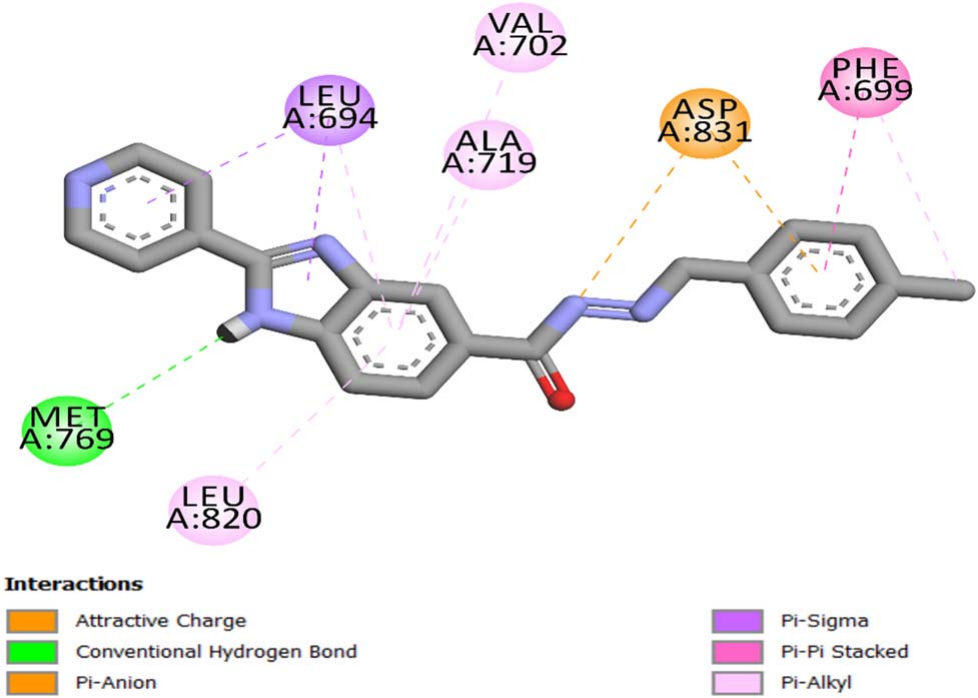
2D representation of 8d into the EGFR active site (PDB ID: 1M17).

Compound 8d comprises three primary components: the benzimidazole ring, the carbohydrazide moiety, and the *p*-chlorophenyl moiety, which collectively form a distinctive pharmacophoric structure. Each feature significantly enhances the binding affinity in the ATP-binding pocket of EGFR. The benzimidazole ring serves as the principal anchoring motif, forming a vital hydrogen bond with the hinge residue Met769, an interaction essential for effective inhibition of ATP-competitive kinases. The ring's flat shape facilitates precise alignment inside the confined hinge area. The neighboring carbohydrazide group enhances planarity by stabilizing the entire compound's binding through attractive charge interactions with Asp 831. Furthermore, 8d's *p*-chlorophenyl ring interacts with Asp831 *via* a pi-anion interaction, thereby improving binding stability and directional specificity. Finally, Fig. 6 shows that the entire molecule forms a network of hydrophobic interactions with Leu694, Phe699, Ala719, Val702, and Leu820. The pronounced binding affinity (−8.70 kcal mol^−1^) and RMSD of 1.32 observed in the docking studies, along with the substantial *in vitro* inhibitory activity of 8 d, are attributable to these synergistic effects.

#### 3.3.2. Docking study into BRAF^V600E^ active site

**Fig. 7:**
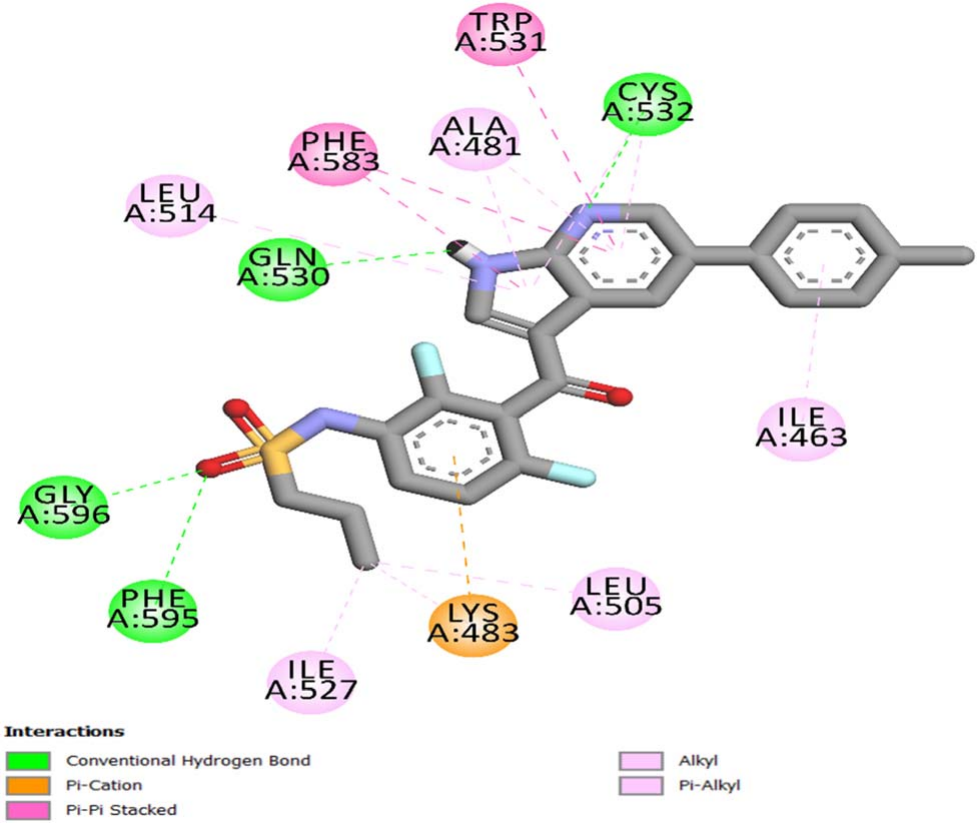
2D representation of vemurafenib into the BRAF^V600E^ active site (PDB ID: 3OG7).

The co-crystallized ligand, vemurafenib, was re-docked into its binding site (PDB ID: 3OG7)^46^ to assess the docking process for BRAF^V600E^. The redocking method yielded a docking score of −11 kcal mol^−1^ and an RMSD of 1.48 Å relative to the experimental conformation, indicating the docking approach's reliability and excellent agreement. The stable binding orientation of vemurafenib preserved the necessary hydrogen bonds with Cys532 and Gly596, which are critical interactions that stabilize inhibitors in the ATP-binding area, as illustrated in Fig. 7. In addition to a network of hydrophobic contacts between Trp531, Phe583, and Ala481, vemurafenib formed two hydrogen bonds with Gln530 and Phe595.

**Fig. 8:**
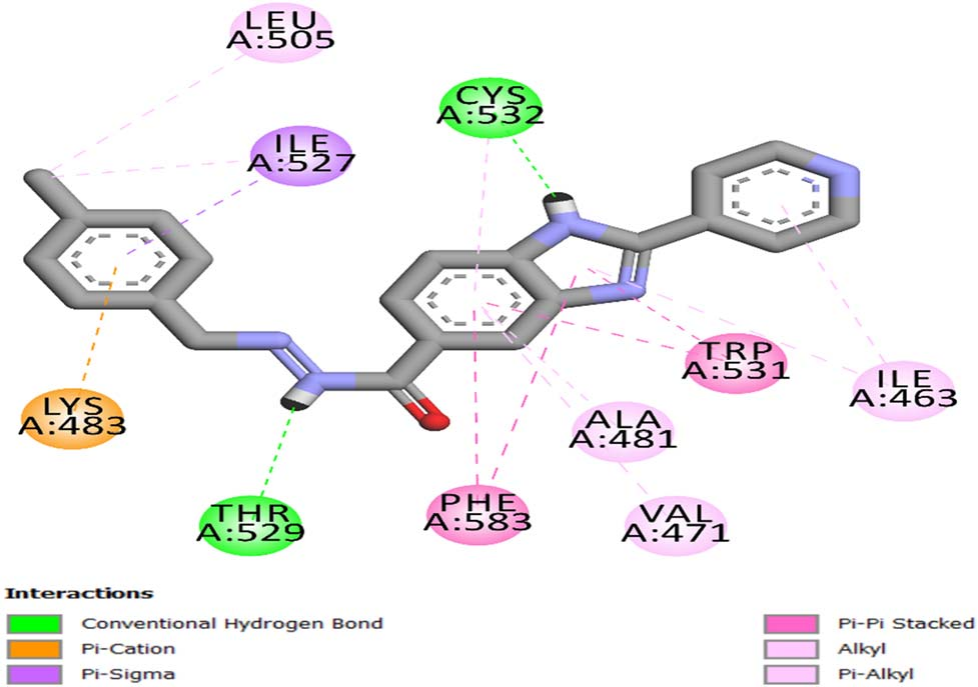
2D representation of 8d into the BRAF^V600E^ active site (PDB ID: 3OG7).

The docking of 8d into the ATP-binding site of BRAF^V600E^ exhibited a well-aligned and energetically advantageous binding configuration, indicated by a docking score of −9.40 kcal mol^−1^ and an RMSD of 1.39 Å from the reference pose. The ligand is accurately positioned at the active site, with the benzimidazole ring forming a hydrogen bond with the backbone of Cys532 and participating in hydrophobic interactions with Trp531, Ala481, Val471, and Phe583, as depicted in Fig. 8. Additionally, the carbohydrazide moiety forms a hydrogen bond with Thr529. The scaffold's location in the hinge region is further maintained by the pi-cation interaction of the *p*-chlorophenyl moiety with Lys483 and the pi-sigma interaction with Ile527.

### 3.4. ADMET analysis

The ADMET data analysis, using ADMETlab 3.0,^47^ for compound 8d showed that it was highly lipophilic and rigid. It has a strong aromatic character, with 21 of 27 heavy elements, and a low molecular weight of 375.81 g mol^−1^. The structure of 8d is completely flat; its *C*_sp^3^_ fraction is 0.00. This may enhance binding within kinase compartments *via* π-stacking; however, it could compromise solubility in water and compromise metabolic stability. A TPSA of 83.03 Å^2^, which has 2 hydrogen bond donors and 4 acceptors, meets the best circumstances for oral bioavailability and probable penetration of the blood–brain barrier. This is a very important trait for treating BRAF^V600E^ brain metastases. The absence of sp^3^-hybridized carbons suggests a “flat” shape that could limit its selectivity within the kinase family, even though it is quite efficient.

The lipophilicity data indicate that 8d possesses moderate to high lipophilicity, with a Consensus log *P*_o/w_ of 3.43, positioning it within the optimal range for drug-like compounds. The consensus outcome reveals a balanced profile that enhances membrane permeability and robust hydrophobic interactions inside the deep ATP-binding pockets of EGFR and BRAF. Individual predictors vary from the conservative ilog *P* (2.12) to the highly lipophilic SILICOS-IT (4.62). However, due to the Fraction *C*_sp^3^_ being 0.00, a log *P* exceeding 3.0 may impede the molecule's solubility in water, since it lacks the requisite “out-of-plane” saturation to disrupt crystal lattice stacking. The values remain well below the Lipinski threshold (log *P* < 5), indicating that the compound achieves an effective balance between lipid binding and the body's requirement for water-soluble compounds.

The solubility data indicate a key concern with 8 d: although its lipophilicity is ideal for target binding, its water solubility is merely good to poor, likely due to its rigid, entirely aromatic structure (*F*_sp^3^_ = 0.00), which hinders dissolution.

The ESOL (−4.70) and Ali (−5.05) models signify moderate solubility, the SILICOS-IT model (−8.22) classifies 8d as “insoluble. In a therapeutic context, a solubility of 10^−5^ to 10^−6^ mol L^−1^ often indicates that the molecule's dissolution rate may limit its absorption. This may indicate that more advanced formulation tactics, such as salt formation, micronization, or lipid-based delivery, are required to ensure the compound's bioavailability. However, 8d was expected to be promptly absorbed by the gastrointestinal tract despite its poor solubility. This is most likely the result of its moderate lipophilicity (log *P* 3.43) and the absence of *P*-gp efflux, which ensures its persistence in the systemic circulation after dissolution.

A significant clinical issue is its suppression of all cytochrome P450 enzymes (1A2, 2C19, 2C9, and 2D6); this extensive inhibitory profile renders drug-drug interactions (DDIs) and potential toxicity in multi-drug regimens prevalent in cancer highly probable. The TPSA (83.03 Å^2^) is typically low enough to penetrate the brain; however, the model indicates it cannot cross the blood–brain barrier. This may be due to its rigid, fully aromatic structure or the molecular configuration, which complicates its entry into the central nervous system.

8d possesses an exceptional drug-likeness profile, exhibiting no violations across all primary filtration criteria—Lipinski, Ghose, Veber, Egan, and Muegge. This “clean” hit demonstrates that the compound's size (375.81 g mol^−1^) and polarity are well balanced for pharmaceutical applications, despite the absence of saturated carbon atoms (*F*_sp^3^_ = 0.00). A bioavailability score of 0.55 indicates a substantial probability (about 55%) of accomplishing a minimum of 10% oral bioavailability in rats. This supports its application as an orally processed dual EGFR/BRAF^V600E^ inhibitor.

Finally, 8d has great structural integrity and no PAINS alerts. This suggests that its dual inhibitory effect is probably due to specific binding rather than general interference with the assay. The Brenk filter shows only one warning (imine_1), indicating that the nitrogen-carbon double bond may be unstable or reactive. The synthetic accessibility score of 2.76 means that 8d is easy to synthesize. It is thought to be “drug-like,” however, it doesn't meet the stricter lead likeness standards because its molecular weight (375.81 g mol^−1^) and XLOGP3 (3.62) are both higher than the sensitive limits of 350 and 3.5, respectively. This implies that 8d has already been “optimized” and is nearing its final therapeutic form. There is limited potential to make further structural modifications without displacing 8d from the optimal drug-like area.

## 4. Conclusion

A new series of benzimidazole-derived compounds that act as dual inhibitors of EGFR and BRAF^V600E^, with potential apoptotic and antiproliferative effects, has been identified. Across all *in vitro* investigations, compounds 8c, 8d, and 8f exhibited the highest potency. Compound 8d is the most promising candidate, as its IC_50_ values (45.50 nM for BRAF^V600E^ and 7.17 nM for EGFR) are nearly equivalent to those of vemurafenib (41.38 nM) and erlotinib (5.40 nM). The therapeutic significance of 8d's ability to provide high-affinity inhibition through both routes is noteworthy. This dual-action profile is a strategic chemotherapy strategy designed to prevent feedback activation of EGFR. Furthermore, the intrinsic (mitochondrial) pathway is the primary mechanism by which compounds 8d and 8f significantly induce apoptosis in the examined cell lines. Compared to the extrinsic receptor pathway, both compounds exhibit a higher selectivity for mitochondrial-mediated cell death, as they surpass the reference staurosporine in activating the executioner Caspase-3. Compound 8d is a highly promising dual inhibitor that mimics a pharmaceutical agent. It adheres to Lipinski's rule of five, albeit exhibiting borderline solubility and not penetrating the blood–brain barrier. Despite its elevated GI absorption and clear PAINS profile favoring oral development, the pan-CYP inhibition and Brenk imine warning suggest that subsequent optimization should focus on minimizing metabolic instability and improving its therapeutic index.

## Conflicts of interest

The authors declare no competing interests.

## Supplementary Material

RA-016-D6RA02117D-s001

## Data Availability

The authors declare that the data supporting the findings of this study are available within the supplementary information (SI). Supplementary information is available. See DOI: https://doi.org/10.1039/d6ra02117d.
