# Supplementary material for: Benzimidazole derivatives as dual EGFR and BRAFV600E inhibitors with pro-apoptotic antiproliferative potential
Source: RSC Adv. 2026 May 14;16(28):25216–28. doi: 10.1039/d6ra02117d (PMC13182522; doi:10.1039/d6ra02117d)
Supplement: RA-016-D6RA02117D-s001 [file RA-016-D6RA02117D-s001.pdf]

Supplementary Information (SI) for RSC Advances.
This journal is © The Royal Society of Chemistry 2026

Supplementary Data

Benzimidazole Derivatives as Dual EGFR and BRAF^{V600E} Inhibitors with Pro-Apoptotic Antiproliferative Potential

Lamya H. Al-Wahaibi[1], Mohamed Samir[2], Stefan Bräse[3]*, Bahaa G. M. Youssif*[4], Martha M. Morcoss[5]

[1]Department of Chemistry, College of Sciences, Princess Nourah bint Abdulrahman University, Riyadh 11671, Saudi Arabia; [2]Department of Pharmaceutical Organic Chemistry, Faculty of Pharmacy, Al-Azhar University, 71524 Assiut, Egypt; [3]Institute of Biological and Chemical Systems, IBCS-FMS, Karlsruhe Institute of Technology, 76131 Karlsruhe, Germany; [4]Department of Pharmaceutical Organic Chemistry, Faculty of Pharmacy, Assiut University, Assiut-71526, Egypt; [5]Department of Pharmaceutical Chemistry, Faculty of Pharmacy, Nahda University, 62513 Beni-Suef, Egypt.

To whom correspondence should be addressed:

Bahaa G. M. Youssif, Ph.D. Pharmaceutical Organic Chemistry Department, Faculty of Pharmacy, Assiut University, Assiut 71526, Egypt.

Tel.: (002)-01044353895

E-mail address: bgyoussif2@gmail.com

Stefan Bräse

Institute of Biological and Chemical Systems, IBCS-FMS, Karlsruhe Institute of Technology, 76131 Karlsruhe, Germany. E-mail: braese@kit.edu

Contents

❒ **Copies of ^{1}H NMR, ^{13}C NMR and Mass spectra of all compounds**

S1:^{1}H NMR spectrum of compound 3a (400 MHz, DMSO-d_6)

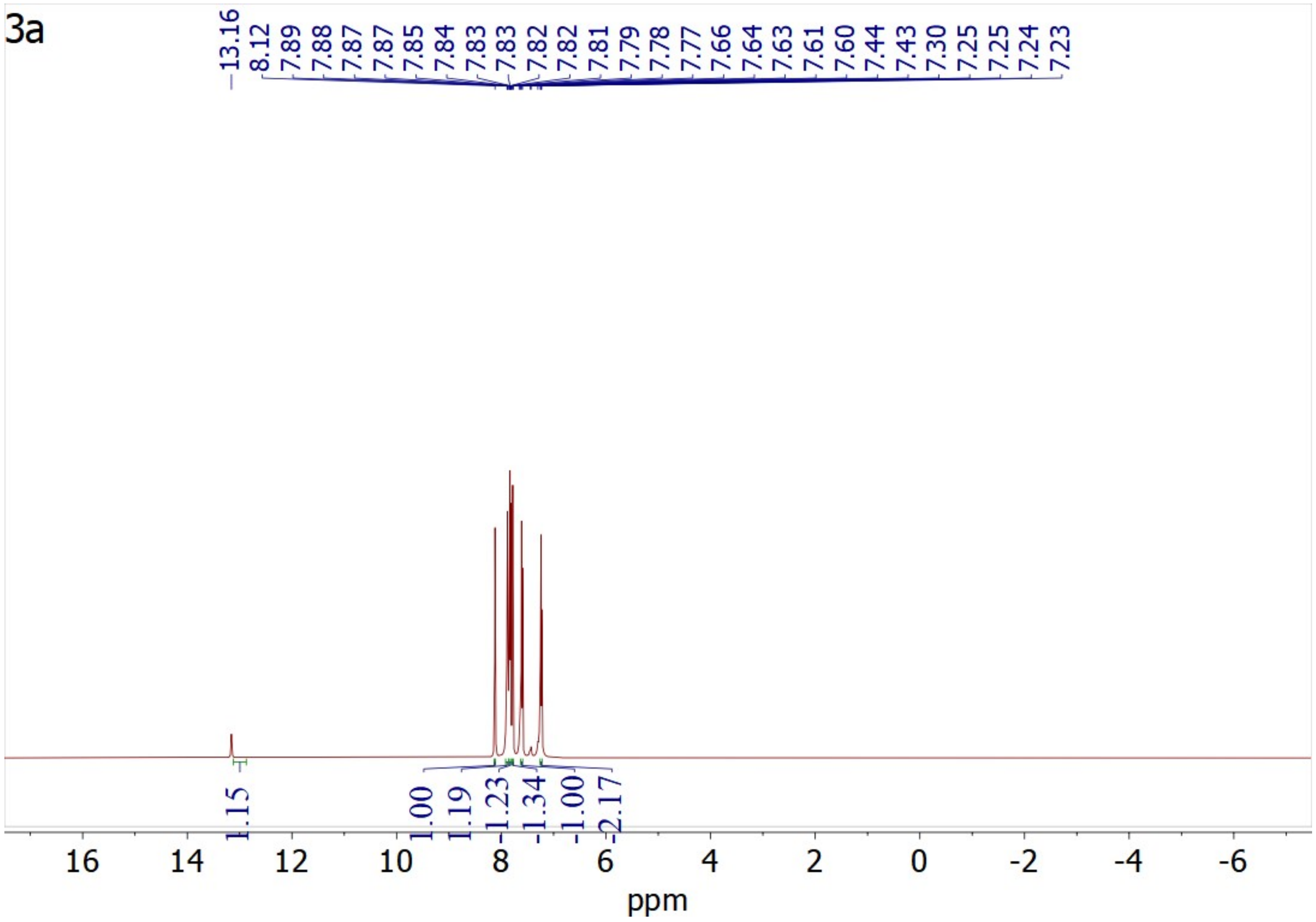

S2: ^{1}H NMR spectrum of compound 4a (400 MHz, DMSO-d_6)

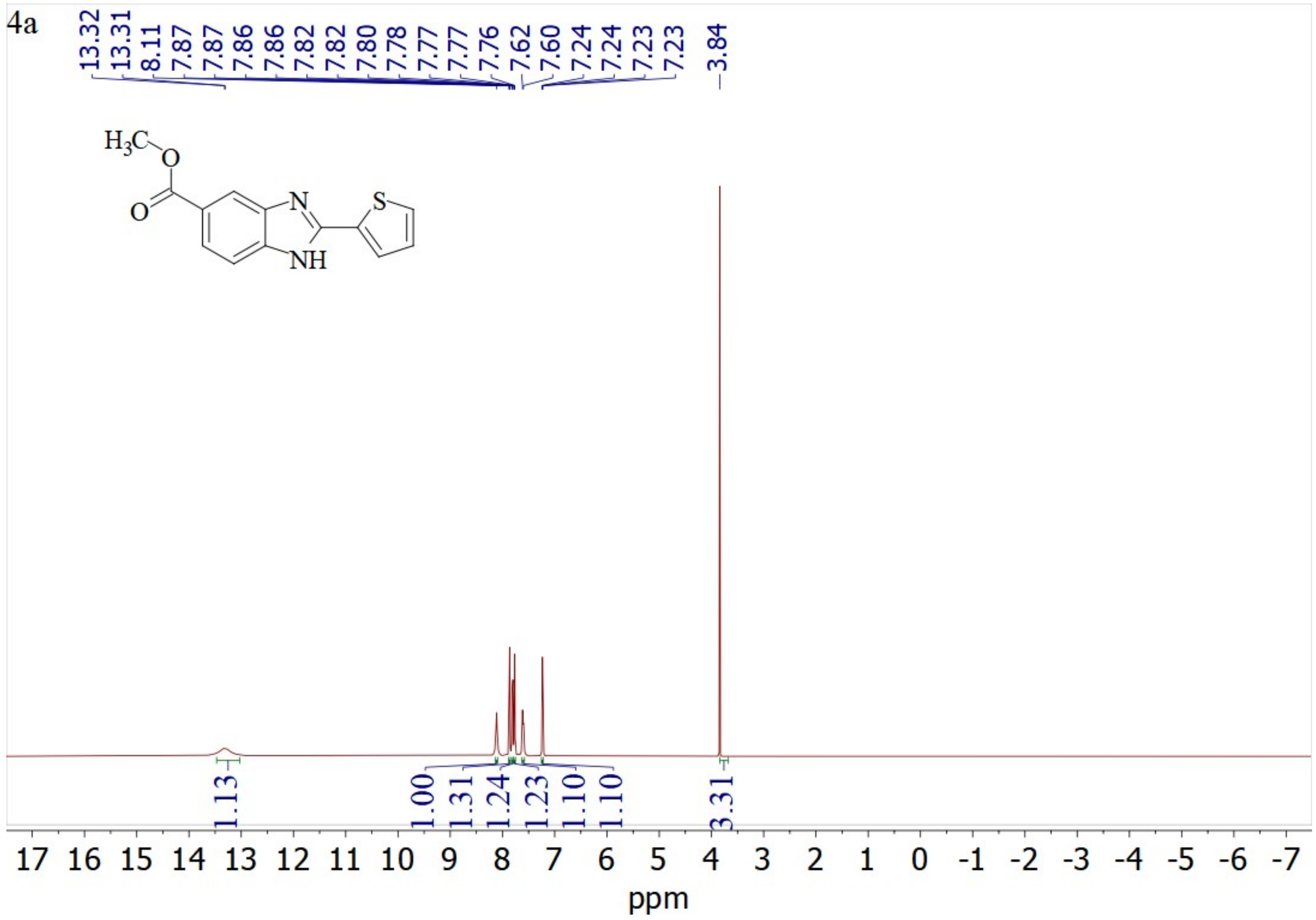

S3: ^{1}H NMR spectrum of compound 5a (400 MHz, DMSO-d_6)

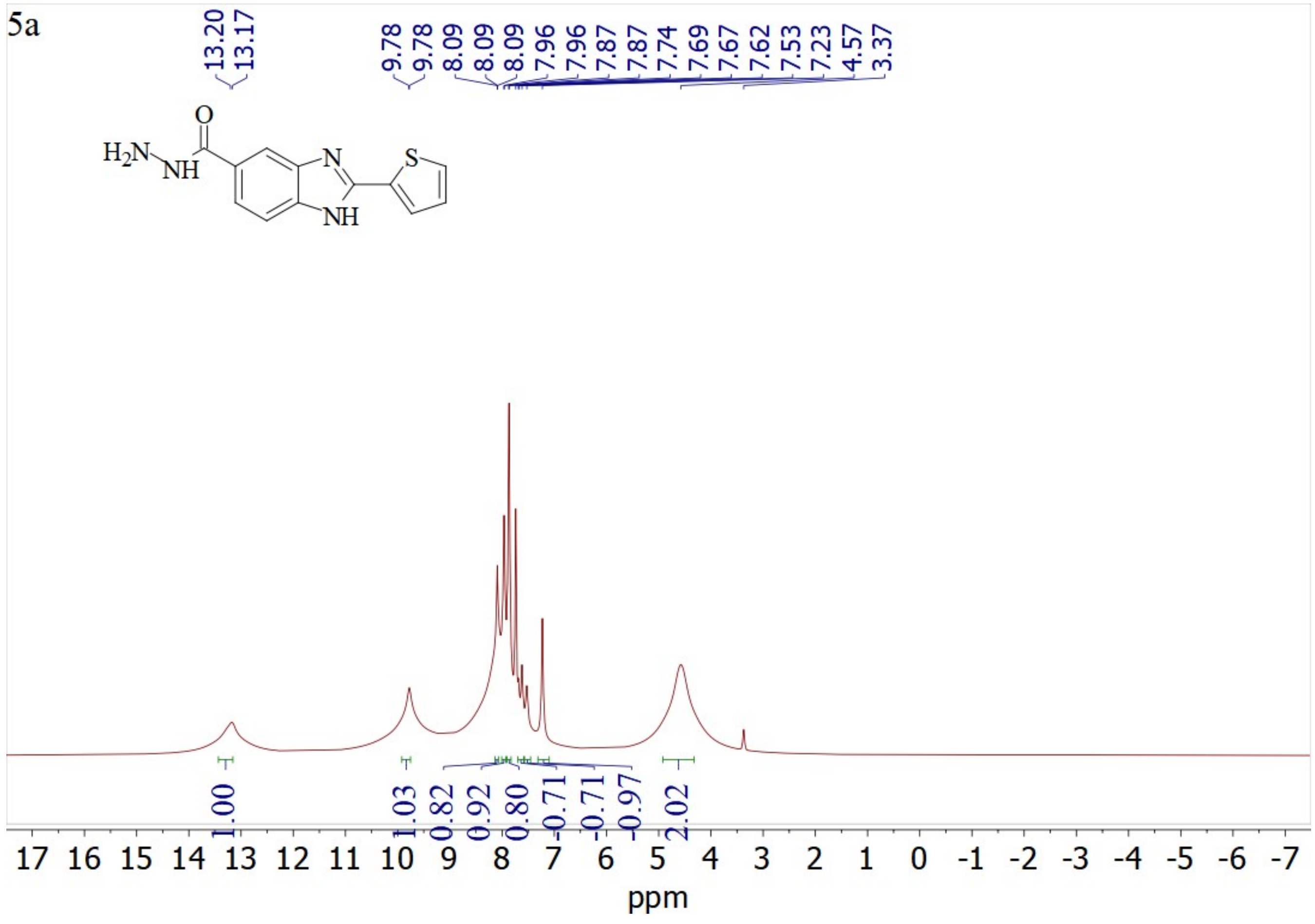

S4: ^{1}H NMR spectrum of compound 7a (400 MHz, DMSO-d_6)

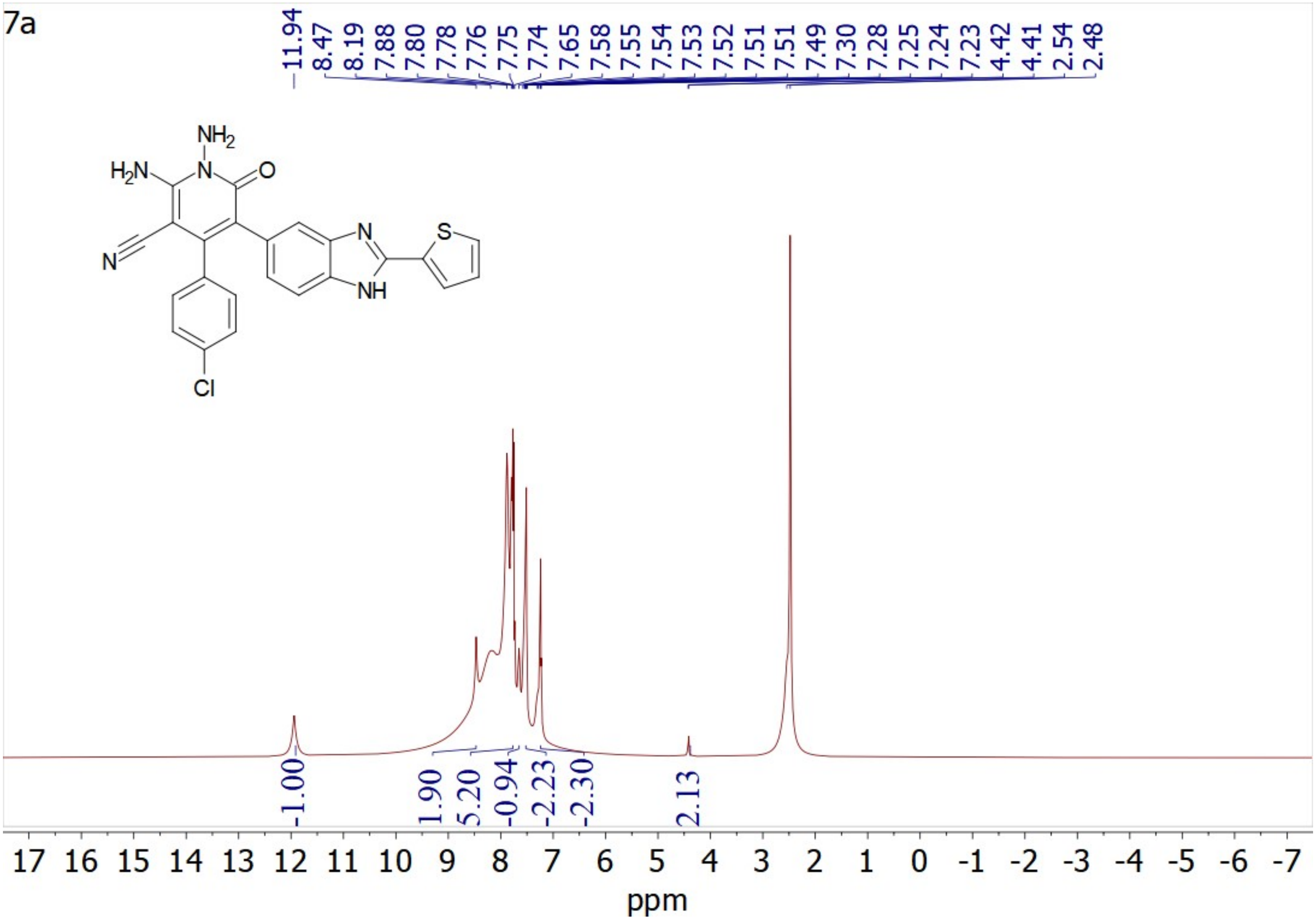

S5: ^{13}C NMR spectrum of compound 7a (100 MHz, DMSO-d_6)

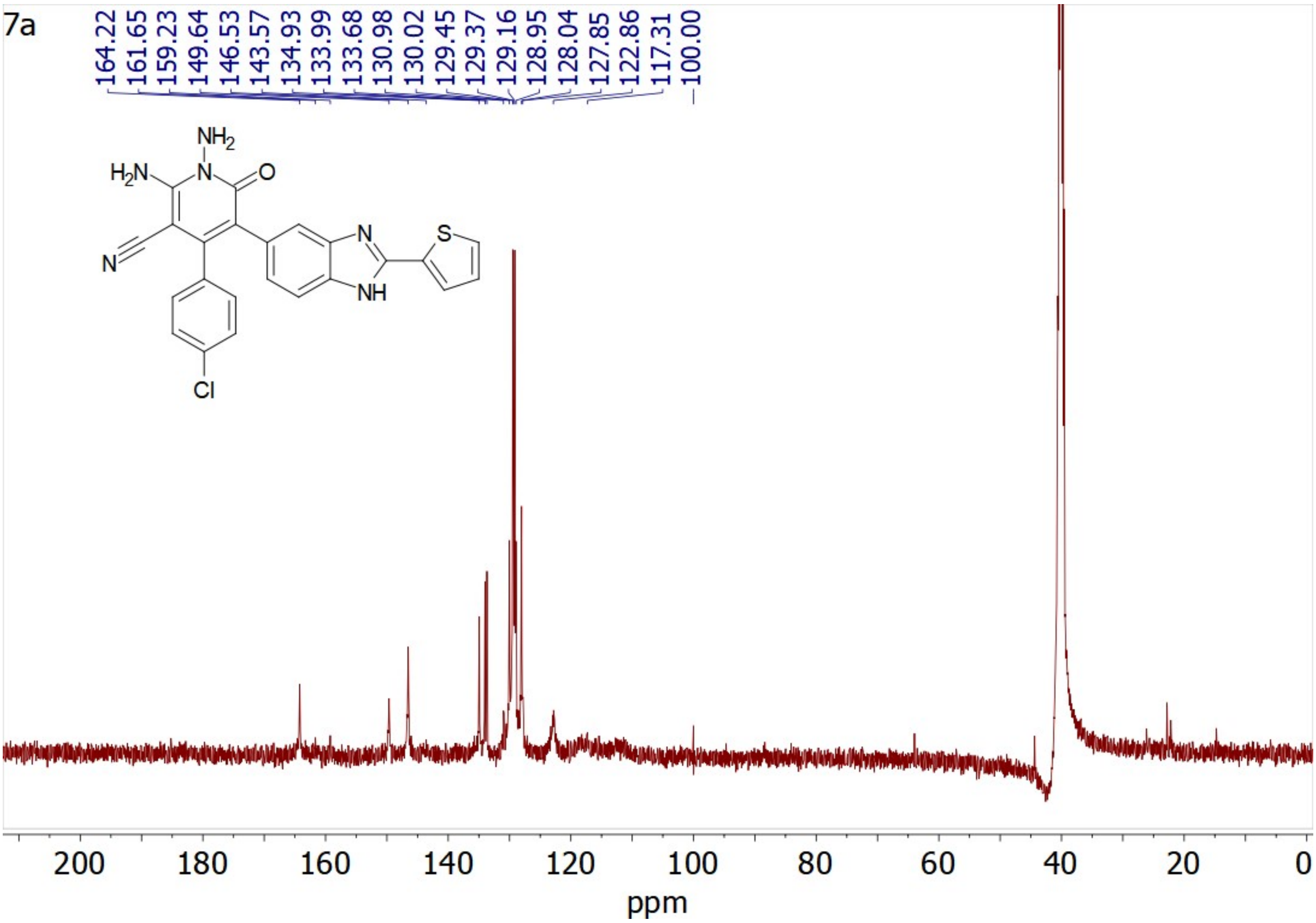

S6: Mass spectrum of compound 7a

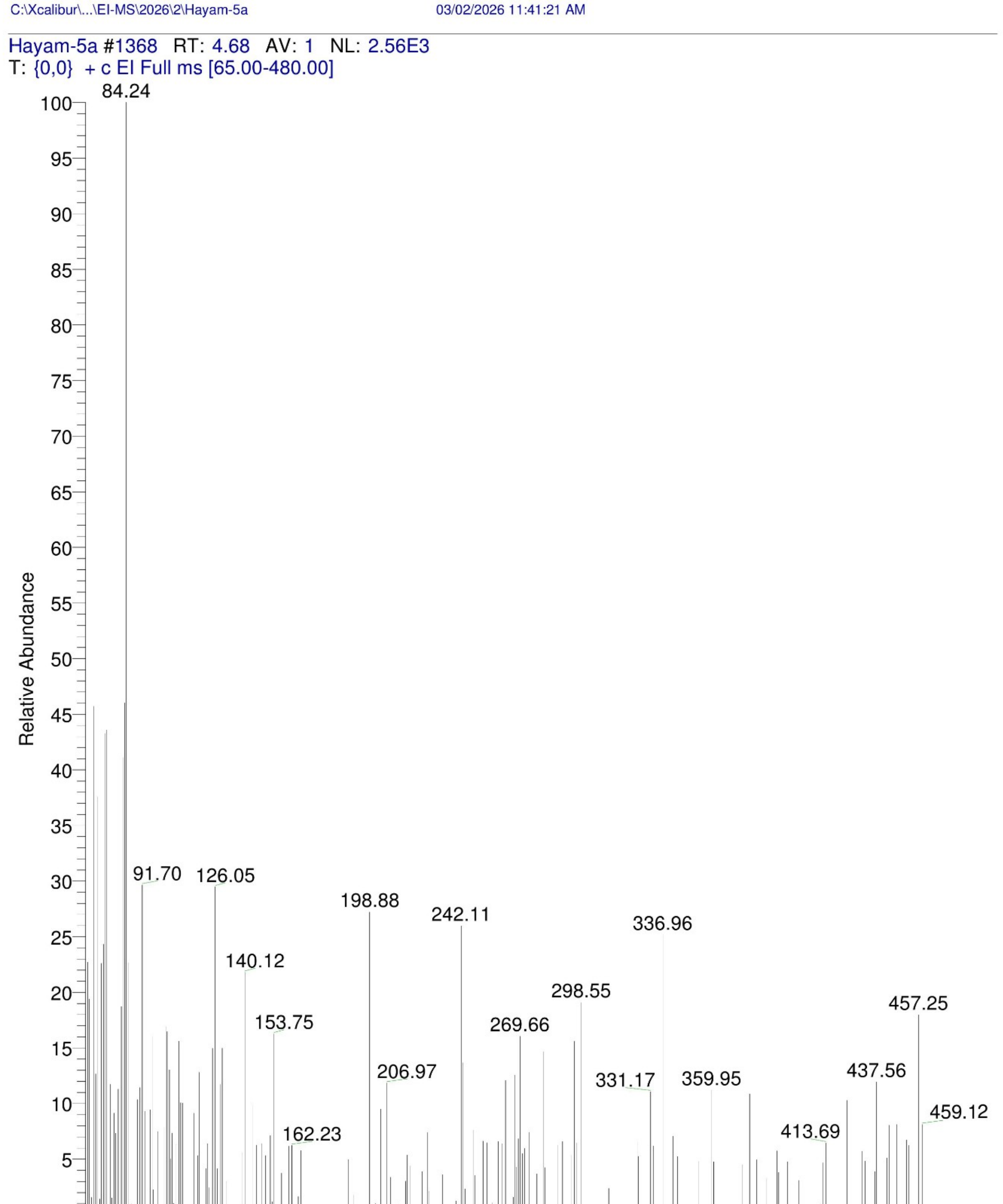

S7: ^{1}H NMR spectrum of compound 7b (400 MHz, DMSO-*d6*)

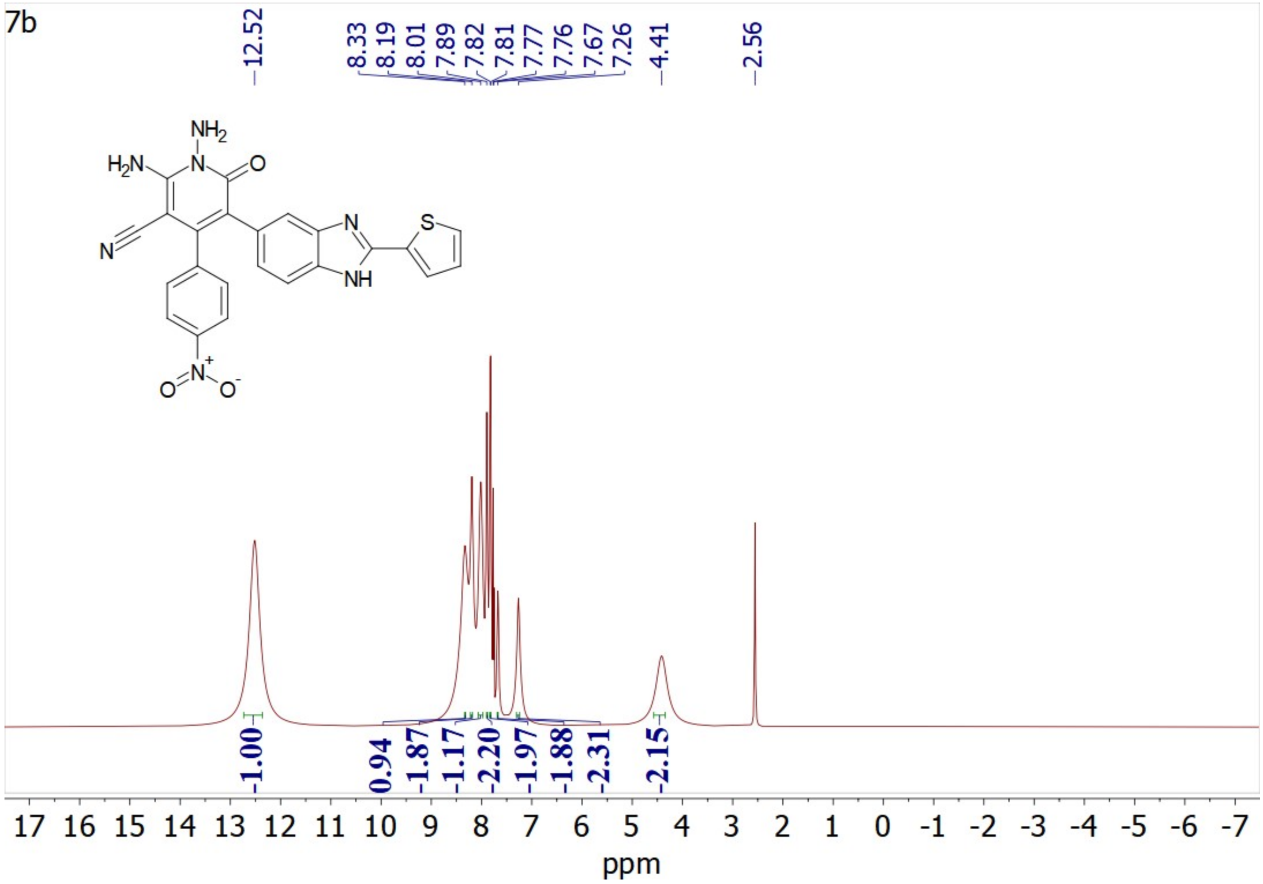

S8: ^{13}C NMR spectrum of compound 7b (100 MHz, DMSO-*d6*)

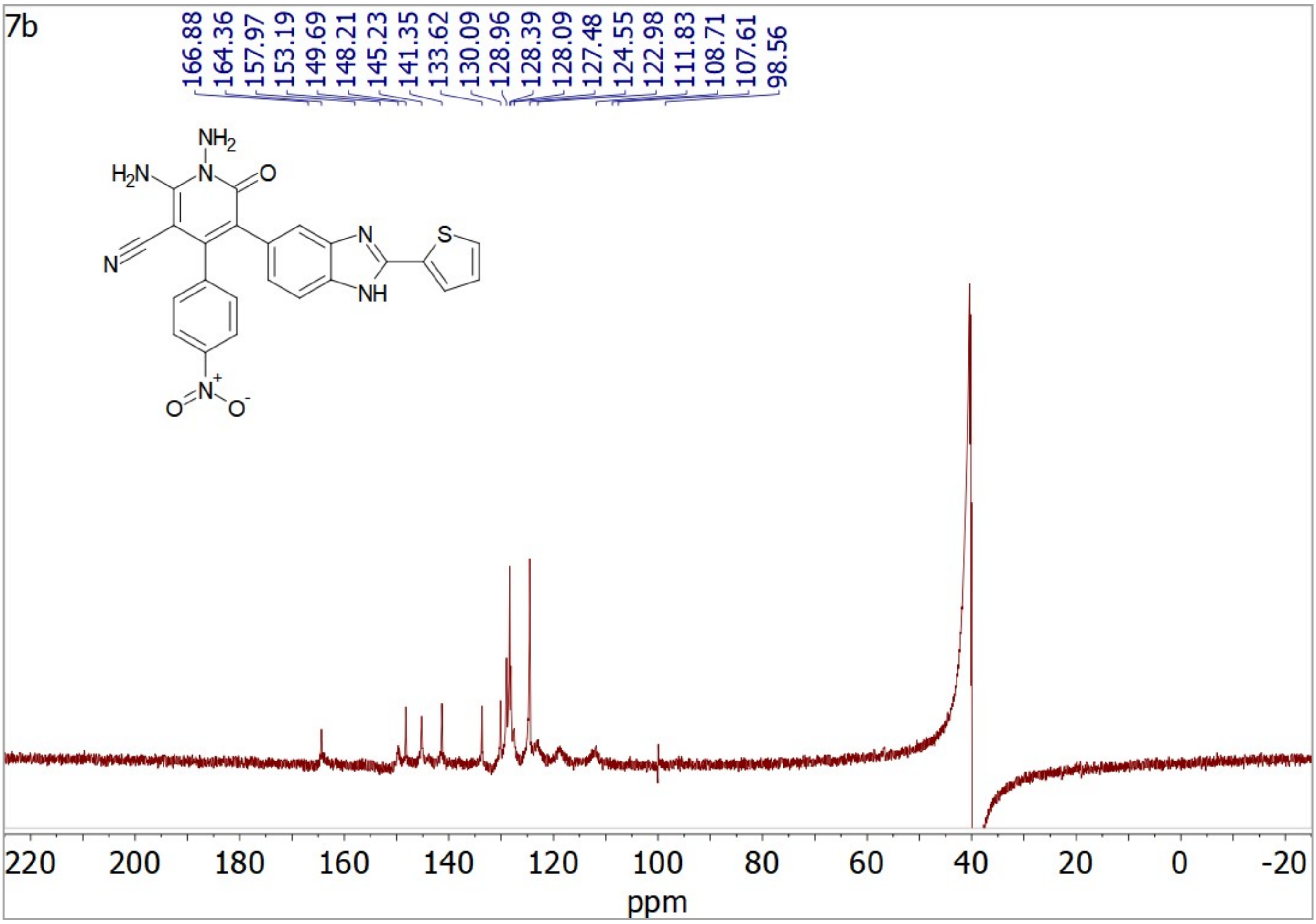

S9: Mass spectrum of compound <u>7b</u>

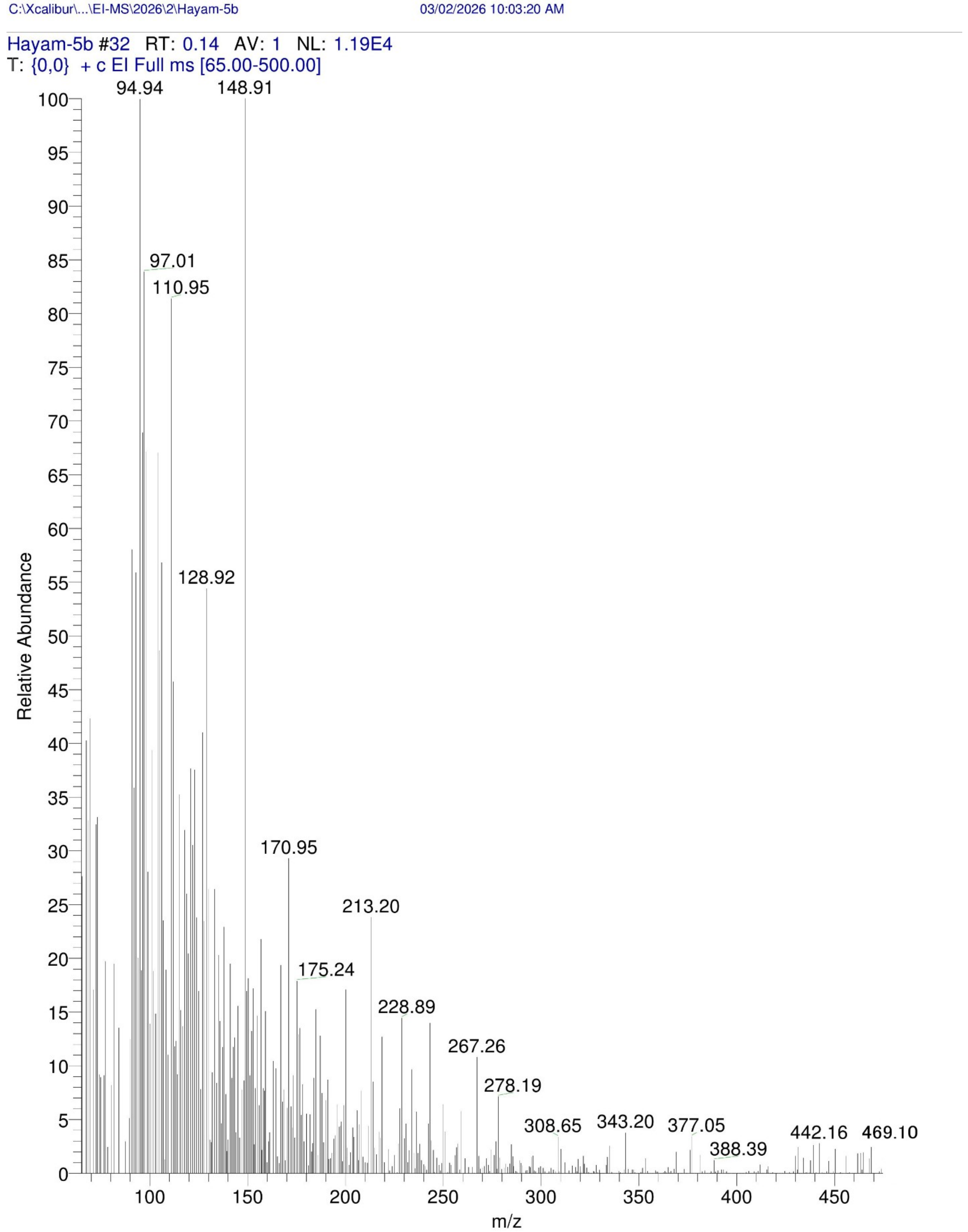

S10: ^{1}H NMR spectrum of compound <u>7c</u> (400 MHz, DMSO-*d6*)

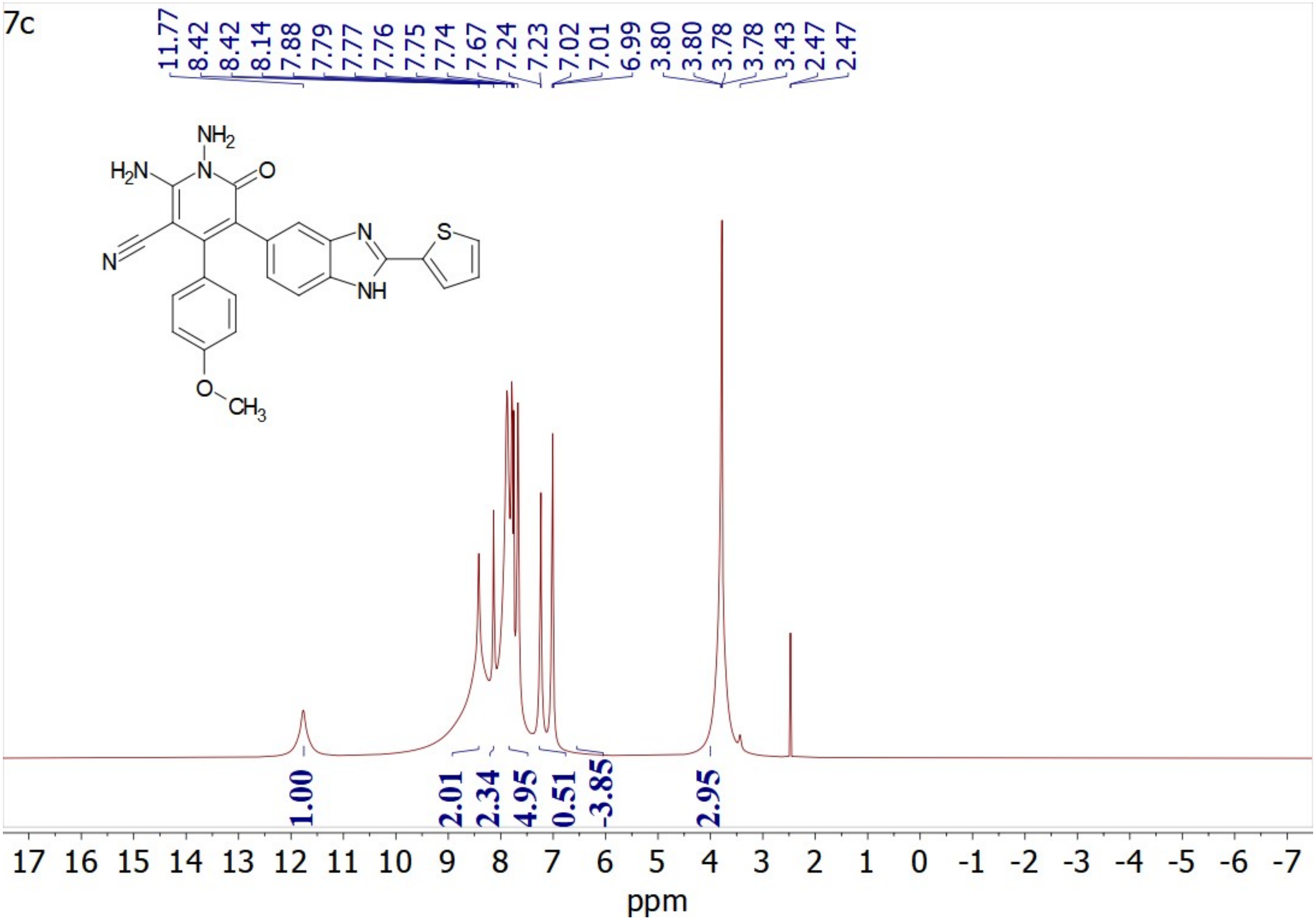

S11: ^{13}C NMR spectrum of compound 7c (100 MHz, DMSO-*d6*)

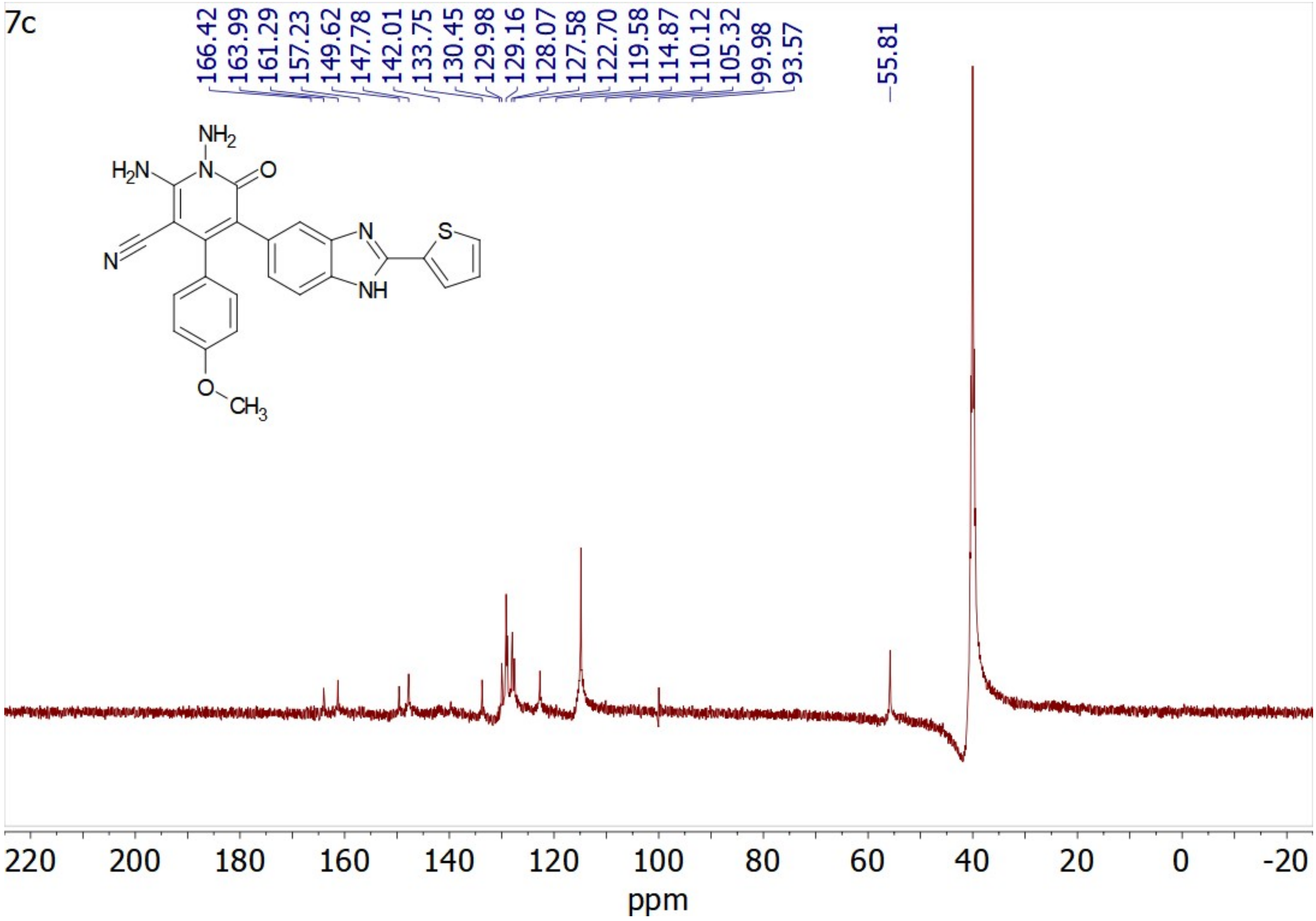

S12: Mass spectrum of compound 7c

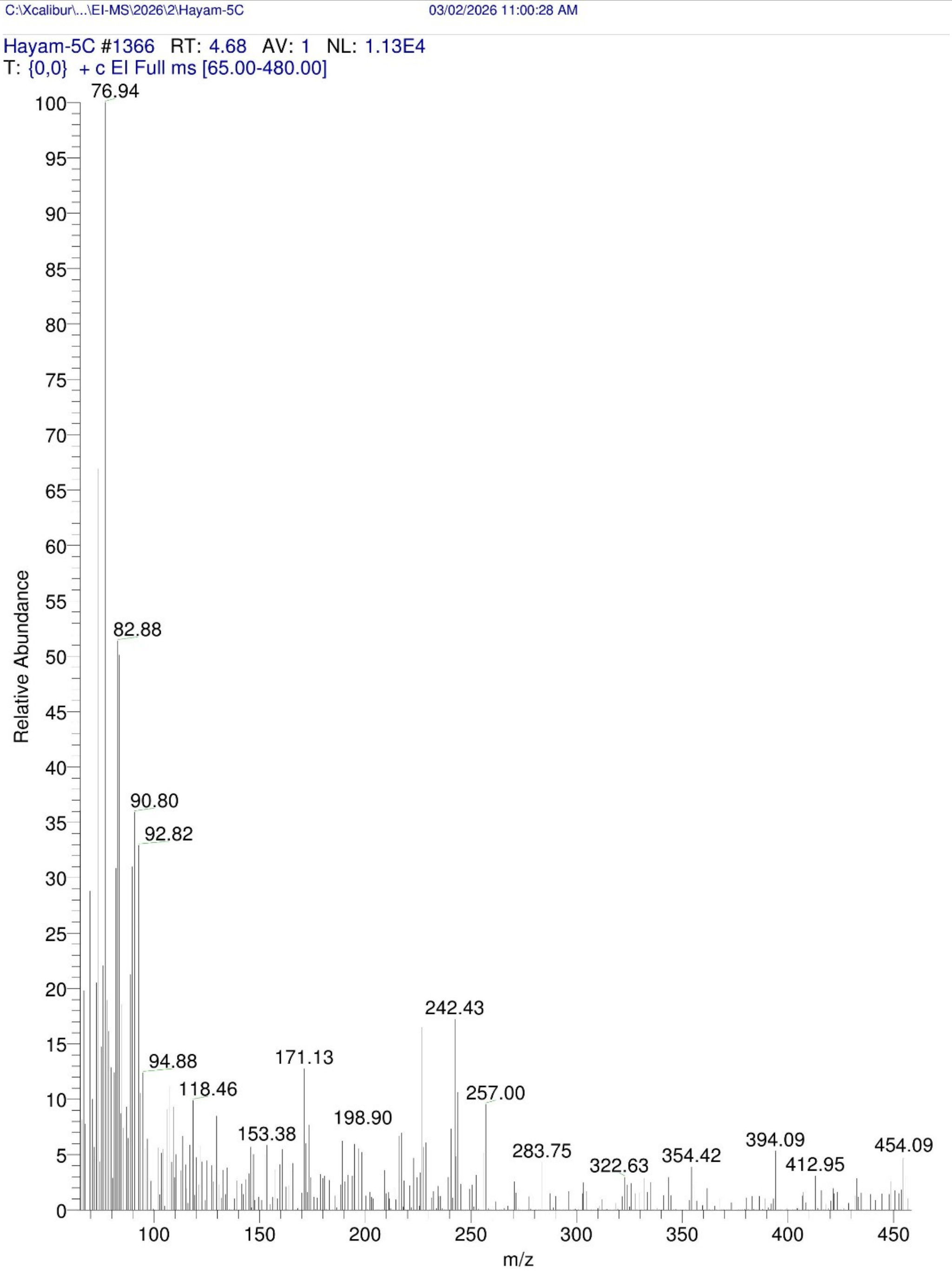

S13: ^{1}H NMR spectrum of compound 7d (400 MHz, DMSO-*d6*)

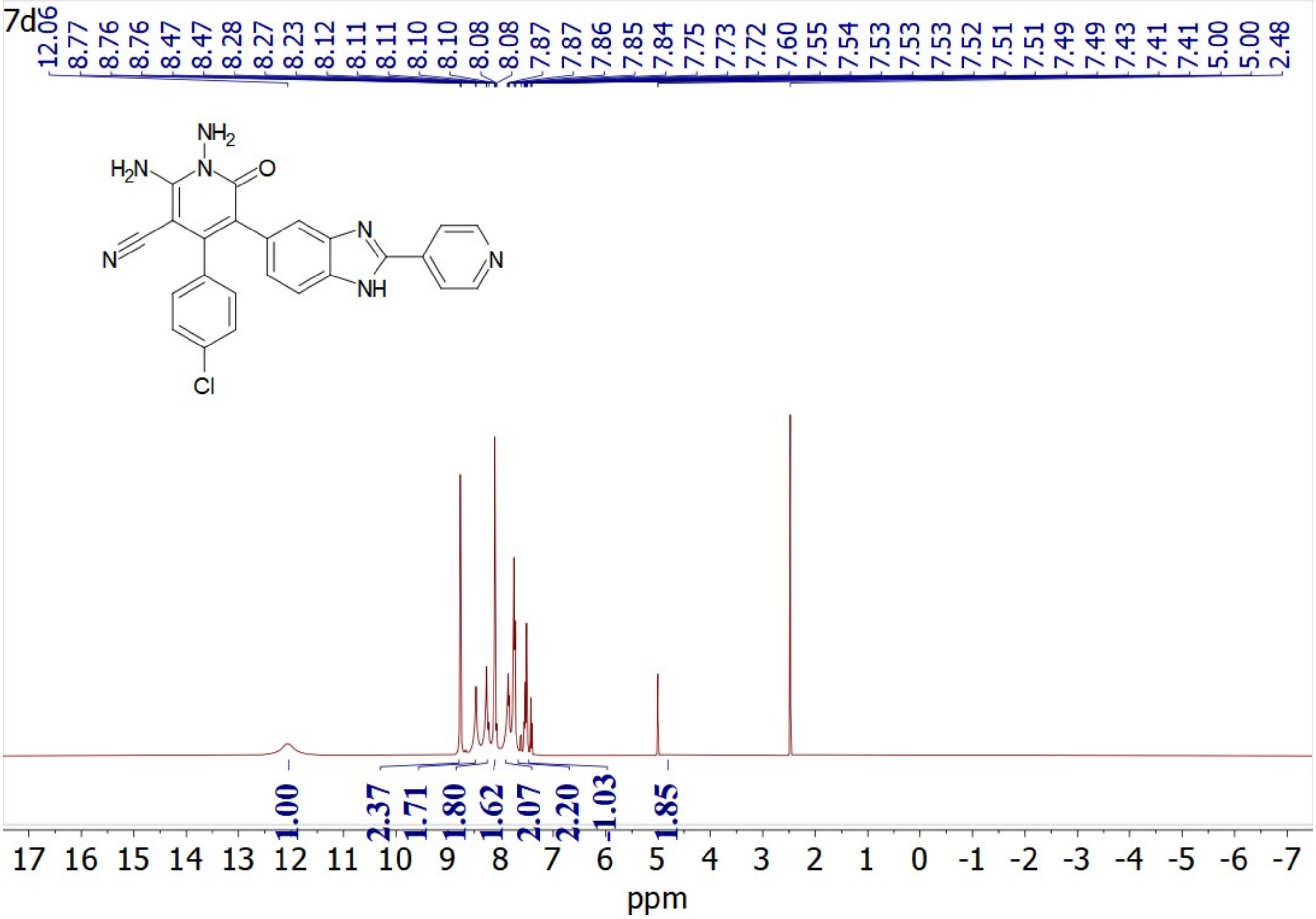

S14: ^{13}C NMR spectrum of compound 7d (100 MHz, DMSO-*d6*)

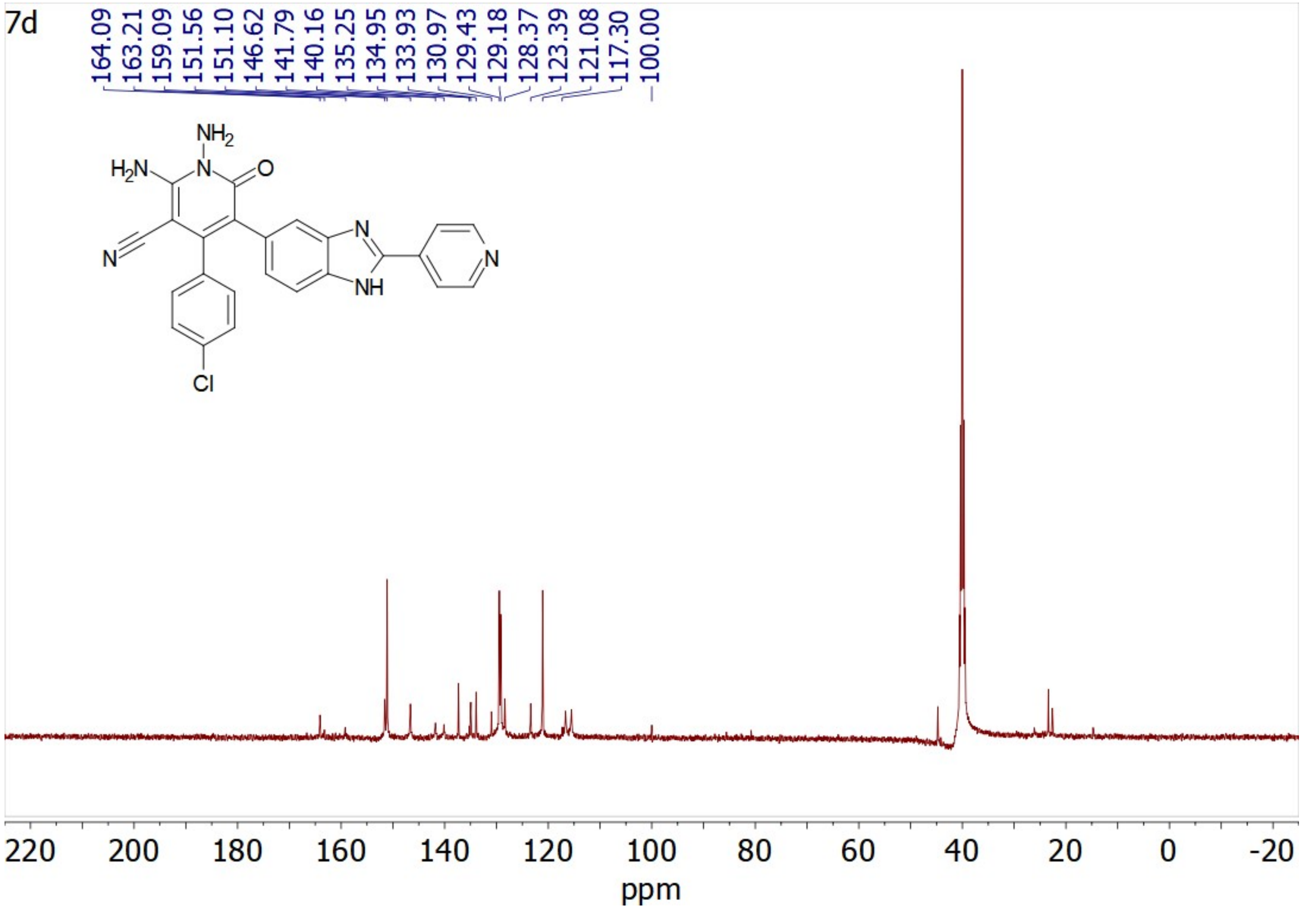

S15: Mass spectrum of compound 7d

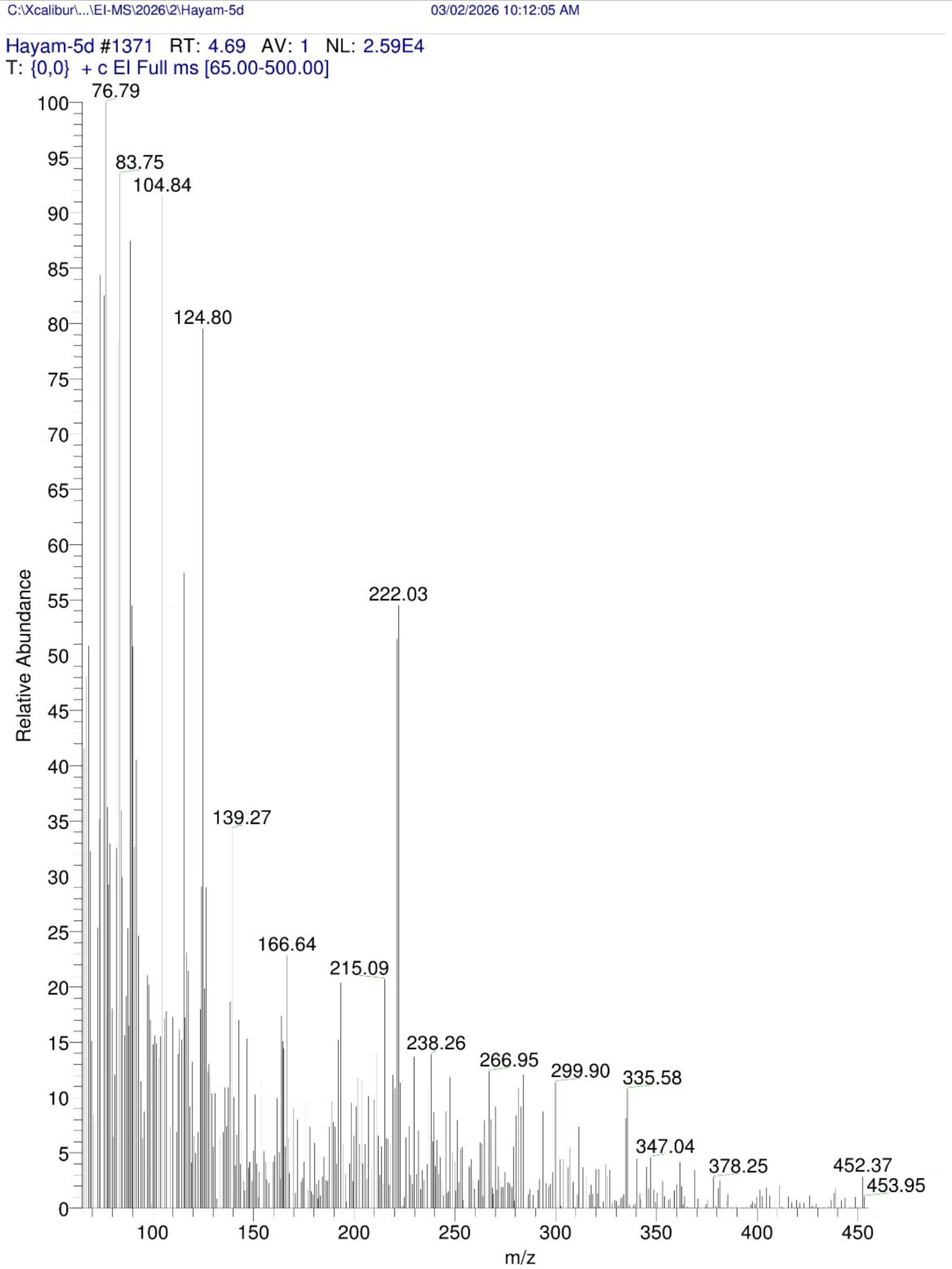

S16: ^{1}H NMR spectrum of compound <u>7e</u> (500 MHz, DMSO-*d6*)

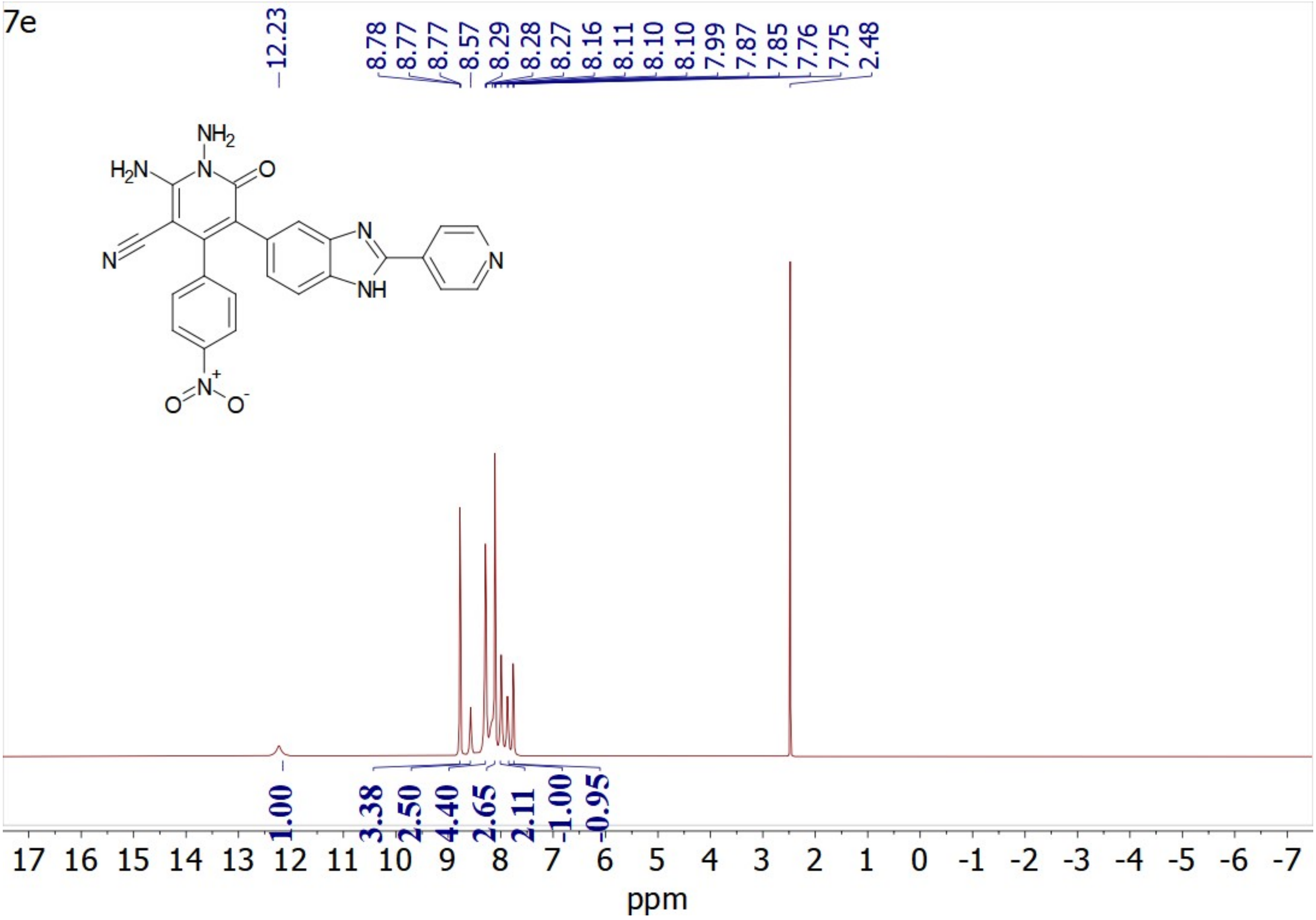

S17: ^{13}C NMR spectrum of compound <u>7e</u> (125 MHz, DMSO-*d6*)

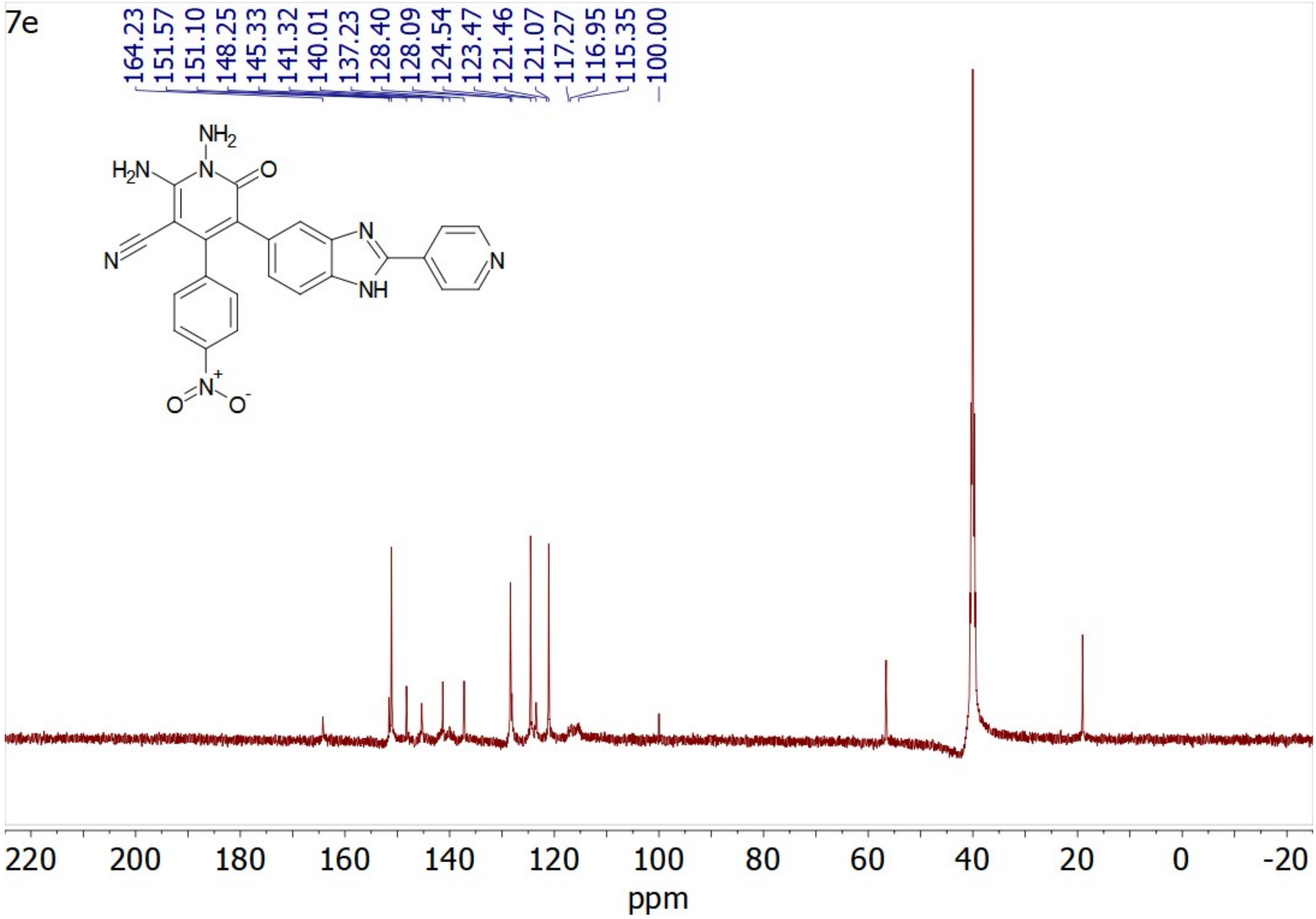

S18: Mass spectrum of compound 7e

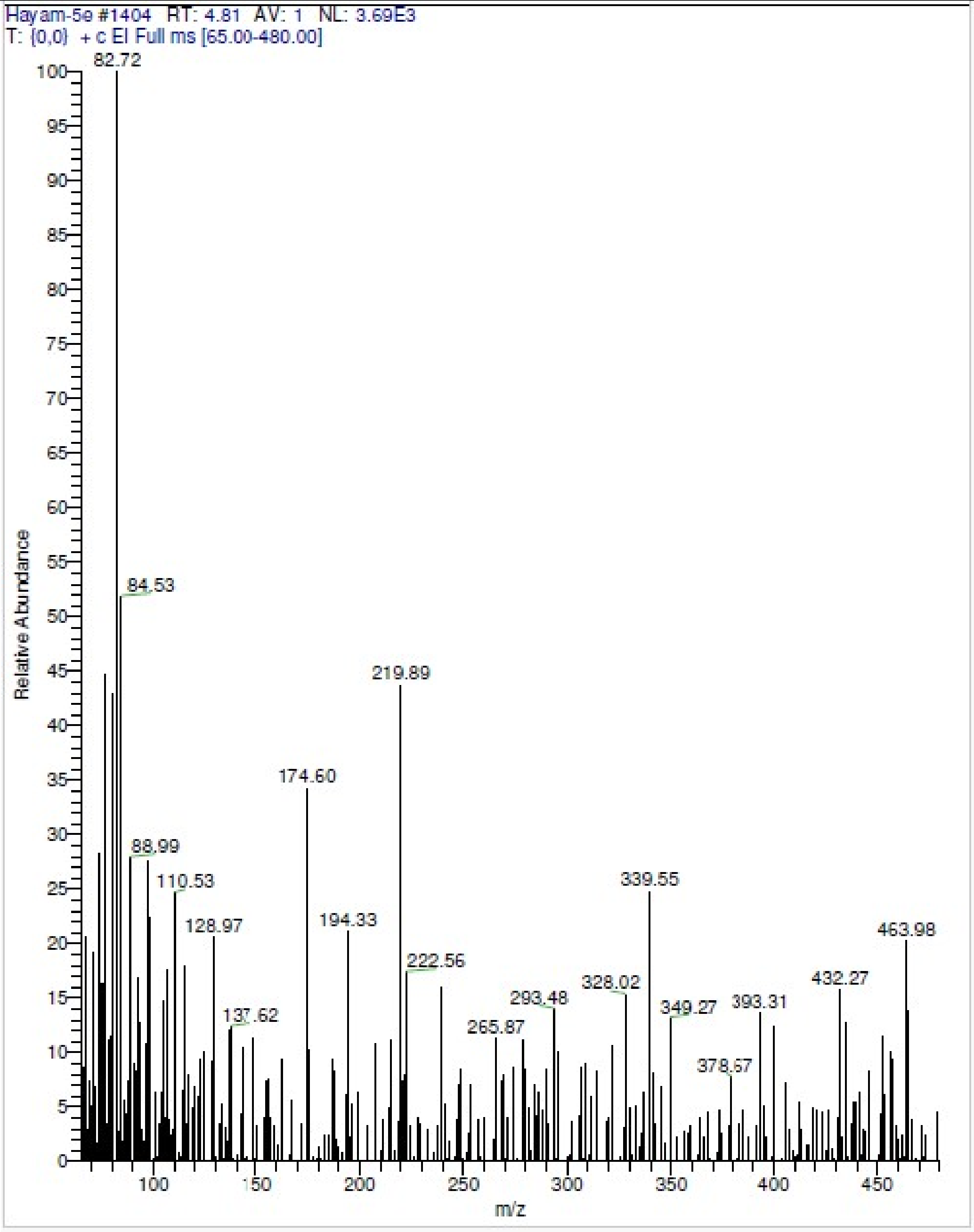

S19: ^{1}H NMR spectrum of compound 7f (400 MHz, DMSO-*d6*)

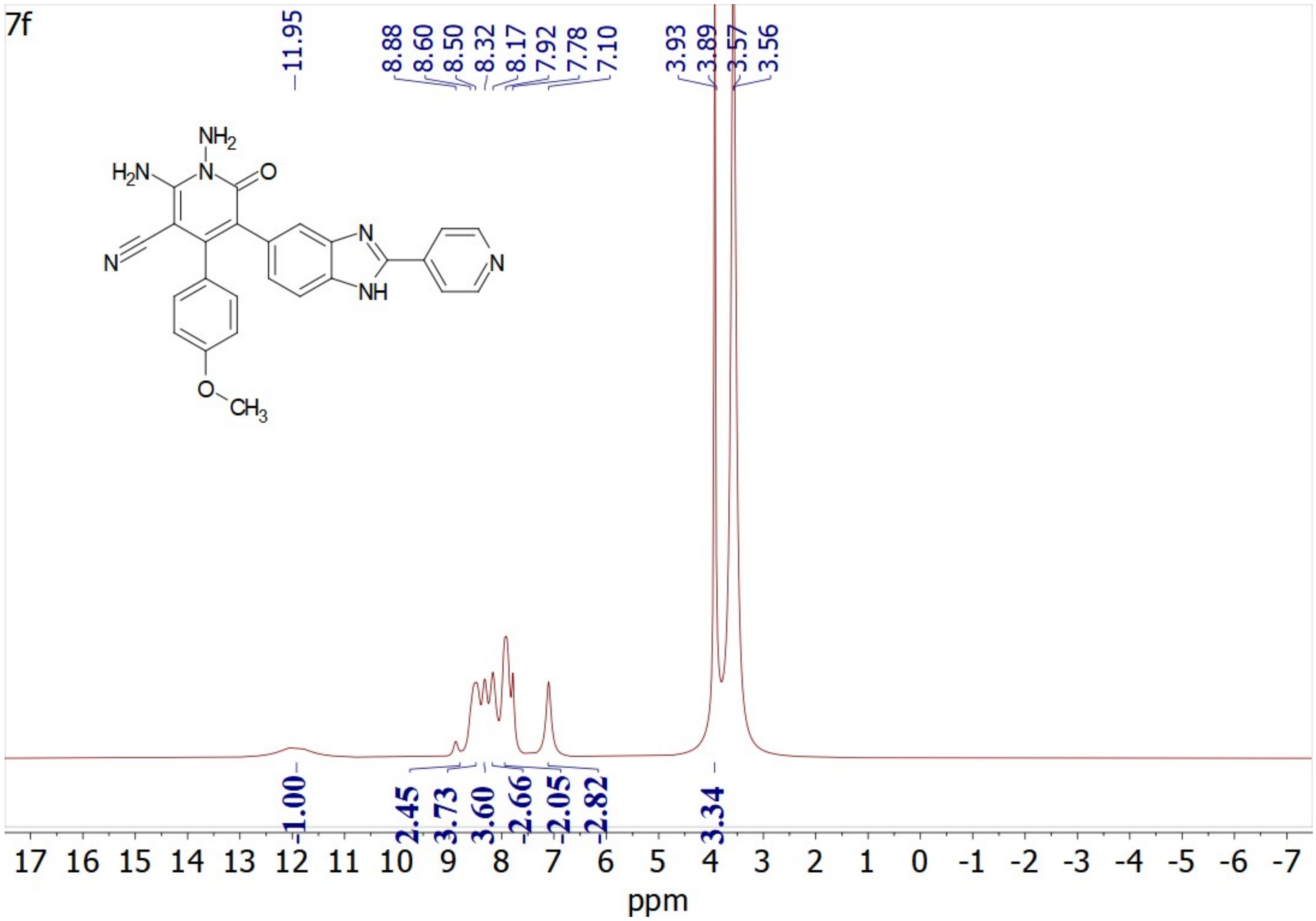

S20: ^{13}C NMR spectrum of compound 7f (100 MHz, DMSO-*d6*)

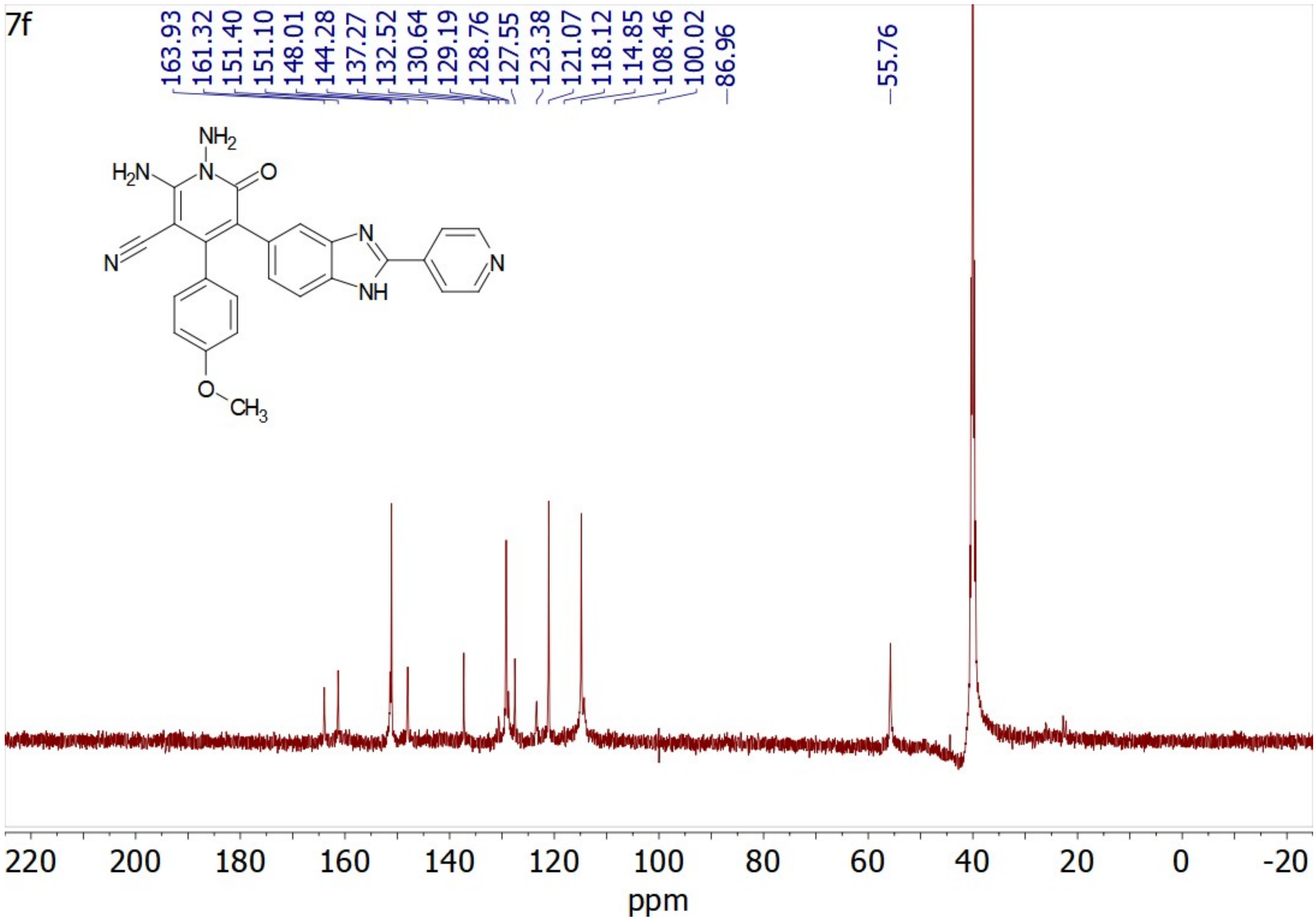

S21: Mass spectrum of compound 7f

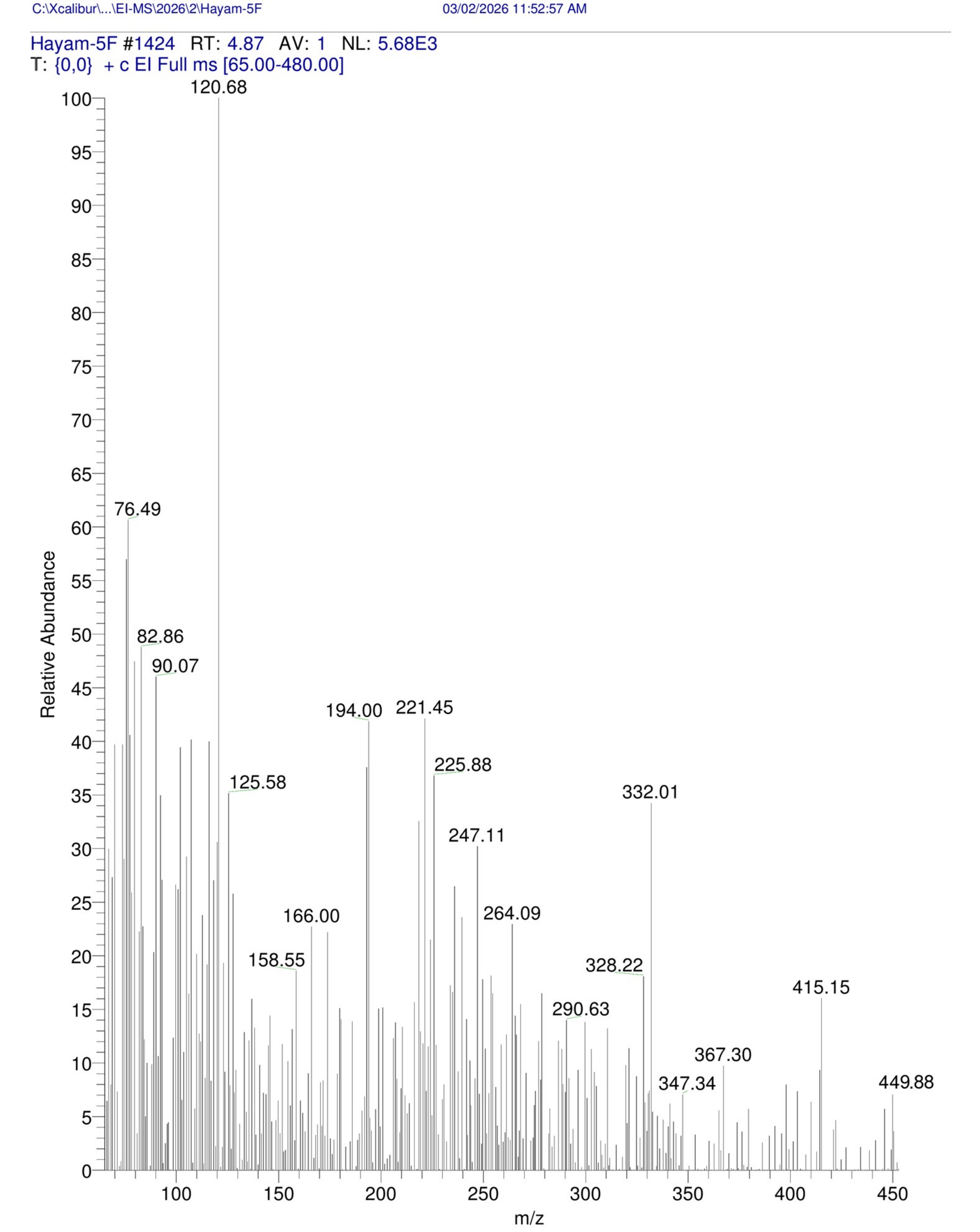

S22: ^{1}H NMR spectrum of compound 8a (400 MHz, DMSO-*d6*)

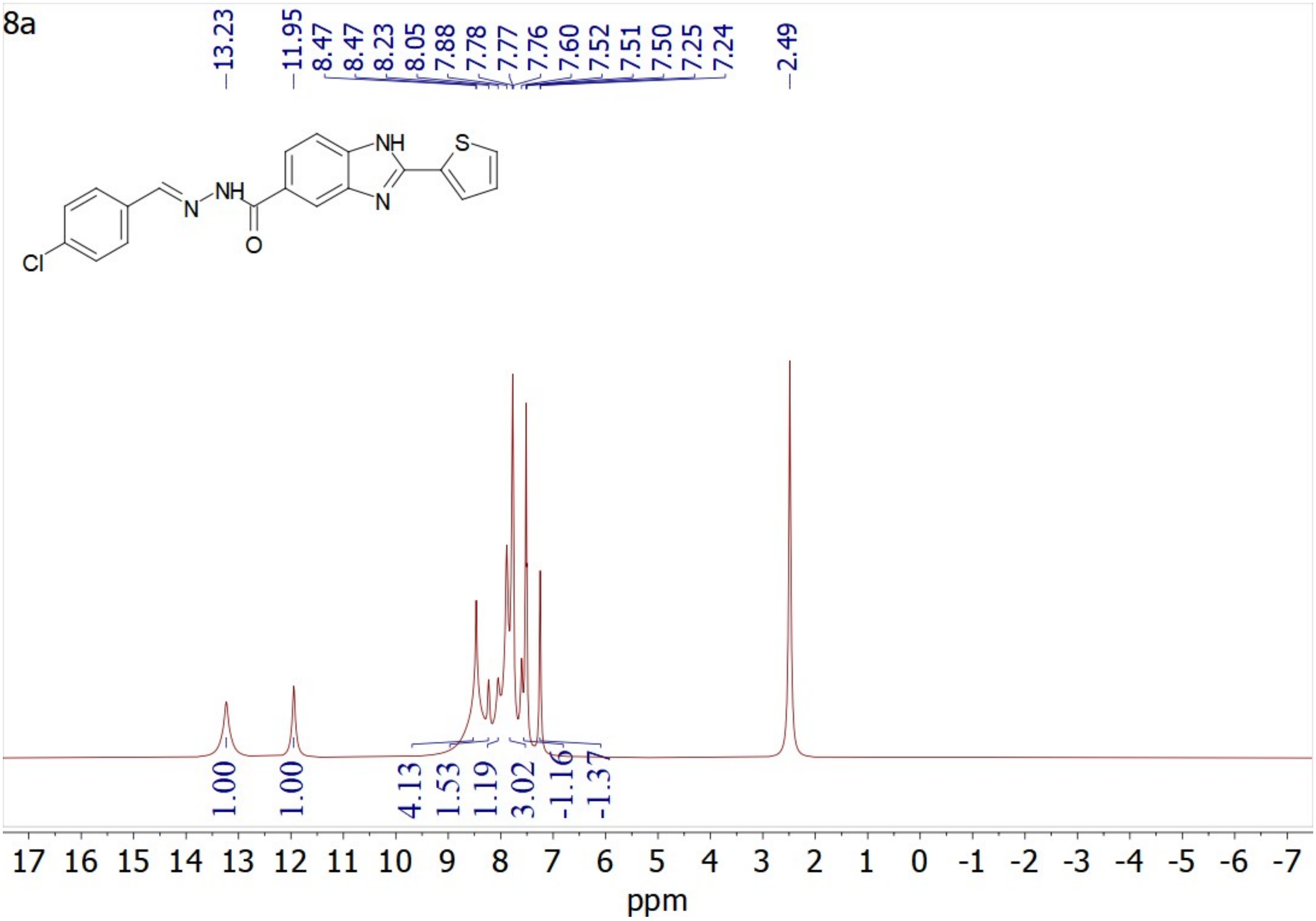

S23: ^{13}C NMR spectrum of compound <u>8a</u> (100 MHz, DMSO-*d6*)

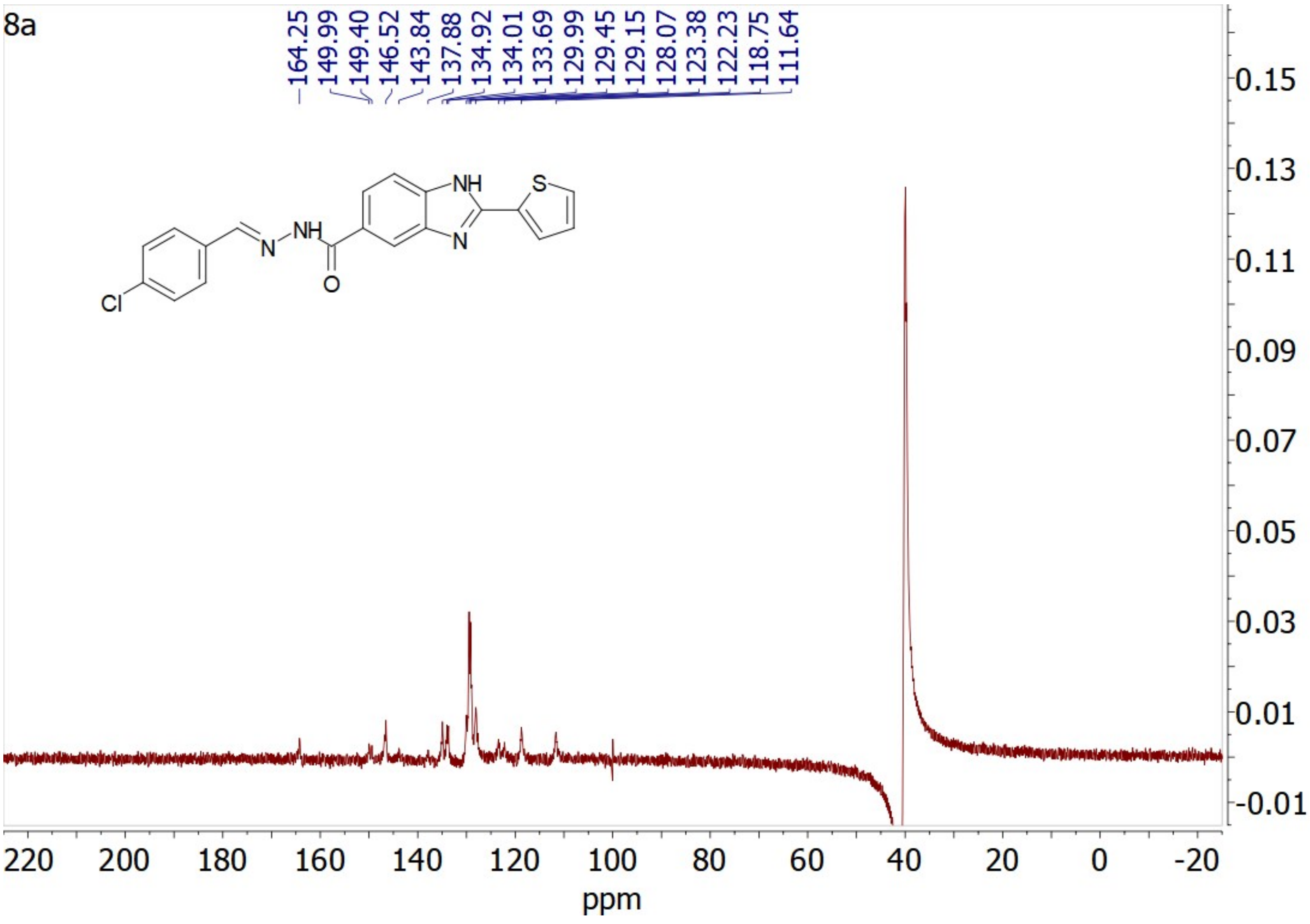

S24: Mass spectrum of compound 8a

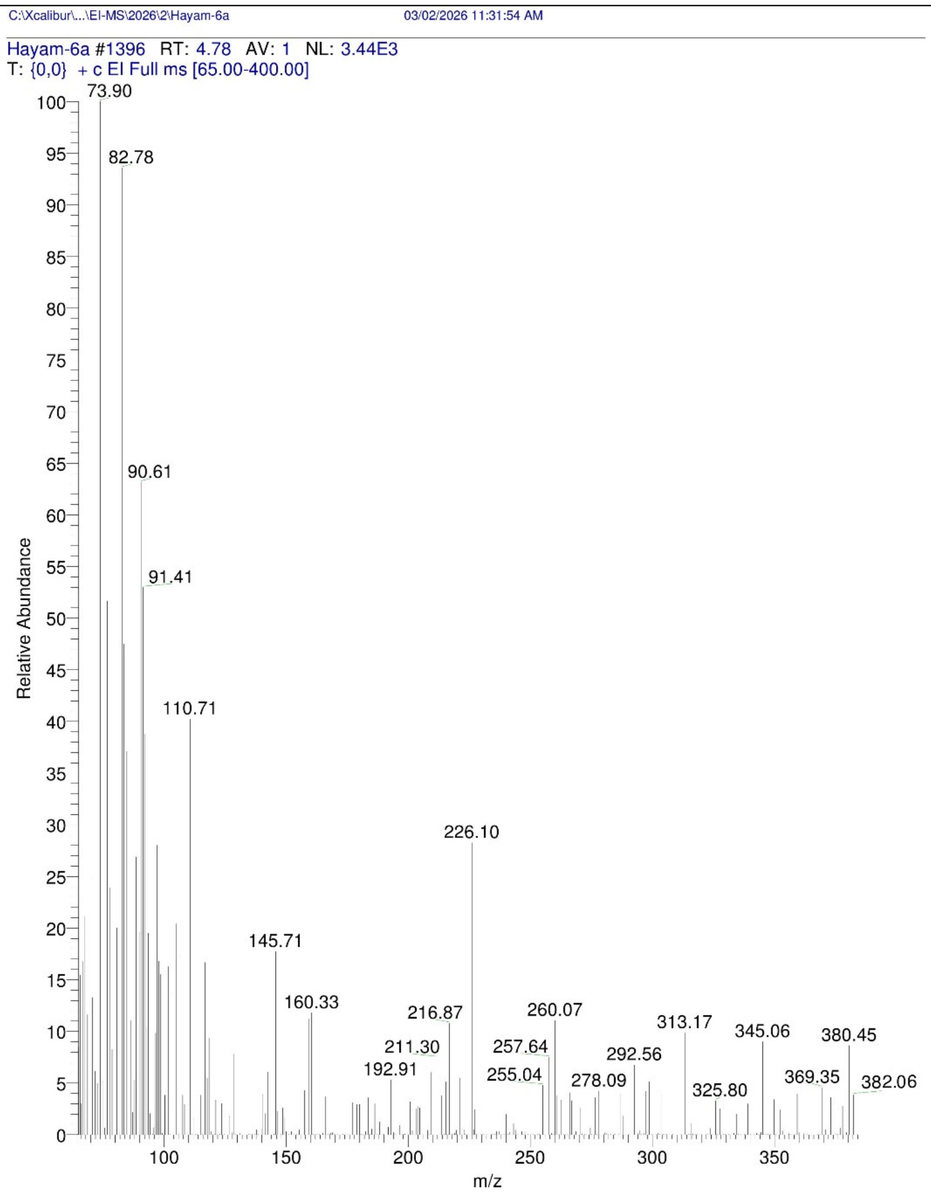

S25: ^{1}H NMR spectrum of compound 8b (400 MHz, DMSO-*d6*)

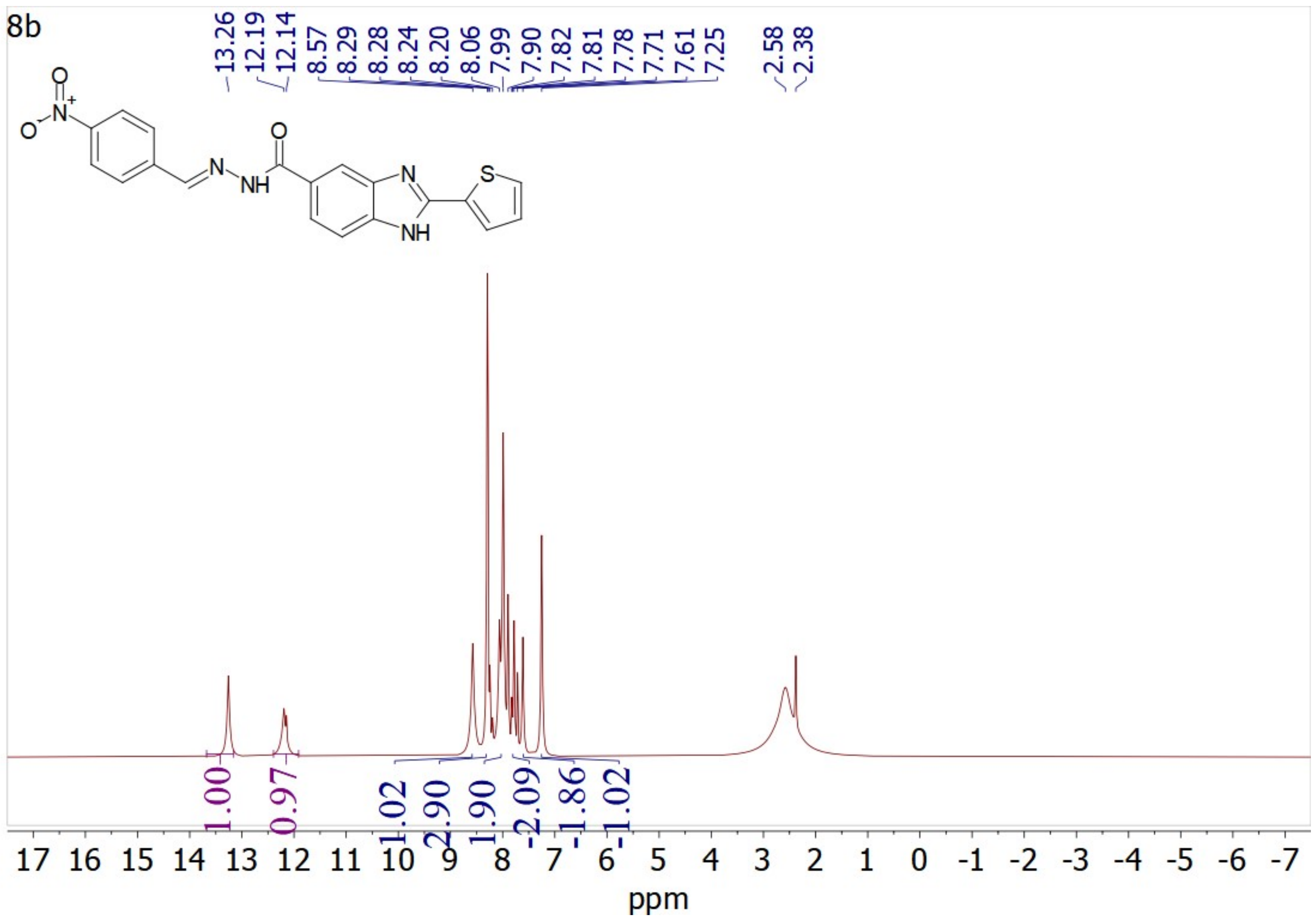

S26: ^{13}C NMR spectrum of compound 8b (100 MHz, DMSO-*d6*)

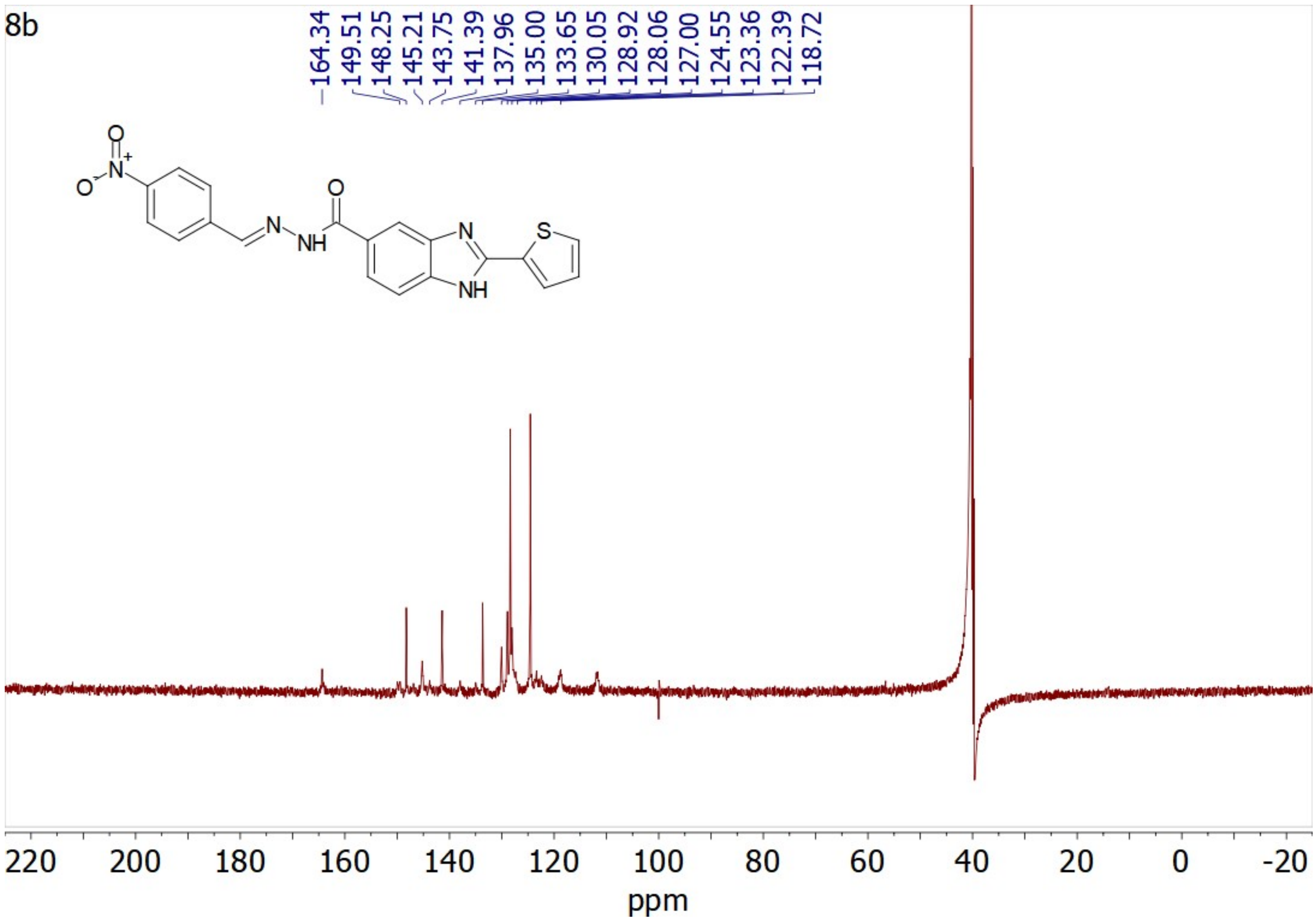

S27: Mass spectrum of compound 8b

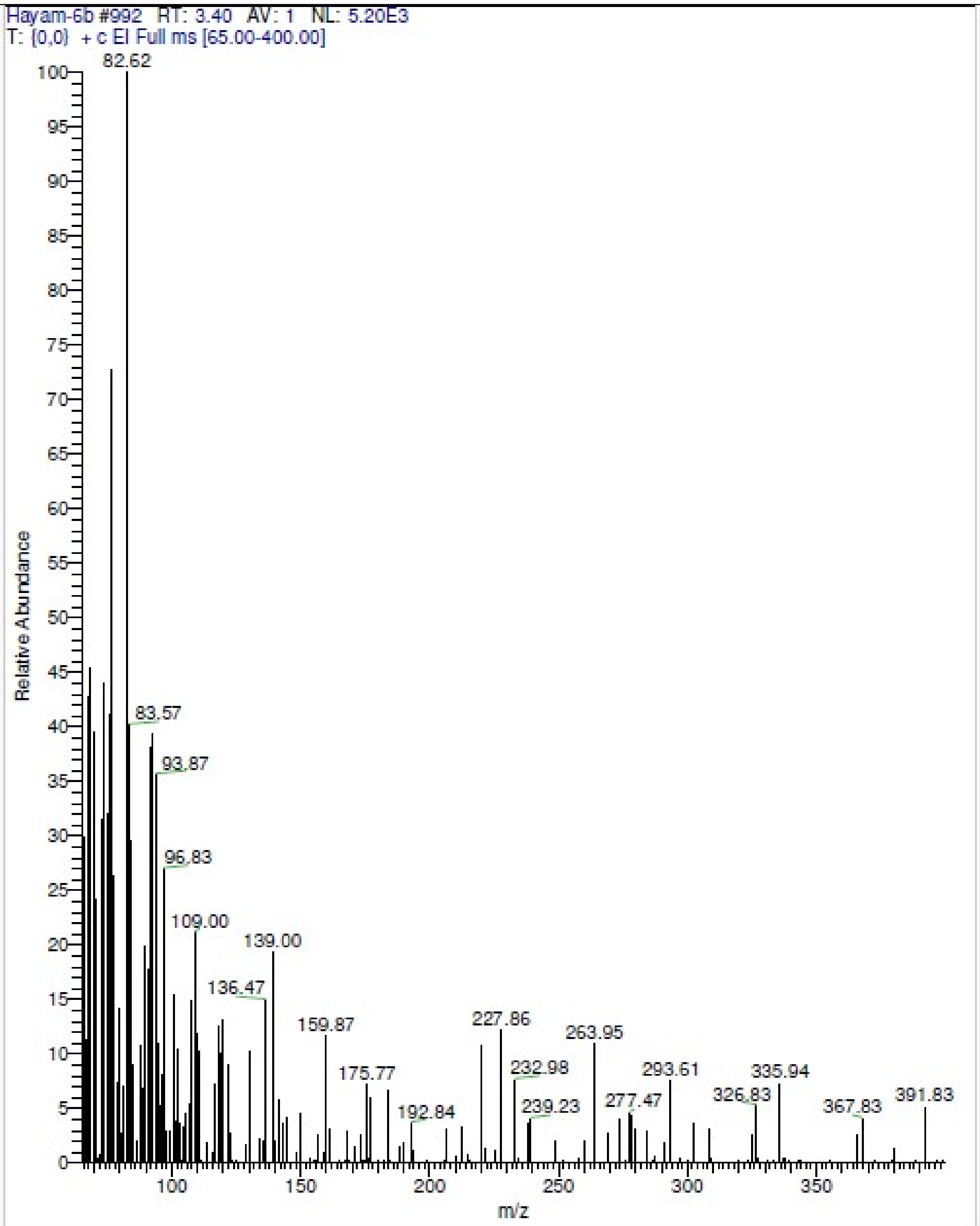

S28: ^{1}H NMR spectrum of compound 8c (400 MHz, DMSO-*d6*)

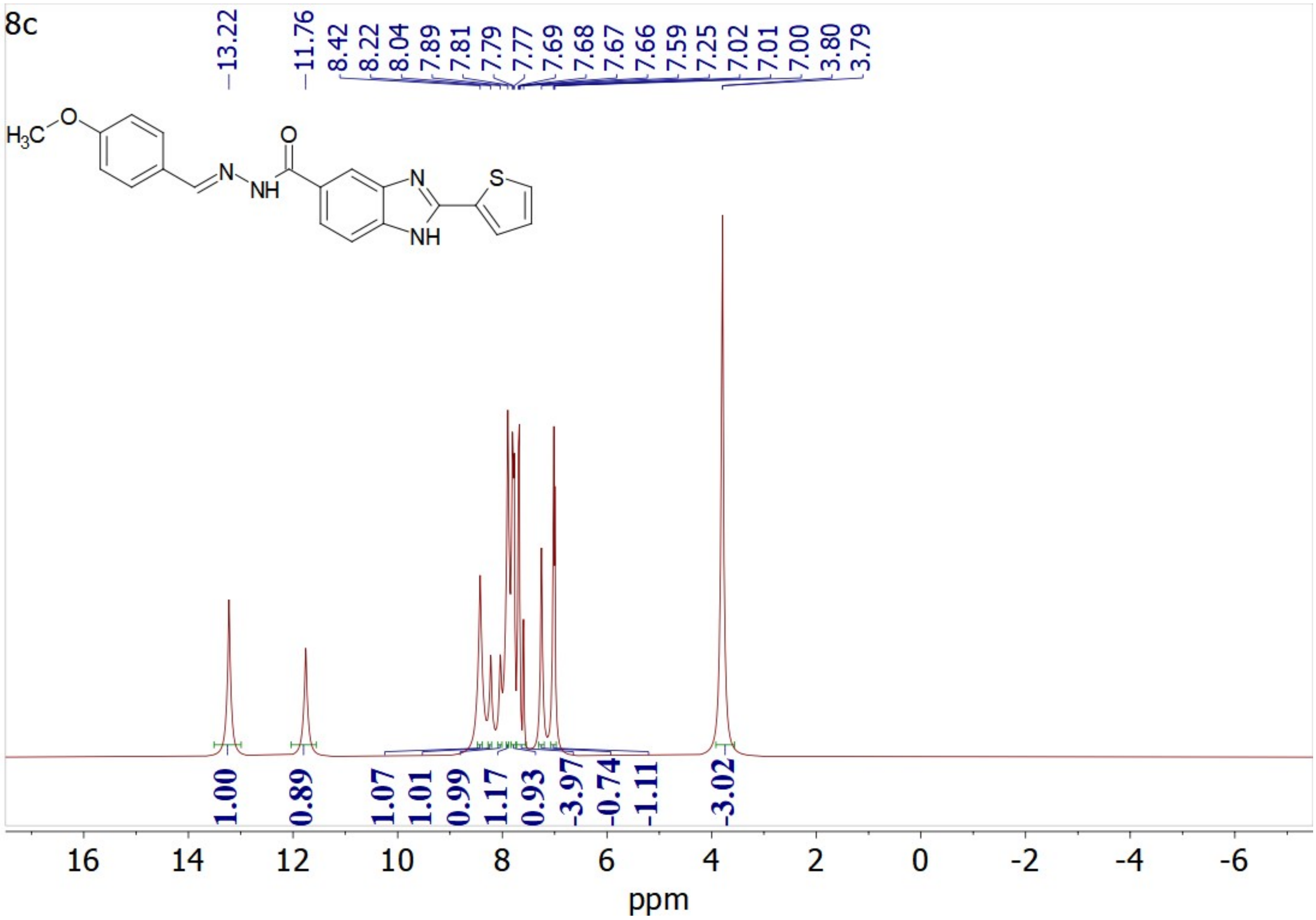

S29: ^{13}C NMR spectrum of compound <u>8c</u> (100 MHz, DMSO-*d6*)

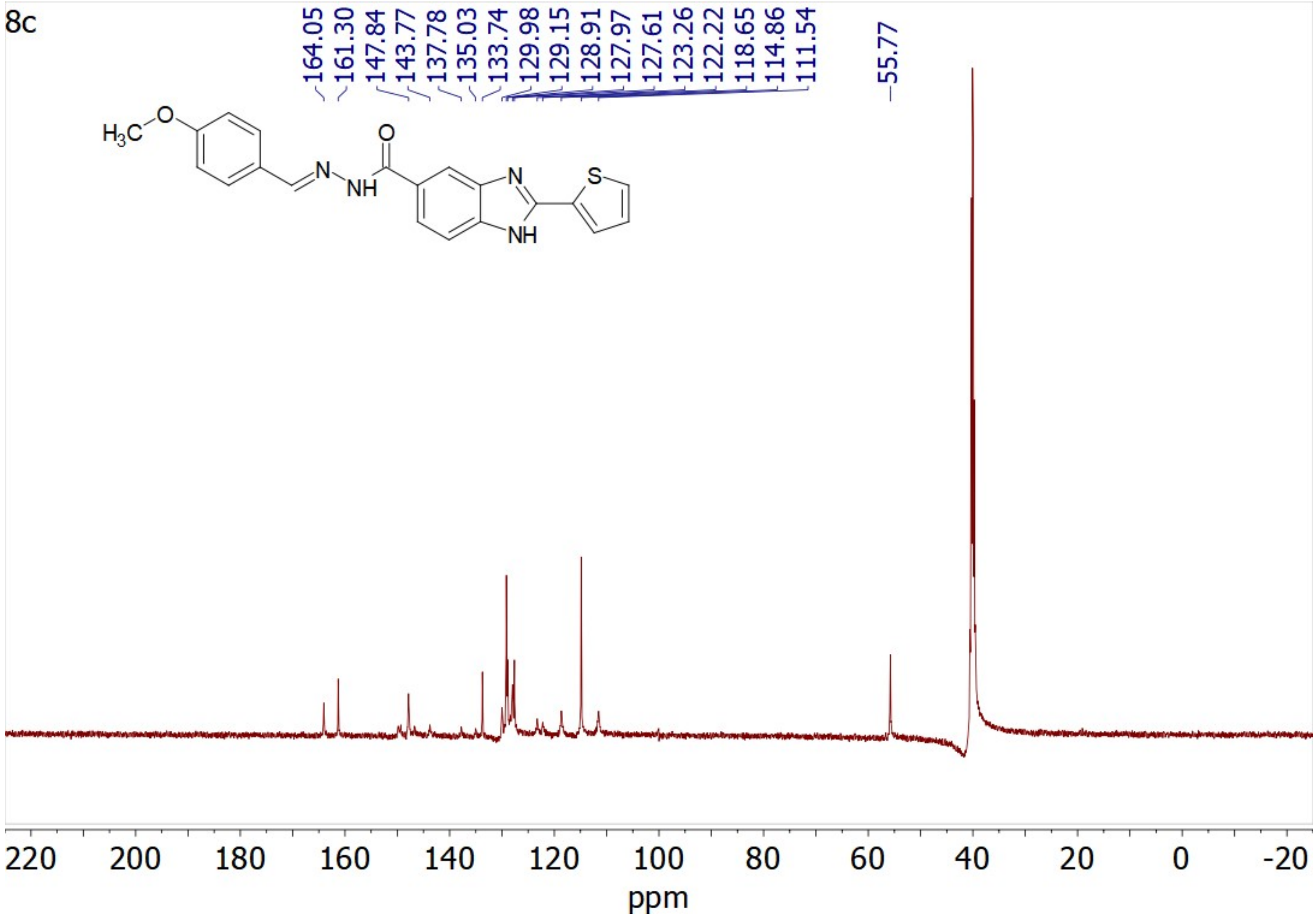

S30: Mass spectrum of compound <u>8c</u>

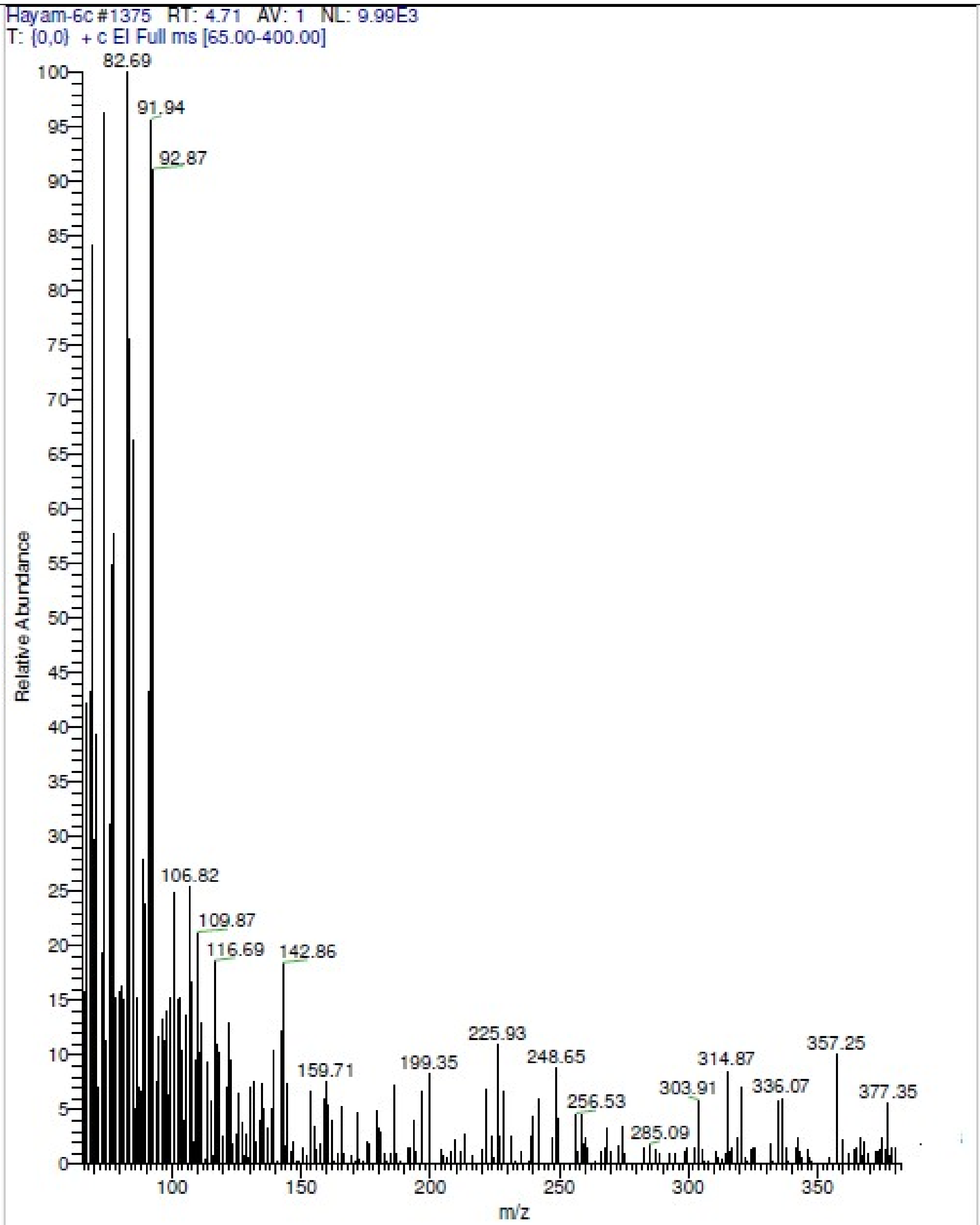

S31: ^{1}H NMR spectrum of compound 8d (400 MHz, DMSO-*d6*)

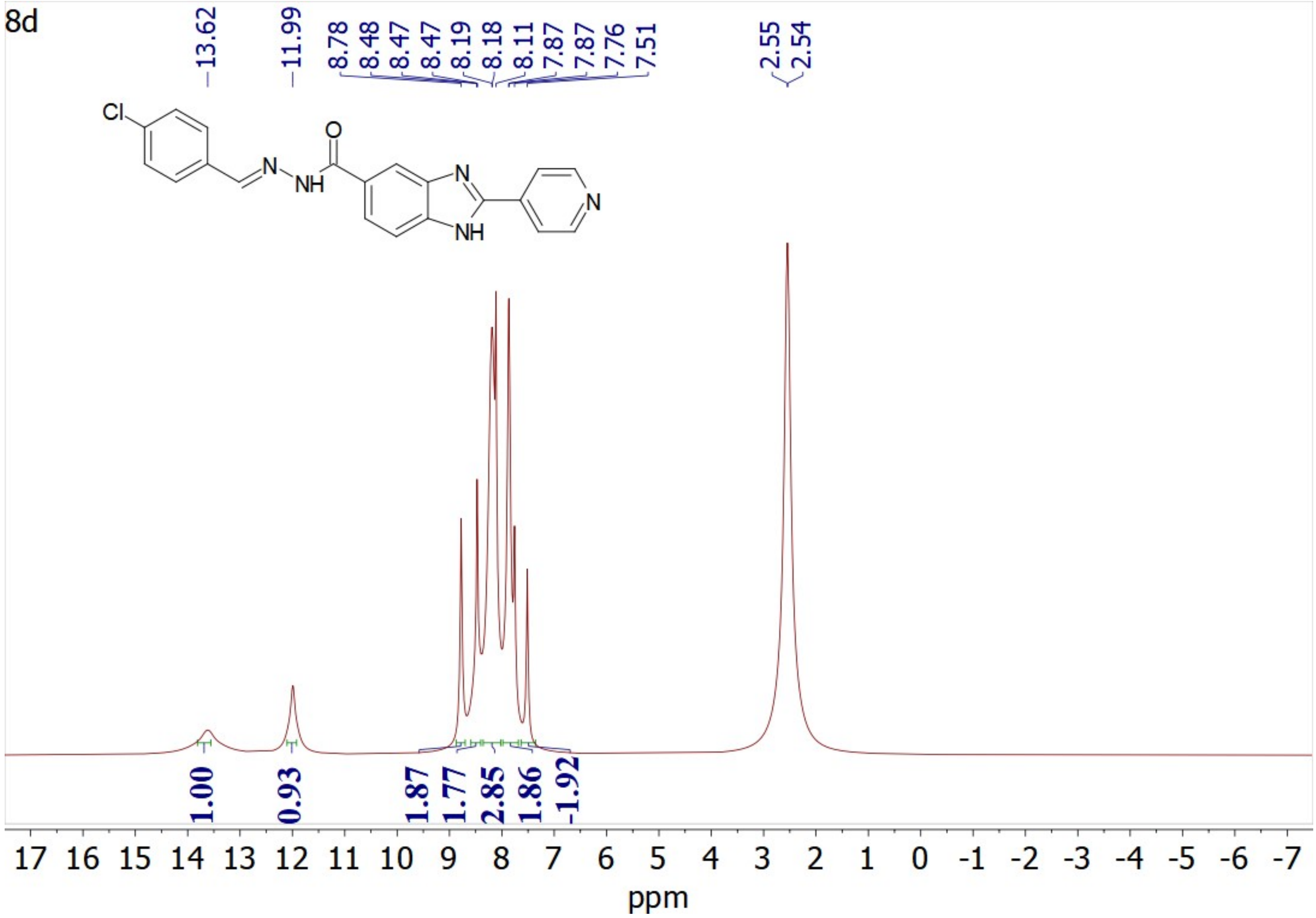

S32: ^{1}H NMR spectrum of compound 8d (400 MHz, DMSO-*d6*)

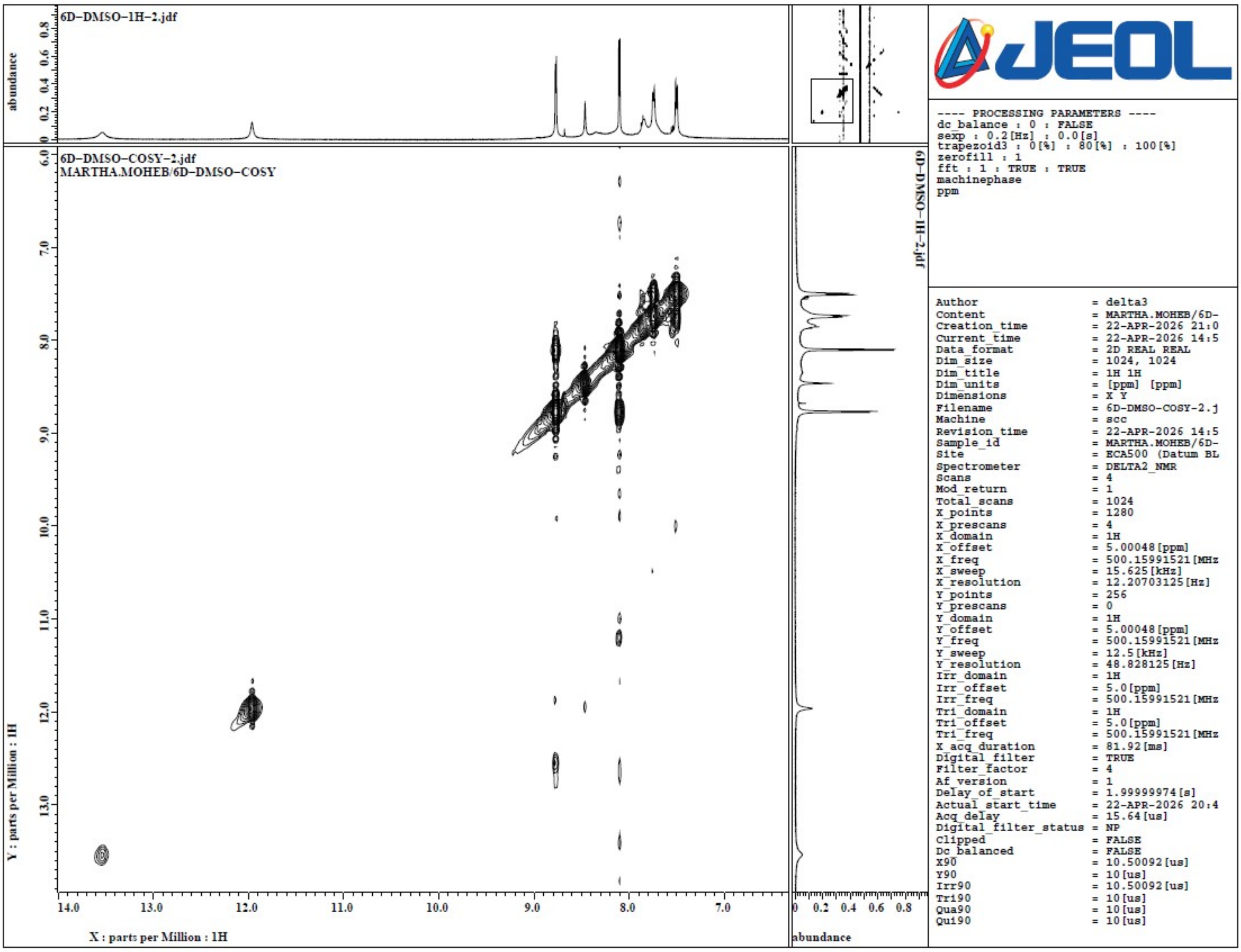

S33: ^{13}C NMR spectrum of compound <u>8d</u> (100 MHz, DMSO-*d6*)

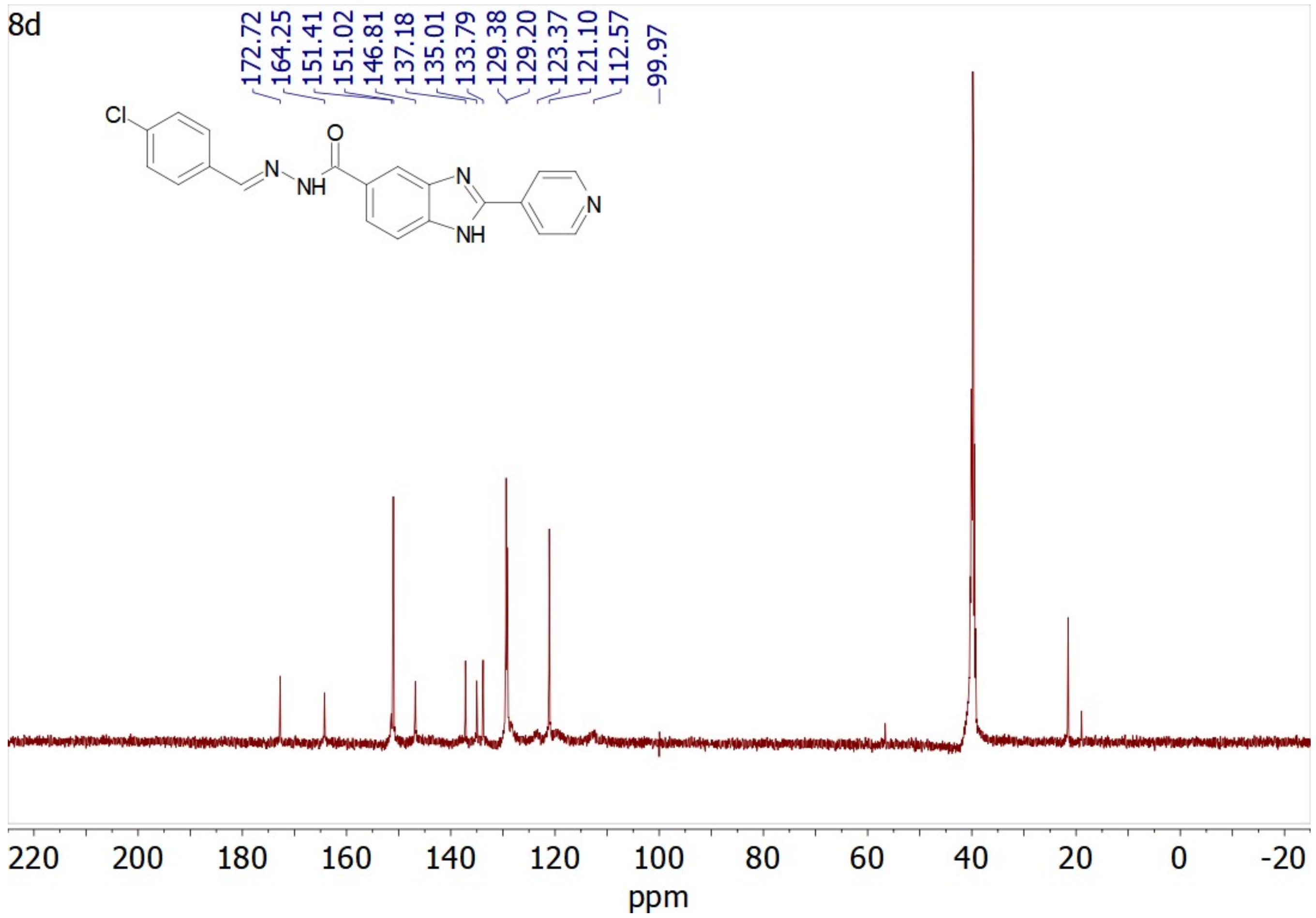

S34: Mass spectrum of compound 8d

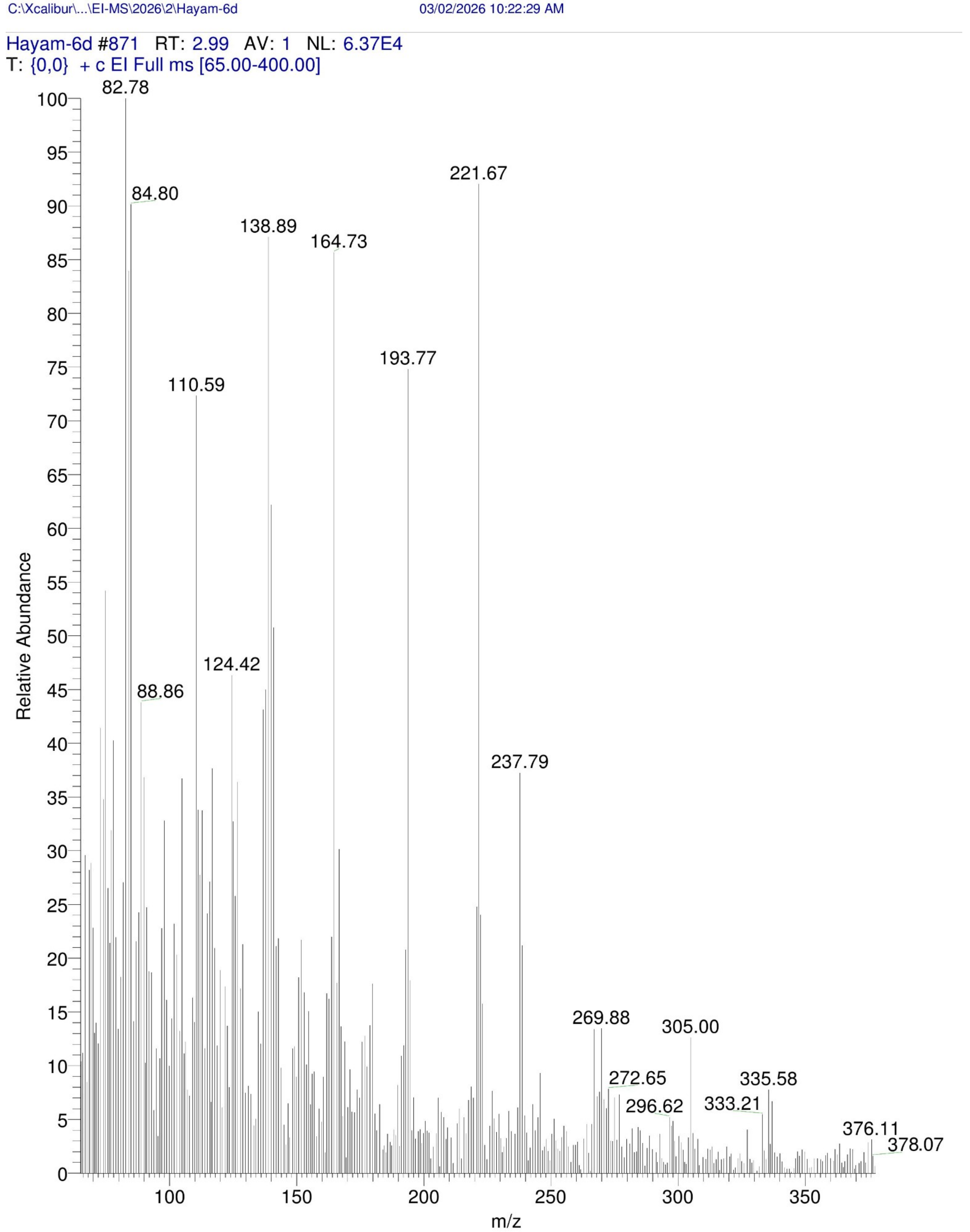

S35: ^{1}H NMR spectrum of compound 8e (400 MHz, DMSO-*d6*)

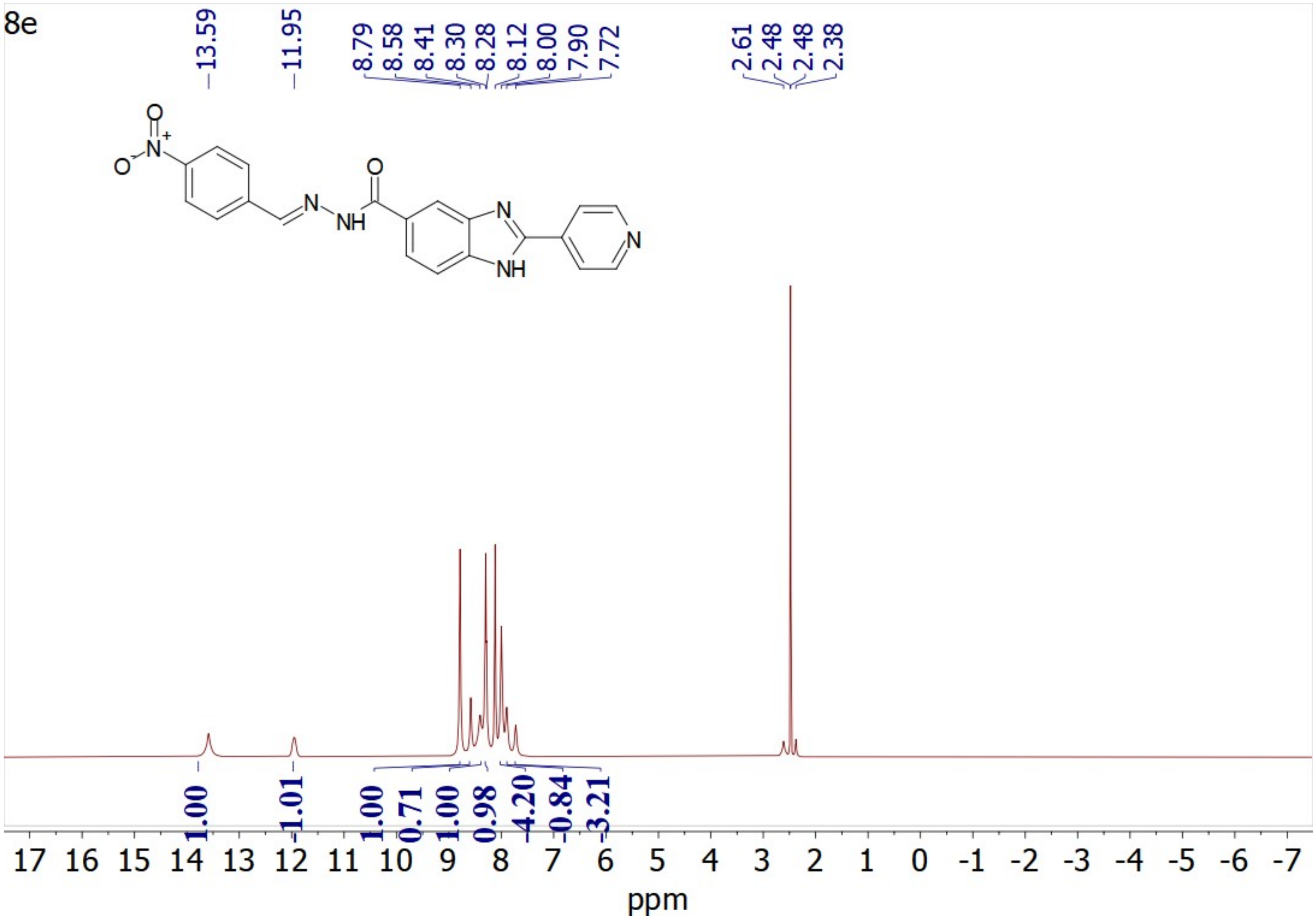

S36: ^{13}C NMR spectrum of compound 8e (100 MHz, DMSO-*d6*)

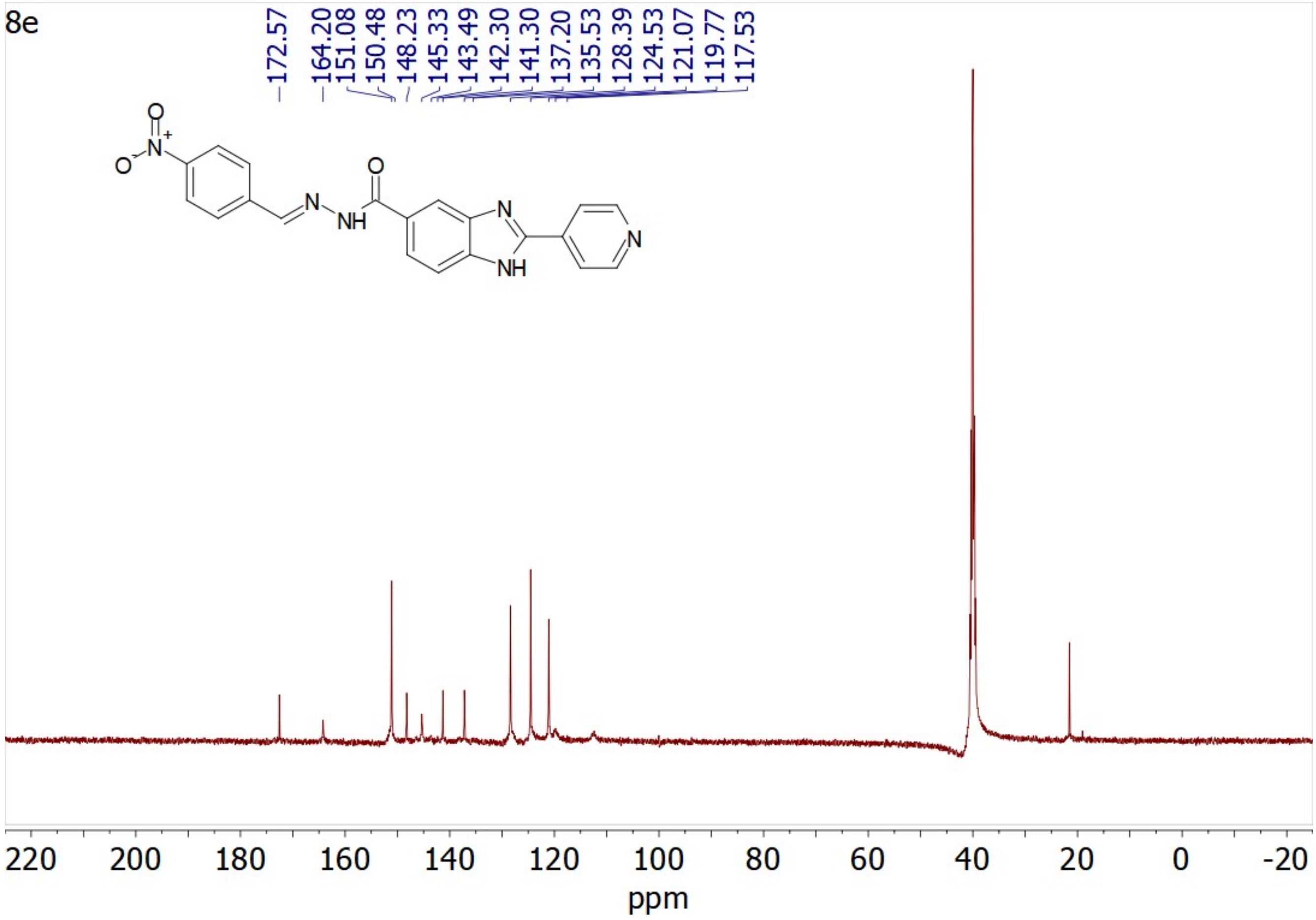

S37: Mass spectrum of compound 8e

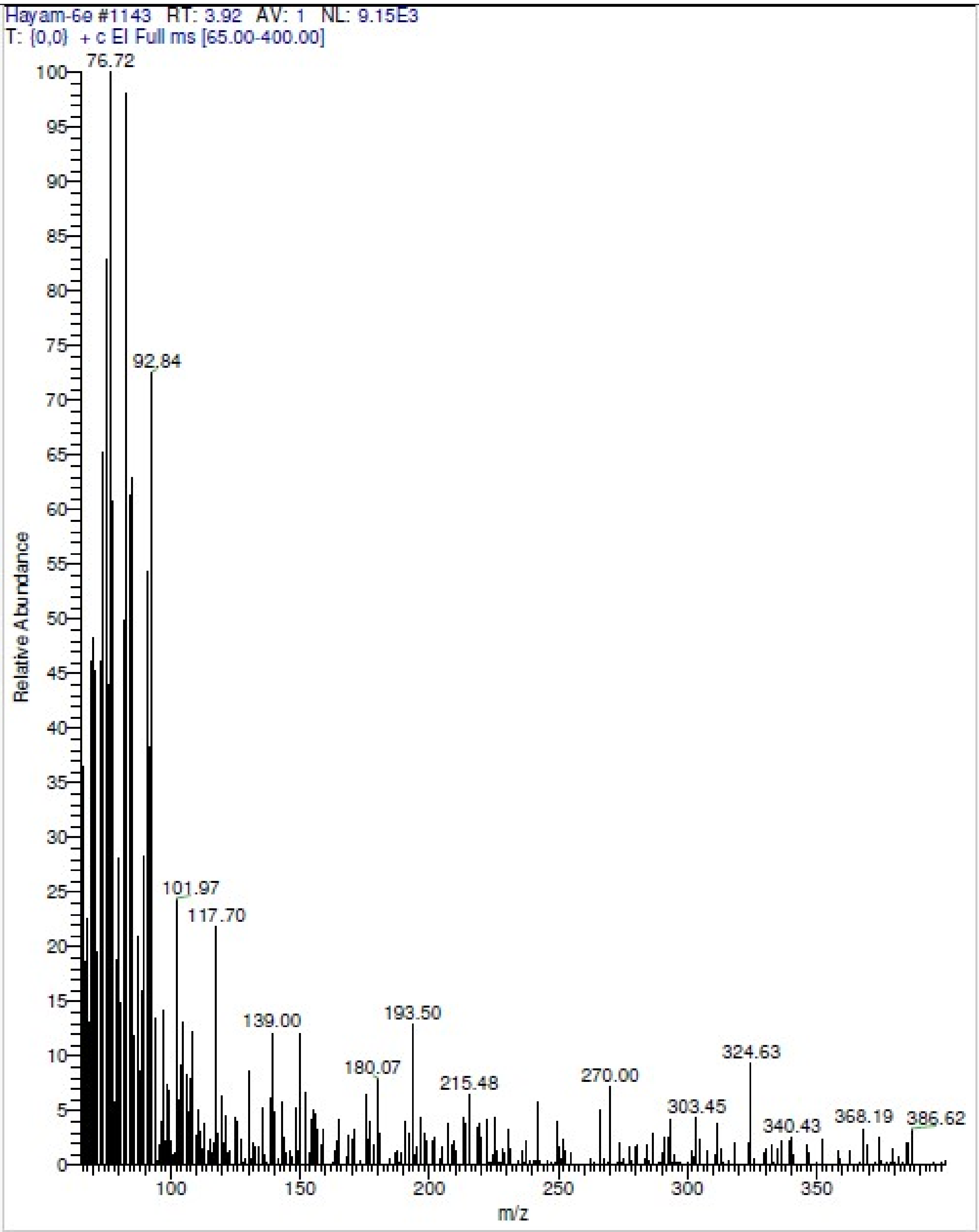

S39: ^{13}C NMR spectrum of compound <u>8f</u> (100 MHz, DMSO-*d6*)

S38: ^{1}H NMR spectrum of compound <u>8f</u> (400 MHz, DMSO-*d6*)

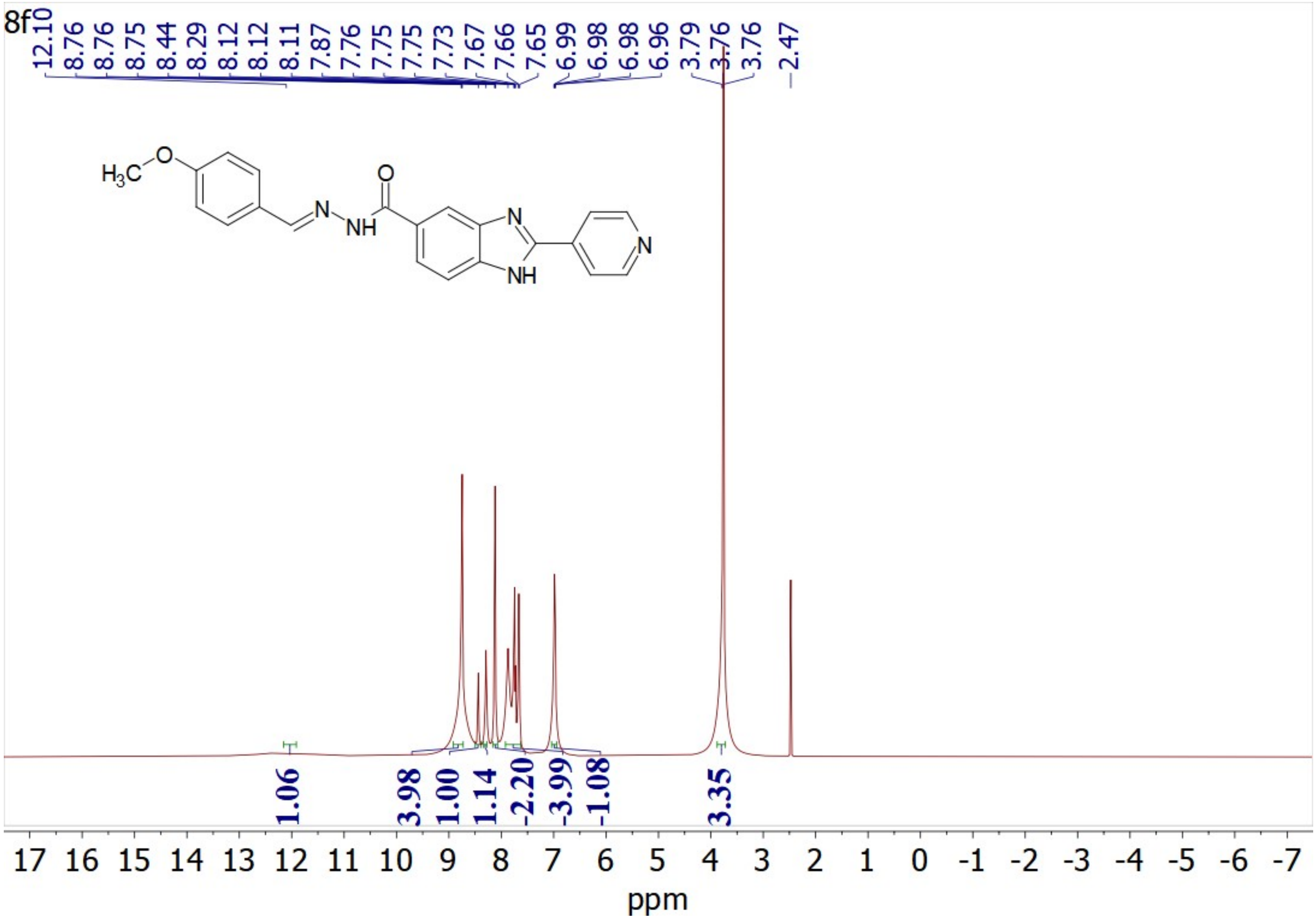

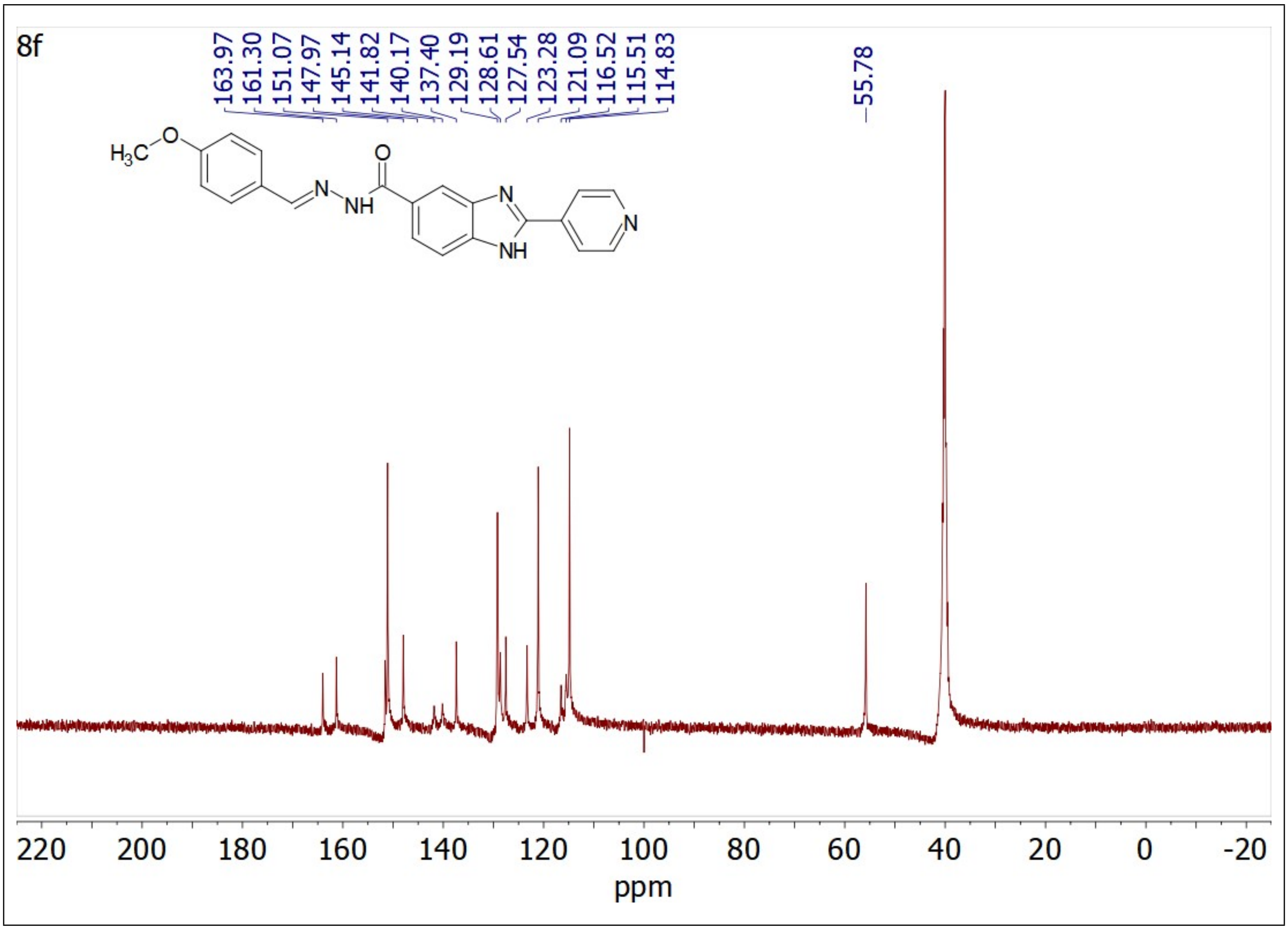
8f
163.97
161.30
151.07
147.97
145.14
141.82
140.17
137.40
129.19
128.61
127.54
123.28
121.09
116.52
115.51
114.83
55.78
H3C
O
N
NH
O
N
NH
N
220 200 180 160 140 120 100 80 60 40 20 0 -20
ppm

S40: Mass spectrum chart of compound 8f

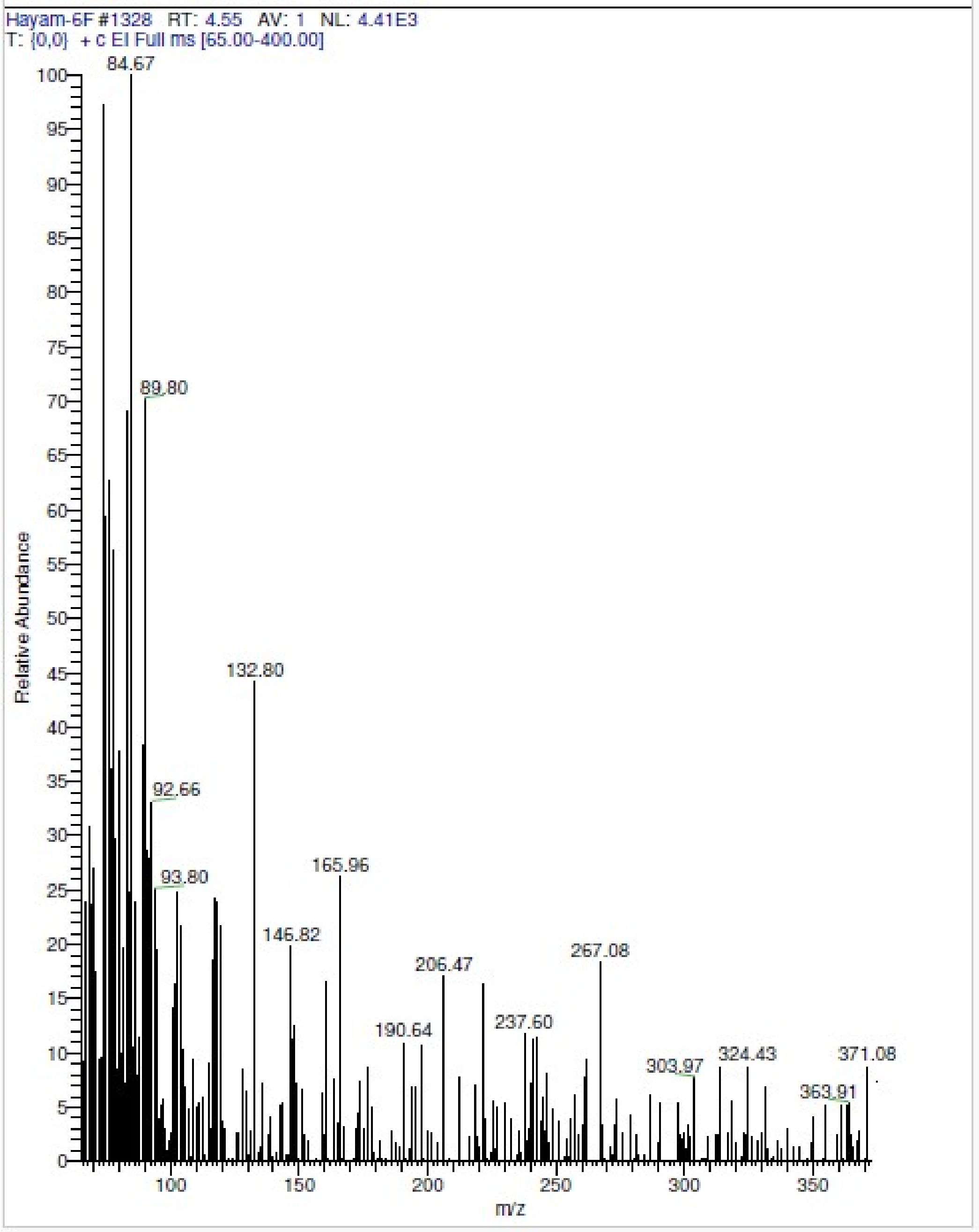

General procedure for synthesis of compound 3a

A solution of o-diaminoaryl derivative (2 mmol), the appropriate substituted benzaldehyde (2 mmol), and sodium metabisulfite $Na_2S_2O_5$, 2.40 mmol) in dimethylformamide was heated under reflux for 6–12 hours. Upon completion of the reaction (as monitored by TLC), the mixture was allowed to cool and subsequently poured into ice-water. The resulting precipitate was collected via filtration and purified by recrystallization from ethanol to afford the target compound 3a in good yields.

2-(thiophen-2-yl)-1H-benzimidazole-5-carboxylic acid (3a)

white powder, 88% yield, m.p. 125-127 ^{o}C, ^{1}HNMR (DMSO-d_6, 400 MHz, δ ppm): 7.23(s, 1H, O**H** carboxylic acid, D_2O exchangeable), 7.25 (d, 1H, *J* = 8.00 Hz, Ar-H), 7.62 (d, 1H, *J* = 8.00 Hz, Ar-H), 7.87 (d, 1H, *J* = 8.00 Hz, Ar-H), 7.82 (d, 1H, *J* = 8.00 Hz, Ar-H), 7.86 (d, 1H, *J* = 8.00 Hz, Ar-H), 8.12 (s, 1H, Ar-H), 13.16 (s, 1H, N**H** benzimidazole, D_2O exchangeable).

General procedure for synthesis of compound 4a

To a stirred solution of the appropriate carboxylic acid 3a (1.77 mmol) in absolute methanol (50 mL), a catalytic amount of concentrated sulfuric acid (a few drops) was added. The resulting mixture was heated under reflux for 17 hours. Upon completion, the reaction mixture was cooled to room temperature and poured into distilled water (50 mL). The solution was then neutralized by the addition of a 5% sodium carbonate (Na_2CO_3) solution. The resulting precipitate was collected by filtration and purified via recrystallization from methanol to afford compounds 4a in good yields.

methyl 2-(thiophen-2-yl)-1H-benzimidazole-5-carboxylate (4a)

Buff powder, 67% yield, m.p. 200-202 ^{o}C, ^{1}HNMR (DMSO-d_6, 400 MHz, δ ppm): 3.84 (s, 3H, C**H**$_3$), 7.24 (m, 1H, Ar-H), 7.61 (d, 1H, *J* = 8.00 Hz, Ar-H), 7.77 (d, 1H, *J* = 8.00 Hz, Ar-H), 7.82 (d, 1H, *J* = 8.00 Hz, Ar-H), 7.86 (d, 1H, *J* = 8.00 Hz, Ar-H), 8.11(s, 1H, Ar-H), 13.32 (s, 1H, N**H** benzimidazole, D_2O exchangeable).

General procedure for synthesis of compound 5a

A mixture of the appropriate ester 4a (1 mmol) and hydrazine monohydrate (95%, 2 mmol, 5 mL) in ethanol (15 mL) was heated under reflux for 7 hours. After cooling to room temperature, the reaction mixture was poured into ice-cold water. The resulting precipitate was collected by filtration, washed thoroughly with water, and dried under vacuum. Purification by recrystallization from absolute ethanol afforded the target compound **5a** in good yields.

2-(thiophen-2-yl)-1*H*-benzimidazole-5-carbohydrazide (5a)

Gray powder, 60% yield, m.p. 216-218 ^{o}C, ^{1}HNMR (DMSO-d_6, 400 MHz, δ ppm): 4.57 (s, 2H, N**H$_2$**, D_2O exchangeable), 6.23(m, 1H, Ar-H), 7.53-7.62 (m, 2H, Ar-H), 7.74 (d, 1H, *J* = 8.00 Hz, Ar-H), 7.87 (d, 1H, *J* = 8.00 Hz, Ar-H), 8.09 (s, 1H, Ar-H), 9.78 (s, 1H, N**H** amide, D_2O exchangeable), 13.17 (brs, 1H, N**H** benzimidazole, D_2O exchangeable).

Average of Relative viability of cells (%)

Conc.(µM)	HePG2	HCT116	MCF7
DOX			
100	6.3	7.1	6.2
50	11.2	13.9	10.9
25	14.1	18.7	14.3
12.5	28.3	31.4	26.9
6.25	45.8	47.9	41.5
3.125	57.6	60.5	58.4
1.56	71.2	73.8	69.1
7d			
100	35.4	36.1	24.7
50	49.1	48.3	39.2
25	56.2	63.4	51.4
12.5	69.9	75.2	68.3
6.25	83.7	96.0	79.1
3.125	98.3	100	97.4
1.56	100	100	100
7a			
100	28.5	25.3	20.5
50	41.7	31.9	29.3
25	53.3	45.6	37.1
12.5	69.6	57.2	47.8
6.25	81.8	70.4	68.7
3.125	100	91.7	87.2
1.56	100	100	100
7e			
100	31.6	31.4	25.6
50	40.9	41.7	36.4
25	61.1	52.8	50.1
12.5	70.3	73.5	64.7
6.25	86.5	88.2	80.5
3.125	99.7	99.3	96.1
1.56	100	100	100
8f			
100	8.6	7.9	8.5
50	16.9	19.3	16.8
25	24.2	22.6	25.7
12.5	32.7	39.2	31.9
6.25	55.8	58.4	60.3
3.125	70.3	85.7	69.6
1.56	88.4	98.1	89.4

7b			
100	23.9	29.1	20.3
50	37.8	38.4	31.1
25	49.3	52.6	42.4
12.5	53.7	63.9	54.2
6.25	70.2	80.3	71.5
3.125	92.5	96.2	92.3
1.56	100	100	100
7f			
100	43.6	47.7	35.6
50	52.3	58.5	48.2
25	62.1	72.4	60.5
12.5	78.5	84.6	72.3
6.25	99.2	98.1	89.4
3.125	100	100	100
1.56	100	100	100
8e			
100	36.4	41.9	30.4
50	49.3	49.1	41.6
25	60.2	65.4	53.1
12.5	74.5	78.3	65.4
6.25	87.1	92.5	79.2
3.125	100	100	98.7
1.56	100	100	100
7c			
100	48.3	55.2	46.5
50	60.5	68.5	57.3
25	76.1	81.6	70.2
12.5	88.2	95.1	83.4
6.25	98.9	100	96.5
3.125	100	100	100
1.56	100	100	100
8b			
100	38.4	49.7	33.6
50	50.9	61.3	45.2
25	62.5	72.9	56.0
12.5	78.7	86.1	69.3
6.25	96.3	99.4	91.1
3.125	100	100	100
1.56	100	100	100
8a			
100	45.9	42.6	39.4
50	56.7	57.3	54.9
25	68.3	69.5	72.1
12.5	84.6	81.2	93.6
6.25	99.2	92.4	100
3.125	100	100	100

**1.56**	**100**	**100**	**100**
**8c**			
**100**	**21.6**	**18.8**	**13.5**
**50**	**28.4**	**26.7**	**22.3**
**25**	**41.7**	**39.5**	**30.4**
**12.5**	**50.3**	**52.6**	**45.1**
**6.25**	**72.5**	**70.4**	**64.7**
**3.125**	**91.2**	**87.3**	**86.2**
**1.56**	**100**	**100**	**98.6**
**8d**			
**100**	**8.3**	**4.7**	**6.5**
**50**	**12.9**	**10.2**	**13.1**
**25**	**21.5**	**16.5**	**20.8**
**12.5**	**30.6**	**23.4**	**33.6**
**6.25**	**54.8**	**41.6**	**48.7**
**3.125**	**62.2**	**58.1**	**63.3**
**1.56**	**75.4**	**67.3**	**86.2**

B-Raf (V600E) Kinase activity assay

Compound 5a
IC_{50} = 198.083 nM

log10(Concentration nM)	% Remaining Activity
3.000	13.88
2.699	24.66
2.398	43.56
2.097	60.92
1.796	77.62
1.495	87.2
1.194	92.91
0.893	97.83

Raw Data

Concentration (nM)	RLU1	RLU2	RLU Average
1000	22449.408	22461.294	22455.351
500	21389.873	21403.235	21396.554
250	19494.269	19506.369	19500.319
125	19484.682	19496.758	19490.72
62.5	17139.053	17148.549	17143.801
31.25	13208.111	13216.615	13212.363
15.625	12800.954	12809.675	12805.315
7.813	12071.476	12083.181	12077.329

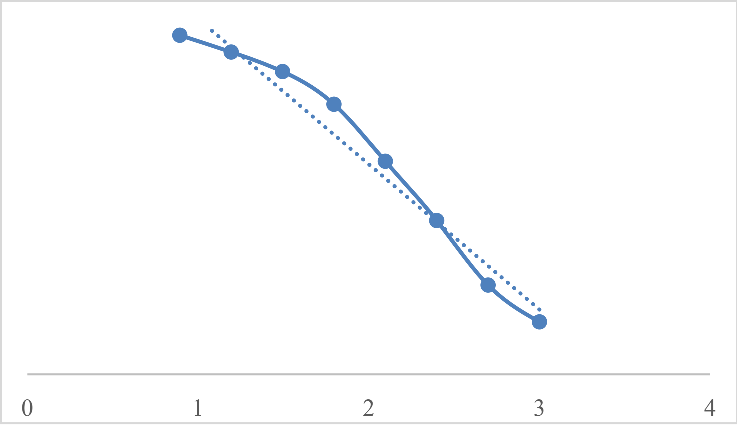

Compound 5b
IC_{50} = 113.246 nM

log10(Concentration nM)	% Remaining Activity
3.000	10.74
2.699	21.2
2.398	30.48
2.097	38.26
1.796	46.98
1.495	59.24
1.194	70.12
0.893	78.15

Raw Data

Concentration (nM)	RLU1	RLU2	RLU Average
1000	20406.304	20415.918	20411.111
500	19180.792	19192.928	19186.86
250	17223.061	17227.154	17225.108
125	16051.475	16060.174	16055.824
62.5	16087.629	16091.601	16089.615
31.25	12885.329	12889.634	12887.482
15.625	10344.167	10353.478	10348.823
7.813	8498.225	8501.464	8499.844

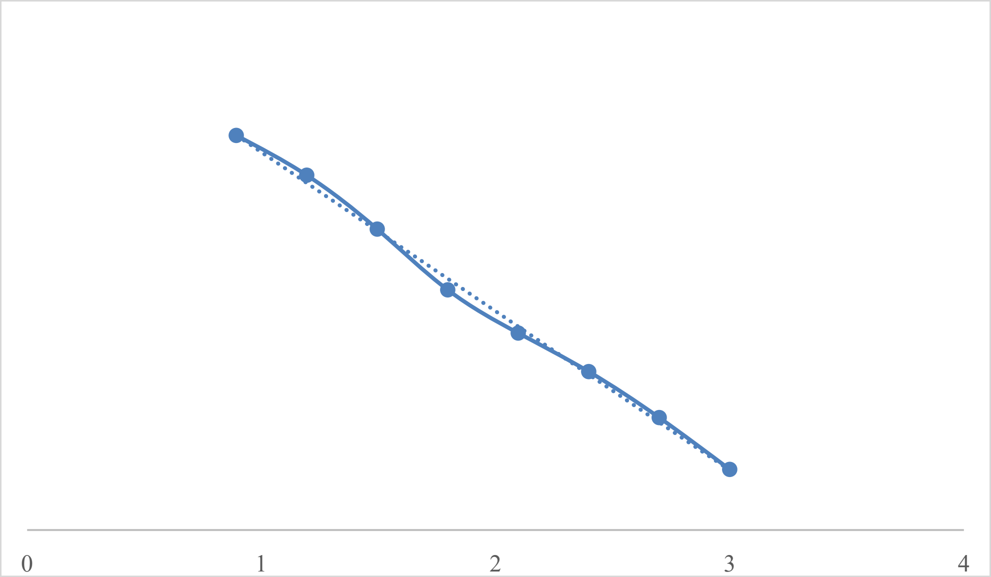

Compound 5e

IC_{50} = 78.107 nM

log10(Concentration nM)	% Remaining Activity
3.000	6.76
2.699	10.13
2.398	21.99
2.097	36.16
1.796	57.15
1.495	71.67
1.194	85.05
0.893	93.58

Raw Data

Concentration (nM)	RLU1	RLU2	RLU Average
1000	23831.335	23842.844	23837.089
500	22131.361	22140.138	22135.749
250	21766.495	21770.147	21768.321
125	20652.475	20655.652	20654.064
62.5	19659.879	19663.745	19661.812
31.25	15799.586	15811.674	15805.63
15.625	14719.145	14725.254	14722.199
7.813	10681.088	10691.66	10686.374

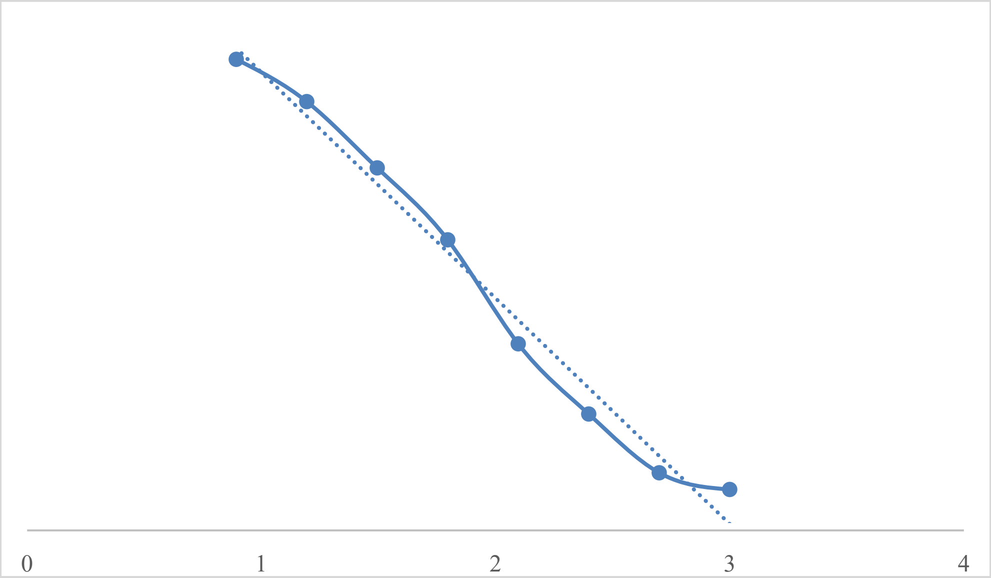

Compound 6c
IC_{50} = 253.532 nM

log10(Concentration nM)	% Remaining Activity
3.000	15.64
2.699	25.25
2.398	30.86
2.097	41.55
1.796	47.5
1.495	55.9
1.194	65.81
0.893	73.21

Raw Data

Concentration (nM)	RLU1	RLU2	RLU Average
1000	25280.754	25292.818	25286.786
500	23768.61	23777.31	23772.96
250	21633.557	21639.194	21636.375
125	19917.353	19925.171	19921.262
62.5	18668.752	18678.816	18673.784
31.25	17726.404	17732.166	17729.285
15.625	14983.993	14989.945	14986.969

Concentration (nM)	RLU1	RLU2	RLU Average
7.813	13101.581	13106.334	13103.958

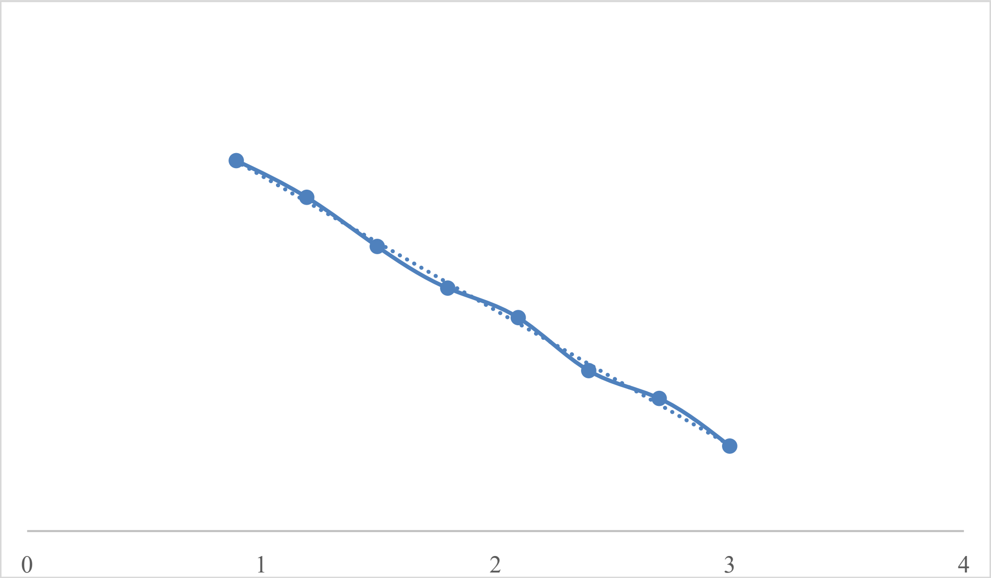

Compound 6d

IC_{50} = 45.471 nM

log10(Concentration nM)	% Remaining Activity
3.000	4.38
2.699	7.12
2.398	13.58
2.097	22.83
1.796	42.75
1.495	60.85
1.194	77.98
0.893	87.89

Raw Data

Concentration (nM)	RLU1	RLU2	RLU Average
1000	24054.751	24057.656	24056.203
500	22580.85	22585.403	22583.127
250	21615.581	21619.454	21617.517
125	18864.656	18869.863	18867.26
62.5	16756.209	16769.798	16763.004

Concentration (nM)	RLU1	RLU2	RLU Average
31.25	17091.597	17099.492	17095.544
15.625	18612.07	18621.715	18616.893
7.813	12642.786	12652.763	12647.774

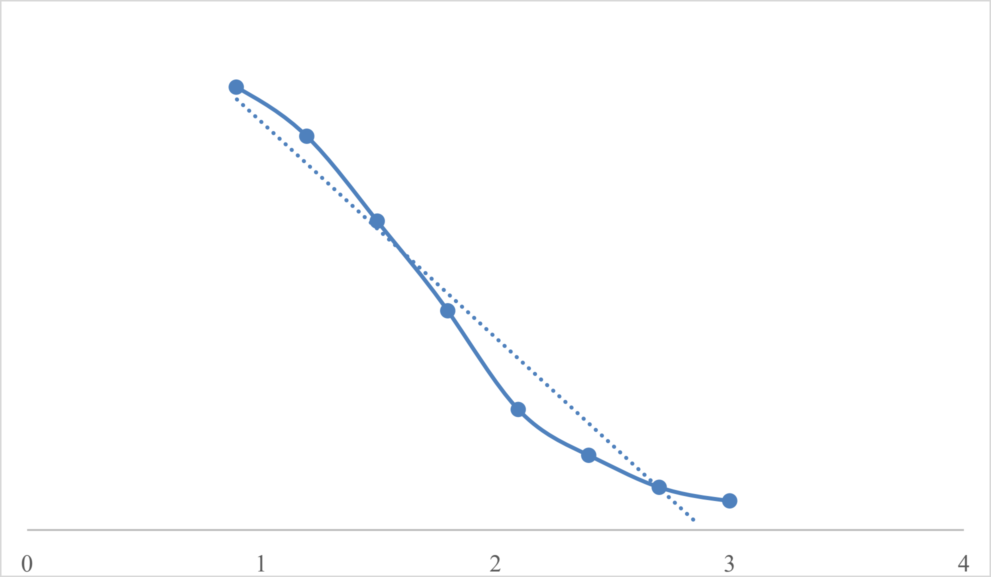

Compound 6f
IC_{50} = 57.694 nM

log10(Concentration nM)	% Remaining Activity
3.000	2.68
2.699	6.69
2.398	18.59
2.097	28.83
1.796	45.87
1.495	64.51
1.194	79.25
0.893	89.45

Raw Data

Concentration (nM)	RLU1	RLU2	RLU Average
1000	21313.106	21321.299	21317.203
500	20017.924	20020.409	20019.166
250	18392.321	18402.959	18397.64

Concentration (nM)	RLU1	RLU2	RLU Average
125	18032.245	18041.065	18036.655
62.5	16960.387	16962.632	16961.509
31.25	16641.369	16650.795	16646.082
15.625	12085.157	12088.058	12086.607
7.813	12936.929	12949.085	12943.007

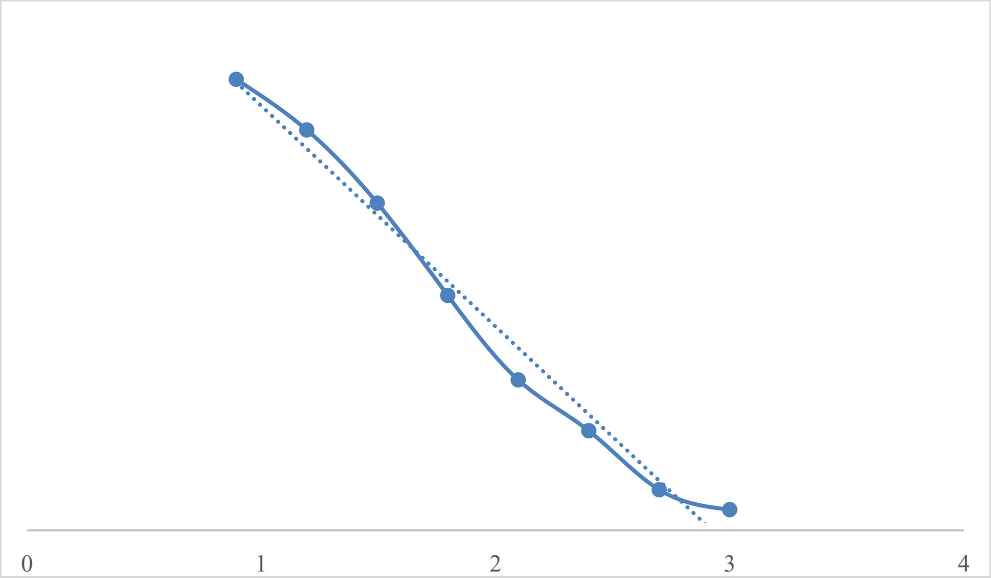

Compound Vemurafenib
IC_{50} = 41.382 nM

log10(Concentration nM)	% Remaining Activity
3.000	3.08
2.699	5.03
2.398	12.55
2.097	23.5
1.796	38.01
1.495	58.58
1.194	73.7
0.893	84.51

Raw Data

Concentration (nM)	RLU1	RLU2	RLU Average
1000	20569.414	20571.795	20570.604

Concentration (nM)	RLU1	RLU2	RLU Average
500	18774.105	18780.335	18777.22
250	16840.236	16847.761	16843.999
125	15977.821	15989.711	15983.766
62.5	16891.102	16895.663	16893.383
31.25	14712.597	14717.96	14715.278
15.625	12275.764	12278.795	12277.279
7.813	8648.497	8655.406	8651.951

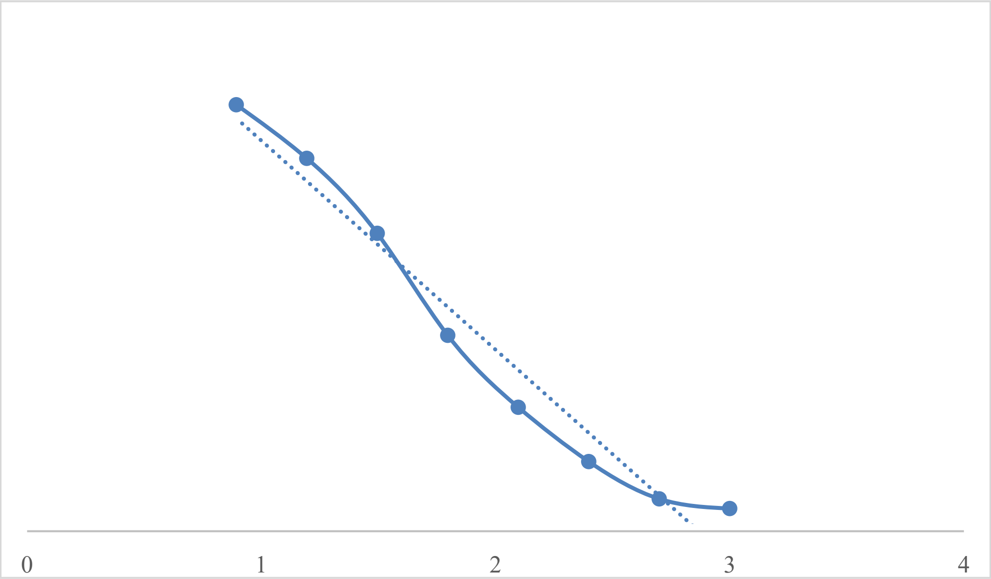

EGFR Kinase activity assay

Compound 6c
IC$_{50}$ = 22.904 nM

Log Concentration (nM)	% Remaining Activity
1.699	23.21
1.398	41.87
1.097	64.92
0.796	75.84
0.495	84.13
0.194	91.62
-0.107	95.48

Raw Data

Concentration (nM)	RLU1	RLU2	RLU Average
50.000	14823.462	14796.118	14809.790
25.000	18291.337	18318.904	18305.121
12.500	21987.554	21942.119	21964.836
6.250	25648.771	25611.309	25630.040
3.125	28792.448	28821.903	28807.176
1.563	31488.204	31455.736	31471.970
0.781	33291.661	33324.190	33307.926

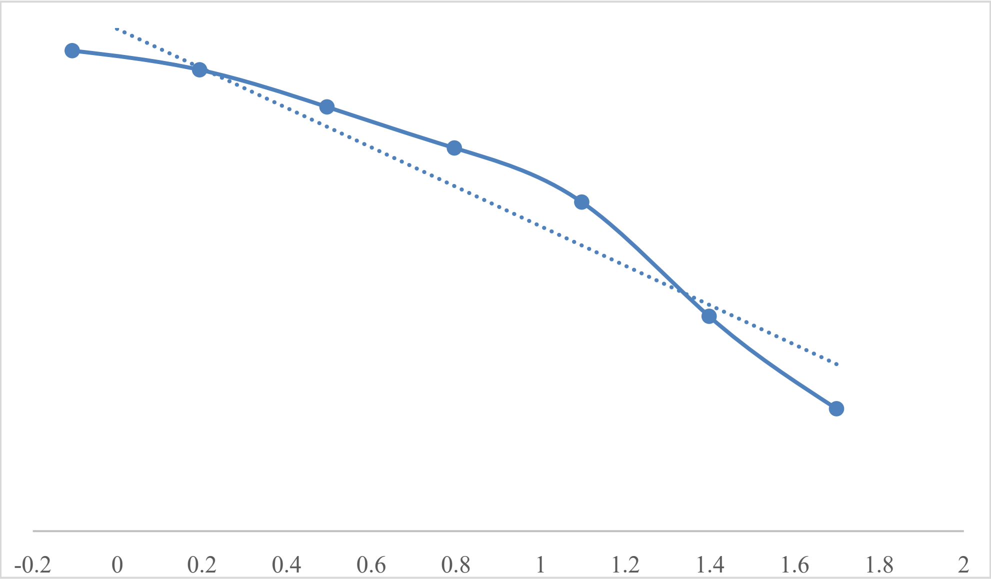

Compound 6d
IC_{50} = 7.175 nM

Log Concentration (nM)	% Remaining Activity
1.699	32.11
1.398	41.67
1.097	54.83
0.796	69.92
0.495	83.76
0.194	92.28
-0.107	97.01

Raw Data

Concentration (nM)	RLU1	RLU2	RLU Average
50.000	10124.739	10157.382	10141.061
25.000	13694.551	13621.984	13658.268
12.500	17942.116	18003.748	17972.932
6.250	22984.503	22891.447	22937.975
3.125	27416.338	27521.774	27469.056
1.563	31684.990	31593.214	31639.102
0.781	34622.731	34541.893	34582.312

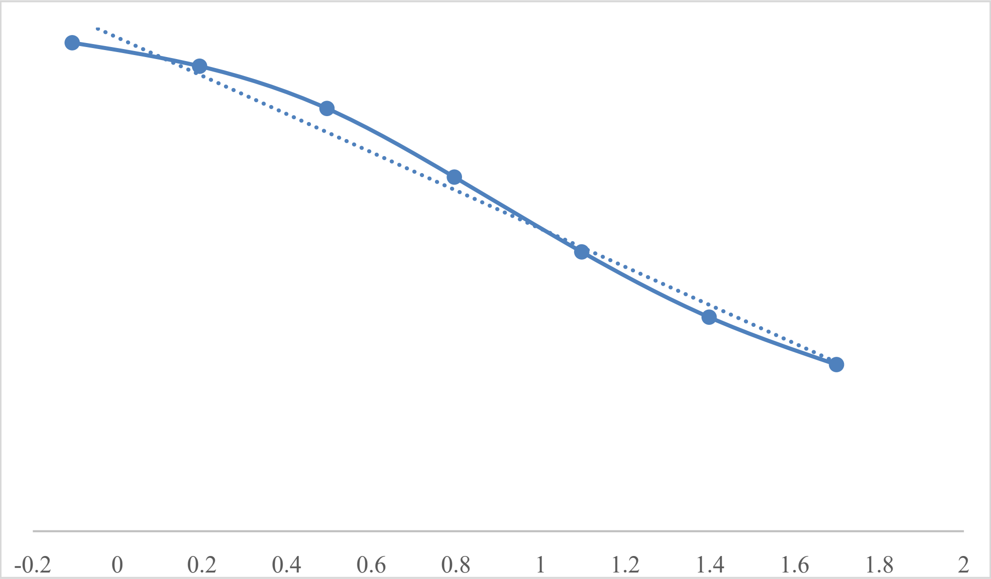

Compound 6f

IC_{50} = 11.483 nM

Log Concentration (nM)	% Remaining Activity
1.699	28.94
1.398	39.72
1.097	52.88
0.796	67.93
0.495	81.46
0.194	90.17
-0.107	96.08

Raw Data

Concentration (nM)	RLU1	RLU2	RLU Average
50.000	8421.774	8463.291	8442.533
25.000	11894.563	11842.119	11868.341
12.500	16273.908	16344.517	16309.213
6.250	21482.664	21413.552	21448.108
3.125	26891.406	26962.771	26927.089
1.563	31974.285	31892.416	31933.351
0.781	35641.907	35722.364	35682.136

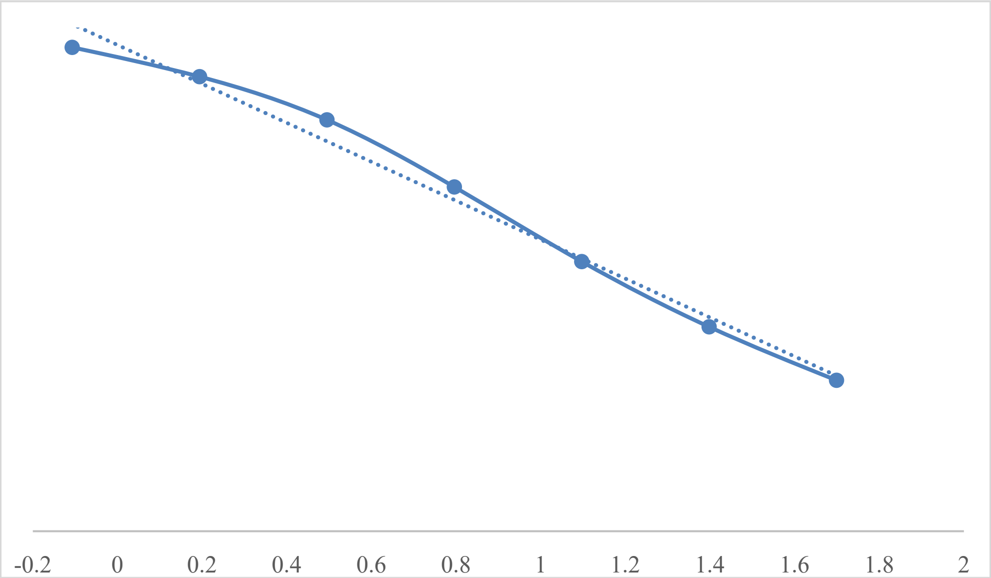

Compound 5a
IC_{50} = 56.772 nM

Log Concentration (nM)	% Remaining Activity
1.699	44.43
1.398	56.88
1.097	71.96
0.796	85.94
0.495	89.91
0.194	93.62
-0.107	97.44

Raw Data

Concentration (nM)	RLU1	RLU2	RLU Average
50.000	25114.773	25068.442	25091.608
25.000	26983.116	27041.552	27012.334
12.500	28762.904	28691.337	28727.121
6.250	30194.447	30241.993	30218.220
3.125	31642.558	31588.224	31615.391
1.563	32941.772	33012.489	32977.131
0.781	34288.631	34231.904	34260.268

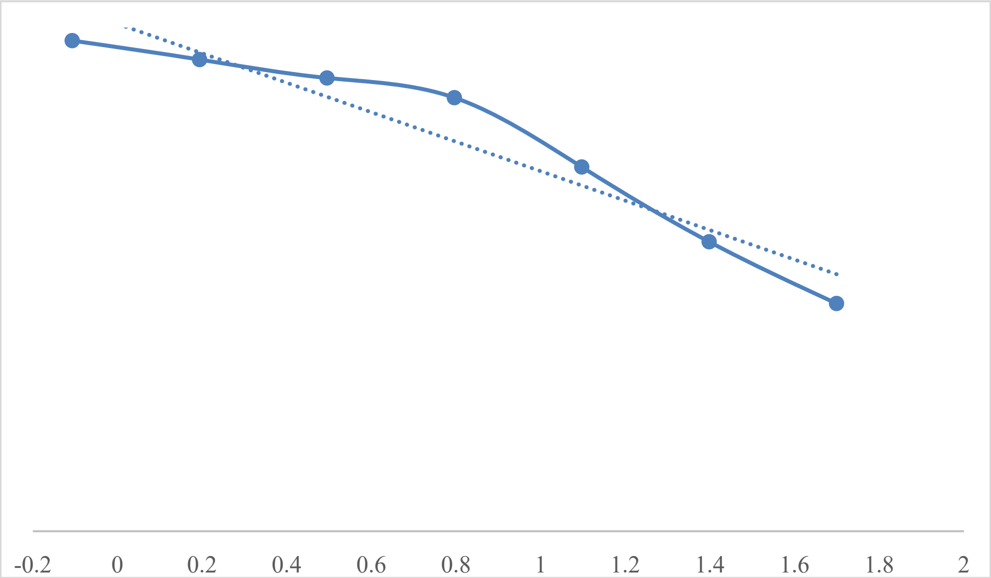

Compound 5b

IC_{50} = 53.259 nM

Log Concentration (nM)	% Remaining Activity
1.699	53.12
1.398	57.84
1.097	62.61
0.796	76.73
0.495	80.68
0.194	94.31
-0.107	97.89

Raw Data

Concentration (nM)	RLU1	RLU2	RLU Average
50.000	26391.482	26441.773	26416.628
25.000	27984.631	27921.294	27952.963
12.500	29641.904	29712.558	29677.231
6.250	31294.337	31352.119	31323.228
3.125	32841.775	32791.462	32816.619
1.563	34112.663	34171.228	34141.946
0.781	35491.904	35421.337	35456.621

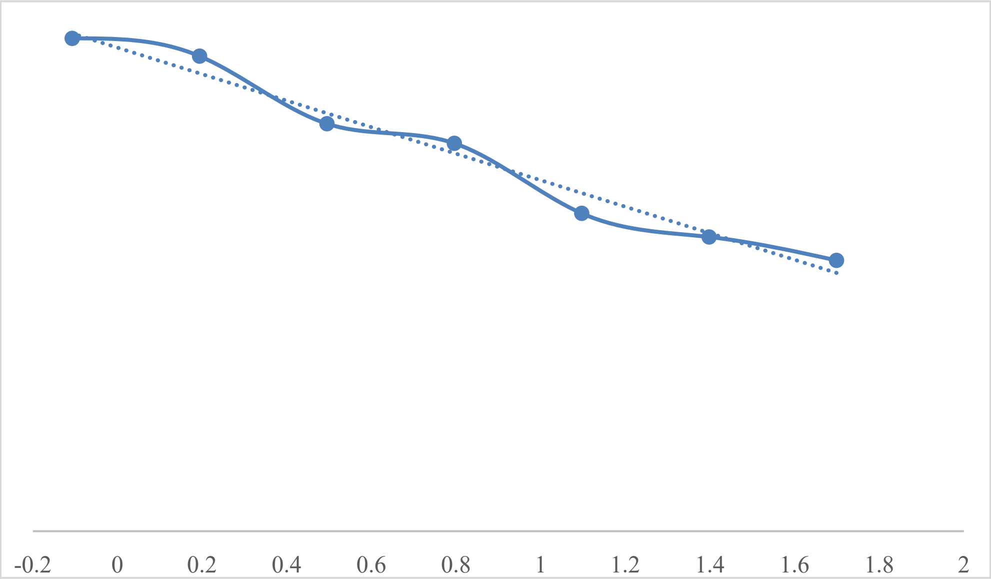

Compound 5e
IC_{50} = 39.861 nM

Log Concentration (nM)	% Remaining Activity
1.699	31.84
1.398	57.91
1.097	64.88
0.796	81.46
0.495	87.93
0.194	92.64
-0.107	97.12

Raw Data

Concentration (nM)	RLU1	RLU2	RLU Average
50.000	21492.773	21541.338	21517.056
25.000	23741.904	23688.229	23715.067
12.500	25984.116	26051.447	26017.782
6.250	28341.662	28271.993	28306.828
3.125	30612.489	30684.771	30648.630
1.563	32794.338	32741.906	32768.122
0.781	34921.774	34852.119	34886.947

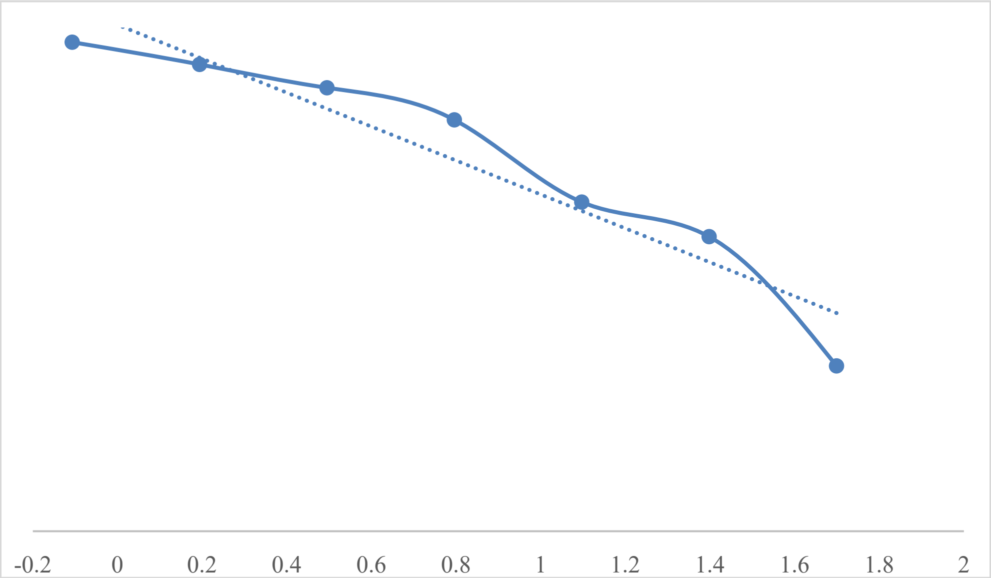

Compound Erlotinib
IC_{50} = 5.392 nM

Log Concentration (nM)	% Remaining Activity
1.699	18.94
1.398	26.73
1.097	38.61
0.796	52.44
0.495	68.92
0.194	83.71
-0.107	92.84

Raw Data

Concentration (nM)	RLU1	RLU2	RLU Average
50.000	6421.884	6462.337	6442.111
25.000	8241.663	8196.294	8218.979
12.500	11094.772	11141.906	11118.339
6.250	15182.441	15241.993	15212.217
3.125	20491.338	20542.774	20517.056
1.563	26894.229	26941.884	26918.057
0.781	31841.906	31794.552	31818.229

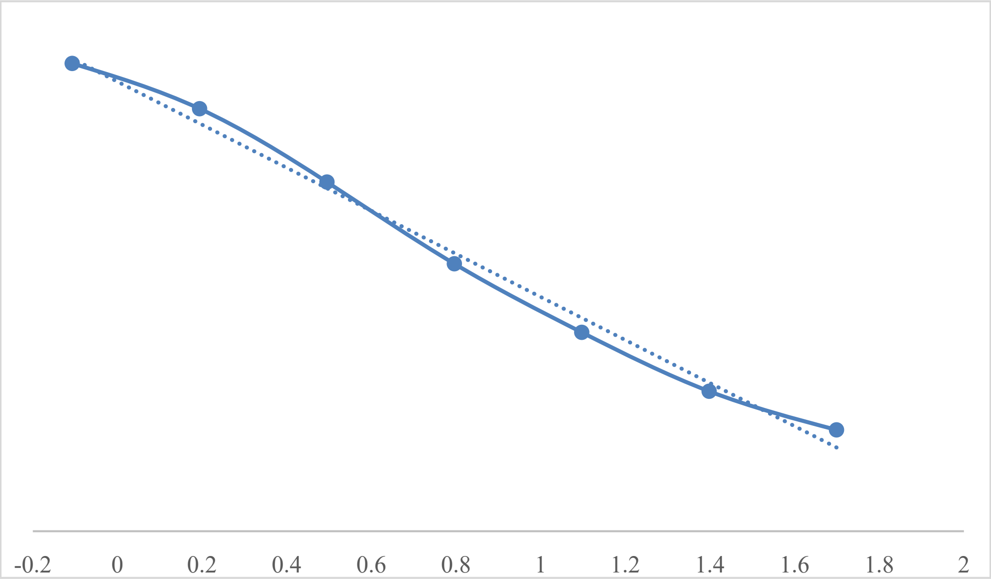

ADMET of Compound 8d

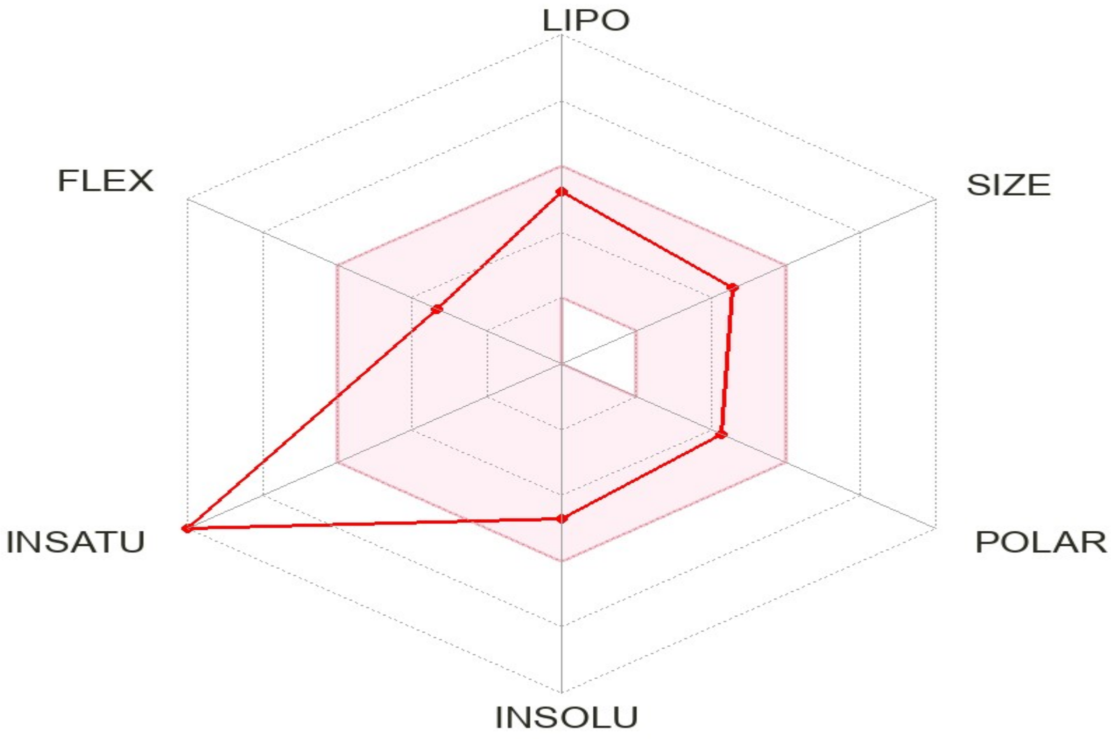

Physicochemical Properties

Formula	C20H14ClN5O
Molecular weight	375.81 g/mol

Num. heavy atoms	27
Num. arom. heavy atoms	21
Fraction Csp3	0.00
Num. rotatable bonds	5
Num. H-bond acceptors	4
Num. H-bond donors	2
Molar Refractivity	105.50
TPSA	83.03 Å^2

Lipophilicity

Log $P_{o/w}$ (iLOGP)	2.12
Log $P_{o/w}$ (XLOGP3)	3.62
Log $P_{o/w}$ (WLOGP)	4.04
Log $P_{o/w}$ (MLOGP)	2.76
Log $P_{o/w}$ (SILICOS-IT)	4.62
Consensus Log $P_{o/w}$	3.43

Water Solubility

Log *S* (ESOL)	-4.70
Solubility	7.56e-03 mg/ml ; 2.01e-05 mol/l
Class	Moderately soluble
Log *S* (Ali)	-5.05
Solubility	3.34e-03 mg/ml ; 8.89e-06 mol/l

Class	Moderately soluble
Log *S* (SILICOS-IT)	-8.22
Solubility	2.24e-06 mg/ml ; 5.97e-09 mol/l
Class	Poorly soluble

Pharmacokinetics

GI absorption	High
BBB permeant	No
P-gp substrate	No
CYP1A2 inhibitor	Yes
CYP2C19 inhibitor	Yes
CYP2C9 inhibitor	Yes
CYP2D6 inhibitor	Yes
CYP3A4 inhibitor	No
Log K_p (skin permeation)	-6.02 cm/s

Drug Likness

Lipinski	Yes; 0 violation
Ghose	Yes
Veber	Yes

Egan	Yes
Muegge	Yes
Bioavailability Score	0.55

Medicinal Chemistry

PAINS	0 alert
Brenk	1 alert: imine_1
Lead likeness	No; 2 violations: MW>350, XLOGP3>3.5
Synthetic accessibility	2.76

Appendix A

4. EXPERIMENTAL

4.1. Chemistry

Materials and methods

All reagents and solvents were of general purpose or analytical grade and purchased from Sigma Aldrich Ltd, Fisher Scientific, Fluka and Acros. ^1H and ^{13}C NMR spectra were recorded with a Bruker Avance III spectrometer operating at 400, 100 MHz respectively, with Me4Si as internal standard and DMSO-d6 as a solvent. Elemental analysis was performed by the regional center for mycology and biotechnology (Cairo, Egypt). TLC was carried out on precoated silica plates (Keisel gel 60 F254, BDH) using Hexane: Ethyl acetate, 1 : 2, v/v. Compounds were visualized by illumination under UV light (254 nm). Melting points were determined on an electrothermal instrument and are uncorrected. All solvents were dried prior to use and stored over 4 Å molecular sieves, under nitrogen. All the compounds were ≥ 95% pure.

4.2. Biological evaluation

4.2.1 Cell Viability assay (MTT assay)

MTT assay was performed to investigate the effect of the synthesized compounds on mammary epithelial cells (MCF-10A). The cells were propagated in medium consisting of Ham's F-12 medium/ Dulbecco's modified Eagle's medium (DMEM) (1:1) supplemented with 10% foetal calf serum (GIBCO, UK), 2 mM glutamine, insulin (10 μg/mL), hydrocortisone (500 ng/mL) and epidermal growth factor (20 ng/mL). Trypsin ethylenediamine tetra acetic acid (EDTA) was used to passage the cells after every 2-3 days. 96-well flat-bottomed cell culture plates were used to seed the cells at a density of 104 cells mL^{-1}. The medium was aspirated from all the wells of culture plates after 24 h followed by the addition of synthesized compounds (in 200 μL medium to yield a final concentration of 0.1% (v/v) dimethyl sulfoxide) into individual wells of the plates. Four wells were designated to a single compound. The plates were allowed to incubate at 37°C for 96 h. Afterwards, the medium was aspirated and 3-[4,5-dimethylthiazol-2-yl]-2,5-diphenyltetrazolium bromide (MTT) (0.4 mg/mL) in medium was added to each well and subsequently incubated for 3 h. The medium was aspirated and 150 μL dimethyl sulfoxide (DMSO) was added to each well. The plates were vortexed followed by the measurement of absorbance at 540 nm on a microplate reader. The results were presented as inhibition (%) of proliferation in contrast to controls comprising 0.1% DMSO.

4.2.2. Assay for antiproliferative effect

To explore the antiproliferative potential of compounds propidium iodide fluorescence assay was performed using different cell lines such as Panc-1 (pancreas cancer cell line) [Panc-1-CTC (RRID:CVCL_VQ69)], MCF-7 (breast cancer cell line) [MCF-7 (RRID:CVCL_0031)], HT-29 (colon cancer cell line) [HT-29 (RRID:CVCL_0320)] and A-549 (epithelial cancer cell line)[A-549 (RRID:CVCL_0023)], respectively. All cell lines were obtained from ATCC (American Type Cell Culture).

To calculate the total nuclear DNA, a fluorescent dye (propidium iodide, PI) is used which can attach to the DNA, thus offering a quick and precise technique. PI cannot pass through the cell membrane and its signal intensity can be considered as directly proportional to quantity of cellular DNA. Cells whose cell membranes are damaged or have changed permeability are counted as dead ones. The assay was performed by seeding the cells of different cell lines at a density of 3000-7500 cells/well (in 200 µl medium) in culture plates followed by incubation for 24 h at 37 °C in humidified 5% CO2 /95% air atmospheric conditions. The medium was removed; the compounds were added to the plates at 10 µM concentrations (in 0.1% DMSO) in triplicates, followed by incubation for 48 h. DMSO (0.1%) was used as control. After incubation, medium was removed followed by the addition of PI (25 µl, 50 µg/mL in water/medium) to each well of the plates. At -80 °C, the plates were allowed to freeze for 24 h, followed by thawing at 25 ^{o}C. A fluorometer (Polar-Star BMG Tech) was used to record the readings at excitation and emission wavelengths of 530 and 620 nm for each well. The percentage cytotoxicity of compounds was calculated using the following formula:

$$\%\ \text{Cytotoxicity} = \frac{A_C - A_{TC}}{A_C} \times 100$$

Where A*TC*= Absorbance of treated cells and AC= Absorbance of control. Erlotinib was used as positive control in the assay.

4.2.3. EGFR inhibitory assay

Baculoviral expression vectors including pBlueBacHis2B and pFASTBacHTc were used separately to clone 1.6 kb cDNA coding for EGFR cytoplasmic domain (EGFR-CD, amino acids 645–1186). 5′ upstream to the EGFR sequence comprised a sequence that encoded (His)6. Sf-9 cells were infected for 72h for protein expression. The pellets of Sf-9 cells were solubilized in a buffer containing sodium vanadate (100 µM),

aprotinin (10 μg/mL), triton (1%), HEPES buffer(50mM), ammonium molybdate (10 μM), benzamidine HCl (16 μg/mL), NaCl (10 mM),leupeptin (10 μg/mL) and pepstatin (10 μg/mL) at 0°C for 20 min at pH 7.4, followed by centrifugation for 20 min. To eliminate the nonspecifically bound material, a Ni-NTA super flow packed column was used to pass through and wash the crude extract supernatant first with 10mM and then with 100 mM imidazole. Histidine-linked proteins were first eluted with 250 and then with 500 mM imidazole subsequent to dialysis against NaCl (50 mM), HEPES (20 mM), glycerol (10%) and 1 μg/mL each of aprotinin, leupeptin and pepstatin for 120 min. The purification was performed either at 4 °C or on ice. To record autophosphorylation level, EGFR kinase assay was carried out on the basis of DELFIA/Time-Resolved Fluorometry. The compounds were first dissolved in DMSO absolute, subsequent to dilution to appropriate concentration using HEPES (25 mM) at pH 7.4. Each compound (10 μL) was incubated with recombinant enzyme (10 μL, 5 ng for EGFR, 1:80 dilution in 100 mM HEPES) for 10 min at 25°C, subsequent to the addition of 5X buffer (10 μL, containing 2 mM MnCl2, 100 μM Na3VO4, 20 mM HEPES and 1 mM DTT) and ATP-MgCl2 (20 μL, containing 0.1 mM ATP and 50 mM MgCl2) and incubation for 1h. The negative and positive controls were included in each plate by the incubation of enzyme either with or without ATP-MgCl2. The liquid was removed after incubation, and the plates were washed thrice using a wash buffer. The Europium-tagged antiphosphotyrosine antibody (75 μL, 400 ng) was added to each well followed by incubation of 1h and then washing of the plates using buffer. The enhancement solution was added to each well and the signal was recorded at excitation and emission wavelengths of 340 at 615 nm. The autophosphorylation percentage inhibition by compounds was calculated using the following equation:

$$100\% - [(negative\ control)/(positive\ control) - (negative\ control)]$$

Using the curves of percentage inhibition of eight concentrations of each compound, IC50 was calculated. The majority of signals detected by antiphosphotyrosine antibody were from EGFR because the enzyme preparation contained low impurities.

4.2.4. BRAF^{V600E} inhibitory assay

V^{600E} mutant BRAF kinase assay was performed to investigate the activity of tested compounds against BRAF. Mouse full-length GST-tagged BRAF^{V600E} (7.5 ng, Invitrogen, PV3849) was pre-incubated with drug (1 µL) and assay dilution buffer (4 µL) for 60 min at 25°C. In assay dilution buffer, a solution (5 µL) containing $MgCl_2$ (30 mM), ATP (200 µM), recombinant human full length (200 ng) and *N*-terminal His-tagged MEK1 (Invitrogen) was added to start the assay, subsequent to incubation for 25 min at 25°C. The assay was stopped using 5X protein denaturing buffer (LDS) solution (5 µL). To further denature the protein, heat (70° C) was applied for 5 min. 4-12% precast Nu-Page gel plates (Invitrogen) were used to carry out electrophoresis (at 200 V). 10 µL of each reaction was loaded into the precast plates and electrophoresis was allowed to proceed. After completion of electrophoresis, the front part of the precast gel plate (holding hot ATP) was cut and afterwards cast-off. The dried gel was developed using a phosphor screen. A reaction without active enzyme was used as negative control while that containing no inhibitor served as positive control. To study the effect of compounds on cell-based pERK1/2 activity in cancer cells, commercially available ELISA kits (Invitrogen) were used according to manufacturer's instructions.

4.2.5. Caspase-3 activation assay

Allow all reagents to reach room temperature before use. Gently mix all liquid reagents prior to use. Determine the number of 8-well strips needed for the assay. Insert these in

the frame(s) for current use. Add 100 µl of the *Standard Diluent Buffer* to the zero standard wells. Well(s) reserved for chromogen blank should be left empty. Add 100 µl of standards and controls or diluted samples to the appropriate microtiter wells. The sample dilution chosen should be optimized for each experimental system. Tap gently on side of plate to mix. Cover wells with *plate cover* and incubate for 2 hours at room temperature. Thoroughly aspirate or decant solution from wells and discard the liquid, Wash wells 4 times. Pipette 100 µl of *Caspase-3 (Active) Detection Antibod*y solution into each well except the chromogen blank(s). Tap gently on the side of the plate to mix. Cover plate with *plate cover* and incubate for 1 hour at room temperature. Thoroughly aspirate or decant solution from wells and discard the liquid, Wash wells 4 times. Add 100 µl Anti-Rabbit IgG HRP Working Solution to each well except the chromogen blank(s). Prepare the working dilution as described in Preparing IgG HRP. Cover wells with the *plate cover* and incubate for 30 minutes at room temperature. Thoroughly aspirate or decant solution from wells and discard the liquid. Wash wells 4 times. Add 100 µl of *Stabilized Chromogen* to each well. The liquid in the wells will begin to turn blue. Incubate for 30 minutes at room temperature and in the dark. The incubation time for chromogen substrate is often determined by the microtiter plate reader used. Many plate readers have the capacity to record a maximum optical density (O.D.) of 2.0. The O.D. values should be monitored, and the substrate reaction stopped before the O.D. of the positive wells exceeds the limits of the instrument. The O.D. values at 450 nm can only be read after the *Stop Solution* has been added to each well. If using a reader that records only to 2.0 O.D., stopping the assay after 20 to 25 minutes is suggested. Add 100 µl of *Stop Solution* to each well. Tap side of plate gently to mix. The solution in the wells should change from blue to yellow. Read the absorbance of each well at 450 nm having blanked the plate reader against a chromogen blank

composed of 100 µl each of *Stabilized Chromogen* and *Stop Solution*. Read the plate within 2 hours after adding the *Stop Solution*. Use a curve fitting software to generate the standard curve. A four-parameter algorithm provides the best standard curve fit. Read the concentrations for unknown samples and controls from the standard curve. Multiply value(s) obtained for sample(s) by the appropriate dilution factor to correct for the dilution in step 3. Samples producing signals greater than that of the highest standard should be diluted in *Standard Diluent Buffer* and reanalyzed.

4.2.6. Caspase-8/9 activation assay

Cells were obtained from American Type Culture Collection, cells were grown in RPMI 1640 containing 10% fetal bovine serum at 37°C, stimulated with the compounds to be tested for caspase 8/9, and lysed with Cell Extraction Buffer. This lysate was diluted in Standard Diluent Buffer over the range of the assay and measured for human active caspase-8/9 content. (*Cells are Plated in a density of 1.2 – 1.8 × 10,000 cells/well in a volume of 100µl complete growth medium + 100 ul of the tested compound per well in a 96-well plate for 24 hours before the enzyme assay*). The absorbance of each microwell was read on a spectro-photometer at 450 nm. A standard curve is prepared from 7 human Caspase-8/9 standard dilutions and human Caspase-8/9 concentration determined.

4.2.7. Bax activation assay

Bring all reagents, except the human Bax-α Standard, to room temperature for at least 30 minutes prior to opening. The human Bax-α Standard solution should not be left at room temperature for more than 10 minutes. All standards, controls and samples should be run in duplicate. Refer to the Assay Layout Sheet to determine the number of wells to be used and put any remaining wells with the desiccant back into the pouch and seal the ziploc. Store unused wells at 4 °C. Pipet 100 µL of Assay Buffer into the S0 (0

pg/mL standard) wells. Pipet 100 µL of Standards #1 through #6 into the appropriate wells. Pipet 100 µL of the Samples into the appropriate wells. Tap the plate gently to mix the contents. Seal the plate and incubate at room temperature on a plate shaker for 1 hour at ~500 rpm. Empty the contents of the wells and wash by adding 400 µL of wash solution to every well. Repeat the wash 4 more times for a total of **5 washes**. After the final wash, empty or aspirate the wells and firmly tap the plate on a lint free paper towel to remove any remaining wash buffer. Pipet 100 µL of yellow Antibody into each well, except the Blank. Seal the plate and incubate at room temperature on a plate shaker for 1 hour at ~500 rpm. Empty the contents of the wells and wash by adding 400 µL of wash solution to every well. Repeat the wash 4 more times for a total of **5** washes. After the final wash, empty or aspirate the wells and firmly tap the plate on a lint free paper towel to remove any remaining wash buffer. Add 100 µL of blue Conjugate to each well, except the Blank. Seal the plate and incubate at room temperature on a plate shaker for 30 minutes at ~500 rpm. Empty the contents of the wells and wash by adding 400 µL of wash solution to every well. Repeat the wash 4 more times for a total of **5 washes**. After the final wash, empty or aspirate the wells and firmly tap the plate on a lint free paper towel to remove any remaining wash buffer. Pipet 100 µL of Substrate Solution into each well. Incubate for 30 minutes at room temperature on a plate shaker at ~500 rpm. Pipet 100 µL Stop Solution to each well. Blank the plate reader against the Blank wells, read the optical density at 450 nm. Calculate the average net Optical Density (OD) bound for each standard and sample by subtracting the average Blank OD from the average OD for each standard and sample. Using linear graph paper, plot the Average Net OD for each standard versus Bax concentration in each standard. Approximate a straight line through the points. The concentration of Bax in the unknowns can be determined by interpolation.

4.2.8. Bcl-2 inhibition assay

Mix all the reagents thoroughly without foaming before use. Wash the microwells twice with approximately 300 µL Wash Buffer per well with thorough aspiration of microwell contents between washes. Take caution not to scratch the surface of the microwells. After the last wash, empty the wells and tap microwell strips on absorbent pad or paper towel to remove excess Wash Buffer. Use the microwell strips immediately after washing or place upside down on a wet absorbent paper for not longer than 15 minutes. Do not allow wells to dry. Add 100 µL of Sample Diluent in duplicate to all standard wells and to the blank wells. Prepare standard (1:2 dilution) in duplicate ranging from 32 ng/mL to 0.5 ng/mL. Add 100 µL of Sample Diluent, in duplicate, to the blank wells. Add 80 µL of Sample Diluent, in duplicate, to the sample wells. Add 20 µL of each Sample, in duplicate, to the designated wells. Add 50 µL of diluted biotin-conjugate to all wells, including the blank wells. Cover with a plate cover and incubate at room temperature, on a microplate shaker at 100 rpm if available, for 2 hours. Remove plate cover and empty the wells. Wash microwell strips 3 times as described in step 2. Add 100 µL of diluted Streptavidin-HRP to all wells, including the blank wells. Cover with a plate cover and incubate at room temperature, on a microplate shaker at 100 rpm if available, for 1 hour. Remove the plate cover and empty the wells. Wash microwell strips 3 times as described in step 2. Proceed to the next step. Pipette 100 µl of mixed TMB Substrate Solution to all wells, including the blanks. Incubate the microwell strips at room temperature (18° to 25°C) for about 15 minutes, if available on a rotator set at 100 rpm. Avoid direct exposure to intense light. The point, at which the substrate reaction is stopped, is often determined by the ELISA reader. Many ELISA readers record absorbance only up to 2.0 O.D. Therefore, the color development within individual microwells must be watched by the person running the assay and the

substrate reaction stopped before positive wells are no longer properly detectable. Stop the enzyme reaction by quickly pipetting 100 µL of Stop Solution into each well, including the blank wells. It is important that the Stop Solution is spread quickly and uniformly throughout the microwells to completely inactivate the enzyme. Results must be read immediately after the Stop Solution is added or within one hour if the microwell strips are stored at 2 - 8°C in the dark. Read the absorbance of each microwell on a spectrophotometer using 450 nm as the primary wavelength.

4.2.3. Molecular Docking

The crystal structure of EGFR complexed with erlotinib (PDB ID: 1M17) and $BRAF^{V600E}$ (PDB ID: 3OG7) were downloaded from the Protein Data Bank. Structure of compound **8d** was drawn and optimized using Marvin Sketch and Avogadro molecular editors. The protein was prepared using Autodock tools where the co-crystallized ligands and water molecules were removed then kollman charges and polar hydrogens were added. The grid dimensions for tubulin were set to 80x80x80. Autodock vina was used for molecular docking and the best docking poses were visualized using Discovery Studio Visualizer

4.2.4. ADMET prediction

The absorption, distribution, metabolism, excretion, and toxicity (ADMET) profile of compound **8d** was predicted using ADMETlab 3.0 (https://admetmesh.scbdd.com/), an integrated online platform that combines large curated datasets with multi-task graph neural network models for drug property assessment. The canonical SMILES of **8d** was

submitted to the server, and predictions were generated across a wide range of pharmacokinetic and toxicity endpoints.
